# Computational models for predicting bone fracture healing: a review of modeling approaches, predictions, and emerging strategies

**DOI:** 10.1007/s10237-026-02061-x

**Published:** 2026-04-03

**Authors:** Diogo G. Pires, A. Completo, Marco P. Soares dos Santos

**Affiliations:** 1 Department of Mechanical Engineering, Centre for Mechanical Technology & Automation (TEMA), University of Aveiro, Aveiro, 3810-193 Portugal; 2 Intelligent Systems Associate Laboratory (LASI), Guimarães, 4800-058 Portugal

**Keywords:** Bone fracture healing, Bone remodeling, Computational modeling, FEM, Healing simulation, Artificial intelligence

## Abstract

Background and Objective: The development of computational models for predicting bone fracture healing process holds strong potential to optimize therapeutic management in non-unions and delayed healing, reducing healthcare costs and disability-adjusted life years. The main goal of this study is to provide a thoroughly comparative analysis to computational models already proposed to predict fracture healing, including methodologies, mathematical frameworks, validation techniques, and comparative findings across different studies. Methods: This review analyzes 60 computational models selected through a systematic search in the Scopus database (2000–2025) using targeted keywords and rigorously screened according to predefined inclusion and exclusion criteria for simulating bone fracture healing, focusing on mechanical, biological, mechanobiological, ultrasound, and bioelectronic dynamics, employing FEM and artificial intelligence techniques. The effectiveness of each model in predicting healing progression was assessed by analyzing their computational frameworks, accuracy, and limitations. Results: Comparative analysis revealed that both mechanical and biological models provide fundamental predictions related to fracture healing (e.g., stress distribution and vascularization), but they often lack the physiological complexity demanded for clinical application. Mechanobiological models accurately predict tissue differentiation by combining mechanical stimuli, with strong qualitative agreement with in vivo histological patterns. Ultrasound models have been effective for non-invasive structural assessment, despite existing limitations due to simplified boundary conditions. Notably, the development of bioelectric models has been demonstrating a highly sensitive approach for assessing fracture healing. Conclusions:This study highlights that multidomain computational frameworks combining mechanobiological dynamics with dielectric properties hold significant potential to personalize the clinical management of delayed bone healing.

## Introduction

The number of bone fractures has exceeded 178 million per year (Wu et al. 2021), of which the non-union rate can reach 10% (Wildemann et al. 2021). It is among the most common injuries worldwide and is associated with high treatment costs, reduced socioeconomic productivity, and long-term disability. Indeed, recent reports highlight that the number of years lived with disability (YLD) currently exceeds 25.8 million years, as well as the significant increase in the number of fractures at active age groups (Wu et al. 2021; Cauley 2021). Therefore, the development of high-sophisticated technologies and advanced medical protocols to significantly enhance the healing management of the bone fracture healing process requires an accurate prediction of the underlying mechanisms.

Bone, as a living tissue, has the remarkable ability to self-heal (Duda et al. 2023). Clinically, the repair process occurs via one of two distinct pathways: primary and secondary healing. Primary bone healing occurs when bone fragments are strongly attached together under compression (e.g., hard internal fixation), allowing osteoblast and osteoclast activity to repair the bone directly without the creation of callus (Ghiasi et al. 2017). Differently, secondary bone healing is clinically the most used healing process, occurring under conditions of relative stability, when low-magnitude interfragmentary motion promotes the creation of a callus, leading to bone regeneration by intramembranous and endochondral ossification (Ghiasi et al. 2017). It is important to highlight that most computational models analyzed in this review are focused on simulating secondary fracture consolidation due to its common occurrence, multiphase nature, and mechanosensitivity. Following the secondary healing, a sequential formation of different tissues occurs through distinct stages, namely hematoma formation, soft callus, hard callus, and, finally, remodeling (Ghiasi et al. 2017). These stages are influenced by multiple biochemical and mechanical factors, which interact to create the conditions necessary for successful repair (Ghiasi et al. 2017). Included is the load and fracture stability characterizing the mechanical environment (Augat et al. 2021), as well as inflammatory cytokines, bone morphogenetic proteins (BMPs), and vascular endothelial growth factor (VEGF) characterizing the biochemical environment (Dimitriou et al. 2011; Cai et al. 2024). Interactions between mechanical and biochemical stimuli play a crucial role in guiding cellular recruitment, differentiation, and organization, shaping the quality and pace of bone healing (Augat et al. 2005). However, the repair process may fail, leading to non-unions or delayed recovery (Wu et al. 2021), due to a combination of geometric, mechanical, and biological factors, namely fracture gap size and alignment, instability or insufficient load transfer, and poor vascularization (Watanabe et al. 2013; Hankenson et al. 2011). Such conditions need in-depth understanding of how the different mechanisms interact, highlighting the importance of advanced models and computational approaches integrating multiphysical phenomena.

**Fig. 1:**
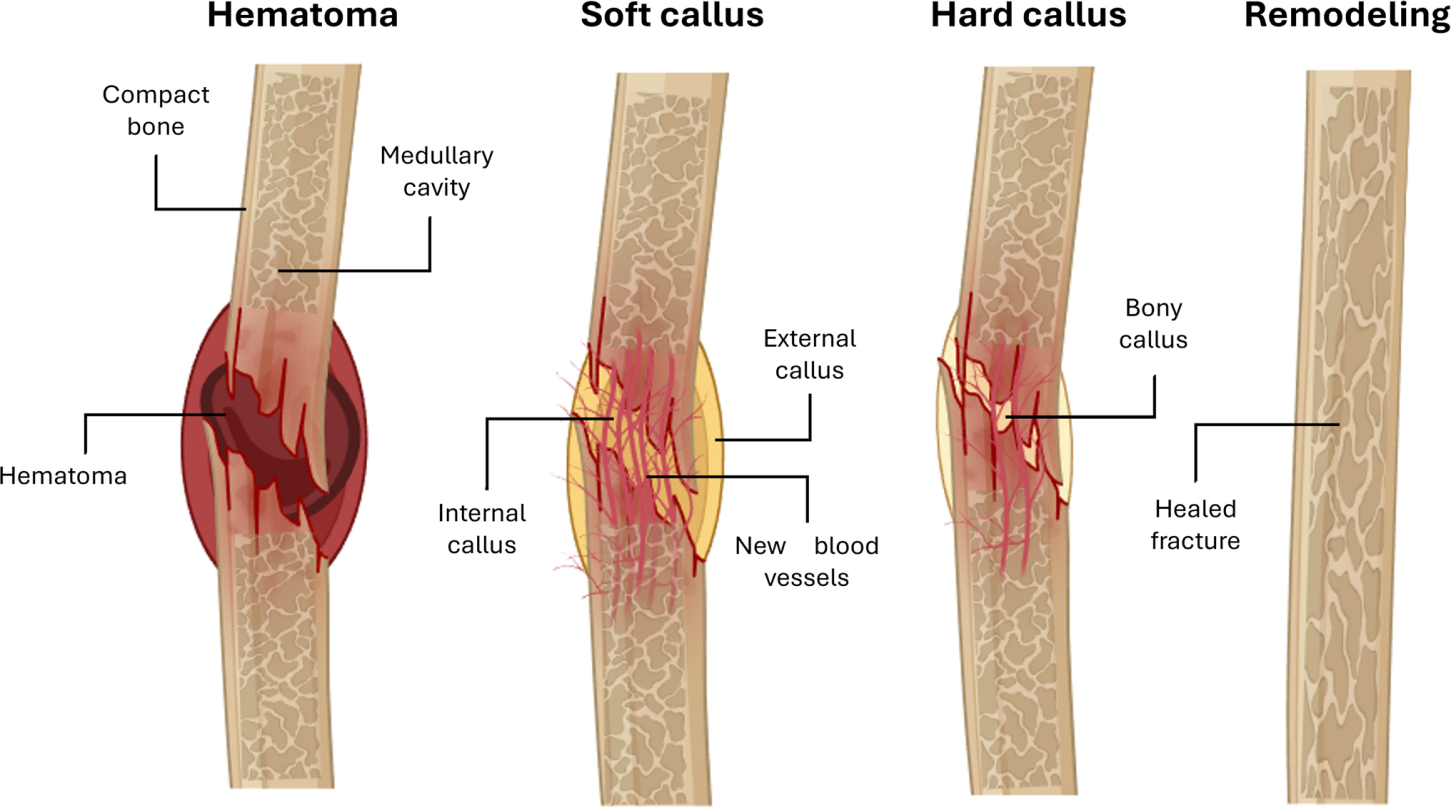
Illustration of the four bone fracture healing stages

Bone healing is a dynamic and highly coordinated process that unfolds in overlapping stages, involving a wide array of cells, signaling molecules, and structural transformations. It begins with the formation of a hematoma immediately after the fracture, which results from the vascular disruption at the injury site (inflammatory stage). This hematoma is rich in inflammatory cells and cytokines (such as TNF-$\alpha$, interleukins, VEGF, and BMPs), which initiate the healing process and recruit mesenchymal stem cells (MSCs) from the periosteum, endosteum, and circulation. Then, granulation tissue forms (soft callus stage), providing provisional mechanical stability and a scaffold structure for further cellular infiltration and angiogenesis. MSCs differentiate into chondrocytes and osteoblasts under the influence of local signals, leading to the formation of a calcified callus of immature bone (hard callus stage). Through endochondral ossification, this callus is gradually replaced. In the final stage, bone remodeling occurs over months to years, during which osteoclasts and osteoblasts remodel the immature bone into mature lamellar bone, restoring the original structure and strength (remodeling stage). This remodeling is modulated by mechanical forces, vascularization, and molecular cues, ensuring that the regenerated bone adapts to functional demands (ElHawary et al. 2021; Steppe et al. 2023; Sheen et al. 2023) (Fig. 1).

Several theories and simulation models related to fracture healing have been proposed in recent years to describe the most relevant mechanisms regulating bone morphogenesis and mechanotransduction, such as the mechanoregulation theory proposed by Claes and Heigele (1999), which suggests that different mechanical stimuli, including interfragmentary strain and hydrostatic pressure, influence the differentiation of mesenchymal cells into specific tissue types, such as bone, cartilage, or fibrous tissue (Claes and Heigele 1999). Another approach is the biphasic model proposed by Prendergast et al. (1997), which combines solid and fluid mechanical stimuli to predict tissue differentiation at the fracture site, based on cellular and extracellular matrix deformation patterns (Prendergast et al. 1997). These approaches, along with more recent finite element-based mechanobiological simulations and artificial intelligence computing, aim to improve the accuracy of bone healing predictions and contribute to the development of new technologies and therapeutic strategies.

This review provides a thoroughly comparative analysis to computational models already proposed to predict fracture healing, including methodologies, mathematical frameworks, validation techniques, and comparative findings across different studies, highlighting similarities and differences, and strengths and limitations, such that the effectiveness of each model predicting the progression of fracture healing can be assessed. The ultimate aim of this work is to contribute to minimize the societal impact of fracture healing, by evaluating which models are able to accurately provide predictions of mechanical, biological and electrical phenomena related to bone fracture healing, such that new scientific findings can support innovative clinical applications, including the design of bioelectronic implants and advanced techniques for management of non-unions and delayed healing (Pires et al. n.d., 2025; Quinn et al. 2025).

## Methods

### Selection criteria

A rigorous analysis of different computational approaches and algorithms for simulating the bone fracture healing was conducted. It examines the most relevant biological, mechanical, ultrasound, and electric models to predict the tissue differentiation process. The Scopus database was searched in the time interval between 2000 and 2025 by seeking for the terms in the title, abstract, or keywords “fracture AND bone AND healing”, OR “bone remodeling”, AND “comput* model”, OR ”FEM AND model”. Research results were further refined to fit the scope of this review, according to the following criteria: (i) papers published as full-length articles in peer-reviewed publications; (ii) papers reporting the use of mathematical models or computational simulations to address the bone fracture healing dynamics; (iii) papers reporting the simulation of at least one fracture healing stage (hematoma formation, soft callus, hard callus, or remodeling); (iv) papers simulating the formation of different tissues throughout the repair process; (v) papers written in English. Additional exclusion of studies was performed according to the following criteria: (i) papers containing the term ”remodeling”, but not referring to fracture remodeling (*e.g.,* bone remodeling during life); (ii) papers focused on distraction osteogenesis (DO), as it involves bone lengthening through traction, rather than bone fracture scenario; (iii) papers lacking a mathematical framework establishing a fracture healing dynamics; and (iv) all the papers in the fourth quartiles, according to the Clarivate ranking, were removed, as we found they do not provide rigorous and sufficient content. The search was completed in June 2025, resulting in 236 studies. After applying the defined selection criteria, 60 articles were selected.

### Literature search strategy

The following data were extracted and analyzed from the selected collection of papers: (1) type of model used (e.g., analytical, finite element, artificial intelligence, or other computational approaches); (2) biophysical dynamics; (3) main characteristics, such as spatial dimension (1D, 2D or 3D), material properties, type of boundary, and initial conditions; (4) main findings, including accuracy results and validation tests, distinguishing between quantitative validation (e.g., experimental or imaging data) and qualitative assessments (e.g., visual comparison using CT images); and (5) strengths and limitations of each computational model.

## Results

### Overview

**Fig. 2:**
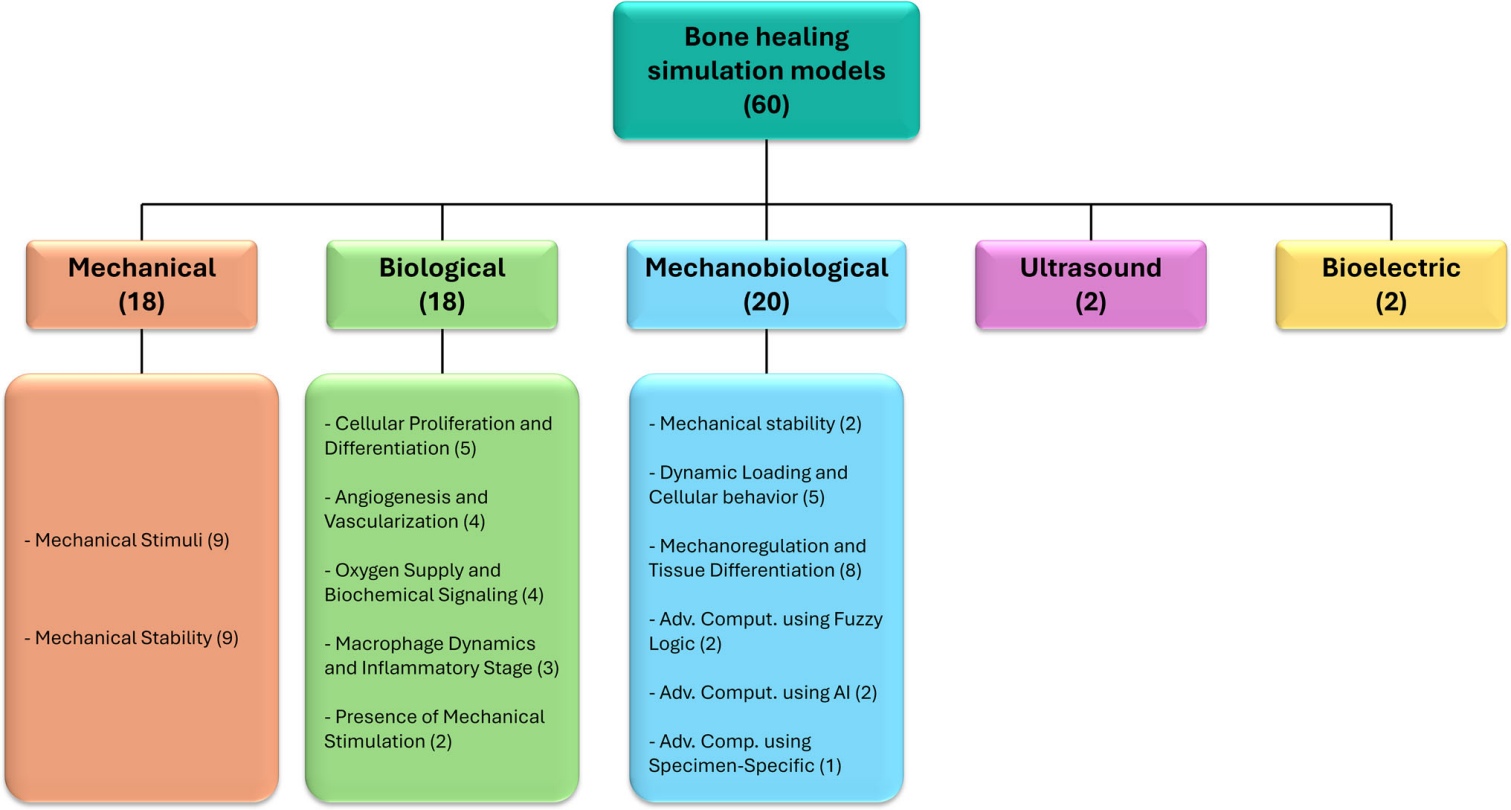
Schematic framework illustrating the categorization of computational models for bone fracture consolidation with the number of articles identified for each category

Five main biophysical models were found, namely predicting mechanical, biological, mechanobiological, ultrasound, and electric dynamics (Fig. 2). Each modeling approach describes specific phenomena related to fracture repair: (i) mechanical models aim to evaluate structural stability, stress distributions, and interfragmentary movement; (ii) biological models are mainly focus on cellular proliferation, angiogenesis, and biochemical signaling pathways; (iii) mechanobiological models combine the previous two described domains to mainly predict how mechanical inputs influence spatial and temporal tissue differentiation; (iv) ultrasound models are used to analyze wave propagation, in order to mainly provide predictions related to callus stiffening and geometric maturation; and (v) bioelectric models track temporal healing progression by predicting changes in tissue composition via dielectric changes. Following this general classification, the specific predictive capabilities and findings of each modeling approach are detailed below.

Mechanical computational models were primarily developed to predict stress distribution, stiffness, callus thickness, and interfragmentary movement. Reported results include up to 300% increase in local stress at one week near the cortex, predicted interfragmentary movement (IFM) reductions of 80% after 40 days for a 2 mm gap in agreement with *in vivo* data, and muscle loading effects increasing interfragmentary shear by up to 23% near the cortex and 11% in distant regions. The mechanical modeling approach was used to predict the effects on fractured tissues of mechanical stimuli, fixation stability, and tissue interactions during the healing phases. A total of 18 models were analyzed, among which FEM models stands out as a primary method for investigating mechanical factors during bone healing (Steiner et al. 2013; Ren and Dailey 2020; González et al. 2009; González-Torres et al. 2011; Wehner et al. 2014; Alierta et al. 2014; Ghiasi et al. 2019; Lipphaus and Witzel 2018; Paul et al. 2022; Miramini et al. 2015, 2018, 2019, 2023; Yang et al. 2024; Ganadhiepan et al. 2021, 2019; Suzuki et al. 2020; Travascio et al. 2021).

Biological modeling approaches focused on vascularization, biochemical signaling, and tissue distribution, predicting blood vessel surface fraction growth from 1.8% (week 1) to 41.0% (week 5), with low oxygen (< 5%) favoring chondrogenesis and high oxygen promoting osteogenesis. In summary, cell proliferation, angiogenesis, and oxygen supply regulation are all part of the numerous biological processes that contribute to fracture healing. A total of 18 studies have used computational simulations to predict these biological factors and their influence on the healing process, providing crucial details about how these cells and tissues respond to biochemical signals (Pietsch et al. 2018; Gómez-Benito et al. 2005; Sapotnick and Nackenhorst 2012; Isaksson et al. 2008; Grivas et al. 2013; Ganadhiepan et al. 2021; Son et al. 2014; Peiffer et al. 2011; O’Reilly et al. 2016; Burke and Kelly 2012; Borgiani et al. 2021; Cheng et al. 2021; Bailón-Plaza and Van Der Meulen 2001; Borgiani et al. 2023; Naveiro et al. 2021; Baratchart et al. 2022; Grivas et al. 2019; Vavva et al. 2018).

Mechanobiological models have been mainly developed to predict the synergetic impact between mechanical properties and cellular proliferation, differentiation, and extracellular matrix (ECM) deposition. This integrated approach is essential for optimizing therapeutic techniques and progressing the design of bioengineered implants. A total of 20 studies have used mechanobiological simulations to examine these interactions and their influence on bone regeneration (Wang and Yang 2019; Avval et al. 2016; García-Aznar et al. 2007; Ghimire et al. 2018; Irandoust and Müftü 2023; Byrne et al. 2007; Liu et al. 2022; Isaksson et al. 2006, 2008; Vetter et al. 2012; Repp et al. 2015; Quinn et al. 2022; Perier-Metz et al. 2022; Moore et al. 2014; Razavi et al. 2024; Ament and Hofer 2000; Morgan et al. 2024; Liu et al. 2023; Razavi et al. 2025; Lihai et al. 2017). Mechanobiological frameworks integrated mechanical and biological aspects, showing that optimal dynamic loading (10% strain at 1 Hz) increased cartilage formation by  15% and reduced fibrous tissue by  5%, promoting endochondral ossification. These models reproduced tissue distributions at day 7 with high accuracy (e.g., fibrous tissue: 90% endosteal, 93% cortical, 65% periosteal; cartilage up to 15%; bone 20% in periosteal zone) and matched *in vivo* callus infilling (predicted 90% vs. 87.5% measured).

Ultrasound-based approaches have also been employed to predict fracture healing progression by analyzing wave propagation through the callus, providing insights into tissue stiffness and composition. Two studies have demonstrated the potential of guided and longitudinal wave measurements to monitor and predict healing outcomes (Vavva et al. 2007; Protopappas et al. 2008). Ultrasound-based models identified increases in FAS velocity with callus stiffening (e.g., from 3360 m/s to 3900 m/s) and highlighted L(0,5) and L(0,8) guided wave modes as sensitive to geometry and tissue properties.

Bioelectric sensing approaches have recently begun to be explored to monitor bone healing by capturing changes in tissue dielectric properties over time. Two studies have shown that capacitance variations correlate with tissue composition, offering a new method to track healing progression (Pires et al. 2025; Conceição et al. 2023). Electric models have predicted capacitance variations decreases during healing, with variations dropping from 545.56 fF in inflammatory phase to 462.92 fF at the remodeling phase (3 mm fracture gap and 10 V), maintaining good sensitivity across fracture sizes (0.25–3 mm) and achieving strong correlation with tissue composition after normalization when compared with *in vitro* data ($\hbox {R}^{2} > 0.9$).

Overall, finite element mechanobiological models were the most frequently adopted, reflecting their ability to simultaneously capture the mechanical environment and the biological processes underlying bone regeneration, thus providing a more comprehensive and clinically relevant simulation framework.

### Computational models related to mechanical phenomena

#### Impact of mechanical stimuli

**Fig. 3:**
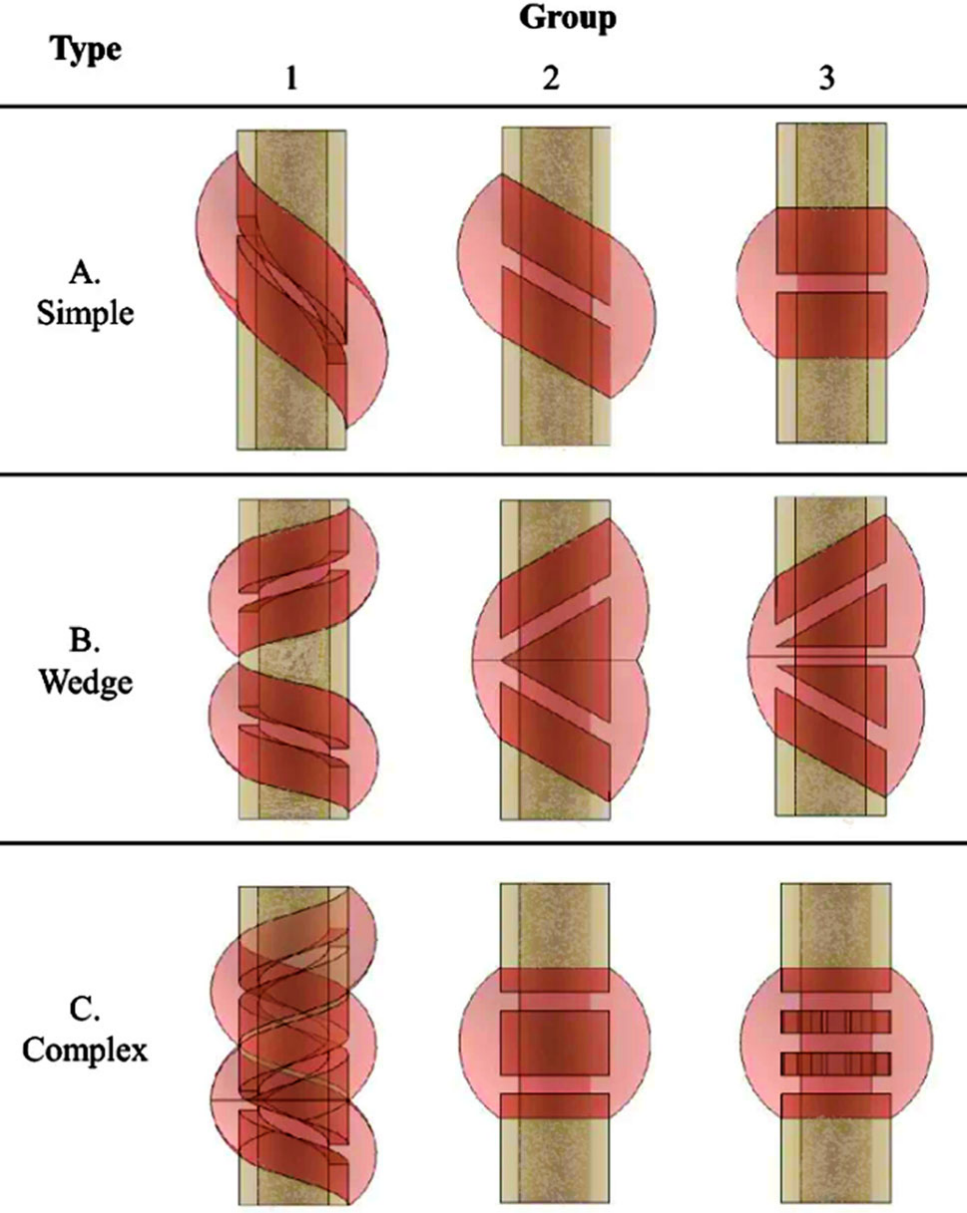
CAD models analyzed from different fracture scenarios: A1-Spiral; A2-Oblique; A3-Transverse; B1-Spiral Wedge; B2-Bending Wedge; B3-Fragmented Wedge; C1-Complex Spiral; C2-Segmental; C3-Irregular. Adapted from Ren and Dailey (2020)

Steiner et al. (2013) proposed a mechanoregulatory model for secondary fracture healing, incorporating distortional and dilatational strains as key stimuli for tissue differentiation. The 3D model was implemented in ANSYS (ANSYS Inc.) using 10-node tetrahedral elements and processed in MATLAB (v. 7.11, R2010b, MathWorks, Inc) using 20 fuzzy logic rules to regulate tissue differentiation. It is able to simulate intramembranous and endochondral ossification, cartilage formation, and revascularization, based on spatially and temporally varying strain patterns. Ovine tibial and metatarsal osteotomies were modeled, with initial fracture gaps ranging from 2 mm to 9.9 mm, and IFMs between 0.25 mm and 3.99 mm. Calibrated material properties included Young’s moduli of 538 MPa for bone, 28 MPa for cartilage, and 1.4 MPa for connective tissue. Quantitative validation was performed by minimizing the deviation between simulated and experimental IFMs across four *in vivo* loading cases, reaching an optimal error of 0.39. Qualitative validation against histological data at weeks 4 and 8 showed good agreement with physiological healing patterns. Model adjustments were required to reduce excessive predictions of intramedullary cartilage, although discrepancies remained under torsional loading. Limitations include the absence of explicit callus growth modeling and the assumption of a fixed healing domain; besides, intramedullary bone formation was observed under torsional loading, which was not observed during *in vivo* studies. Nevertheless, the model demonstrated strong predictive ability across mechanically distinct scenarios, which was further corroborated in five additional axial scenarios and one bending scenario. Using a similar fuzzy logic framework, Ren and Dailey (2020) extended the model’s application to more complex fractures. Their approach was used to test different fracture geometries, intramedullary nail fixation, and multiaxial loading conditions, including axial and torsional forces (Fig. 3). Simulation results indicated that torsional instability consistently led to delayed healing across all fracture types. Indeed, under combined axial and torsional loading, less than 1% of healing was predicted after 70 days, whereas purely axial loading resulted in full recovery. Additionally, torsional loads, combined with specific fixator configurations, were shown to induce non-union, particularly due to high distortional strain within the callus. Their model was implemented in ANSYS (version 17.2, ANSYS Inc.), using a 3D finite element framework, but the mechanical stimulus was computed using the Fuzzy Logic toolbox from MATLAB (MathWorks, Inc) to predict tissue differentiation based on local strain conditions. While this study provided relevant predictions into the role of loading and fracture geometry, validation was limited to qualitative comparison with previous computational results; besides, biological factors, such as those related to vascularization and inflammation, were not explicitly modeled.

González et al. (2009) developed a poroelastic boundary element (BEM) callus model to mimic mechanically induced tissue formation, emphasizing the impact of fluid flow and hydrostatic pressure at the fracture region. Their model incorporates local strain and pore pressure as key biophysical stimuli, with particular emphasis on the roles of hydrostatic pressure and interstitial fluid flow in guiding tissue formation. Tissue differentiation thresholds were adapted from Claes and Heigele’s quantitative theory (Claes and Heigele 1999), assuming intramembranous ossification occurring under strain magnitudes below 75% and hydrostatic pressures lower than 0.15 MPa, while considering that higher compressive pressures promote endochondral ossification. Such model was developed using a 3 mm osteotomy gap and 31% interfragmentary strain under a 500 N axial load. Axisymmetric geometry and idealized boundary conditions were applied, assuming isotropic and homogeneous tissue properties. Simulations allowed to predict that early healing stages are characterized by high pore pressures and strain magnitudes near the periosteum, promoting ossification. As healing progresses, both parameters stabilize, which promotes bone formation. The validation was obtained through qualitative comparison with *in vivo* and FEM simulations of interfragmentary movement and pressure distribution from other studies (Claes and Heigele 1999). Assuming idealized boundary conditions and isotropic, homogeneous media, their model oversimplifies the biological environment. Indeed, fracture healing involves asymmetric geometries and the differentiation of multiple tissue types, introducing both anisotropy and heterogeneity (Bigham-Sadegh and Oryan 2015). González-Torres et al. (2011) developed a similar model to assess how residual stress can impact fracture healing and remodeling. Their study simulated the evolution of residual stresses in a sheep metatarsus with a 3 mm osteotomy, stabilized with a ring fixator. Residual stresses were quantified by comparing stress distributions in the callus under loading alone (500 N axial cyclic load at 1 Hz) versus combined loading and growth. Results allowed to predict that within a week, residual stresses increased up to 300% in periosteal regions near cortical bone and up to 200% in the interfragmentary space compared to load-only conditions. In the fourth week, the influence of growth-induced stress diminished, affecting smaller regions. While the model reveals the significance of residual stress in early healing, particularly in periosteal regions, it lacks experimental validation and dynamic callus remodeling.

**Fig. 4:**
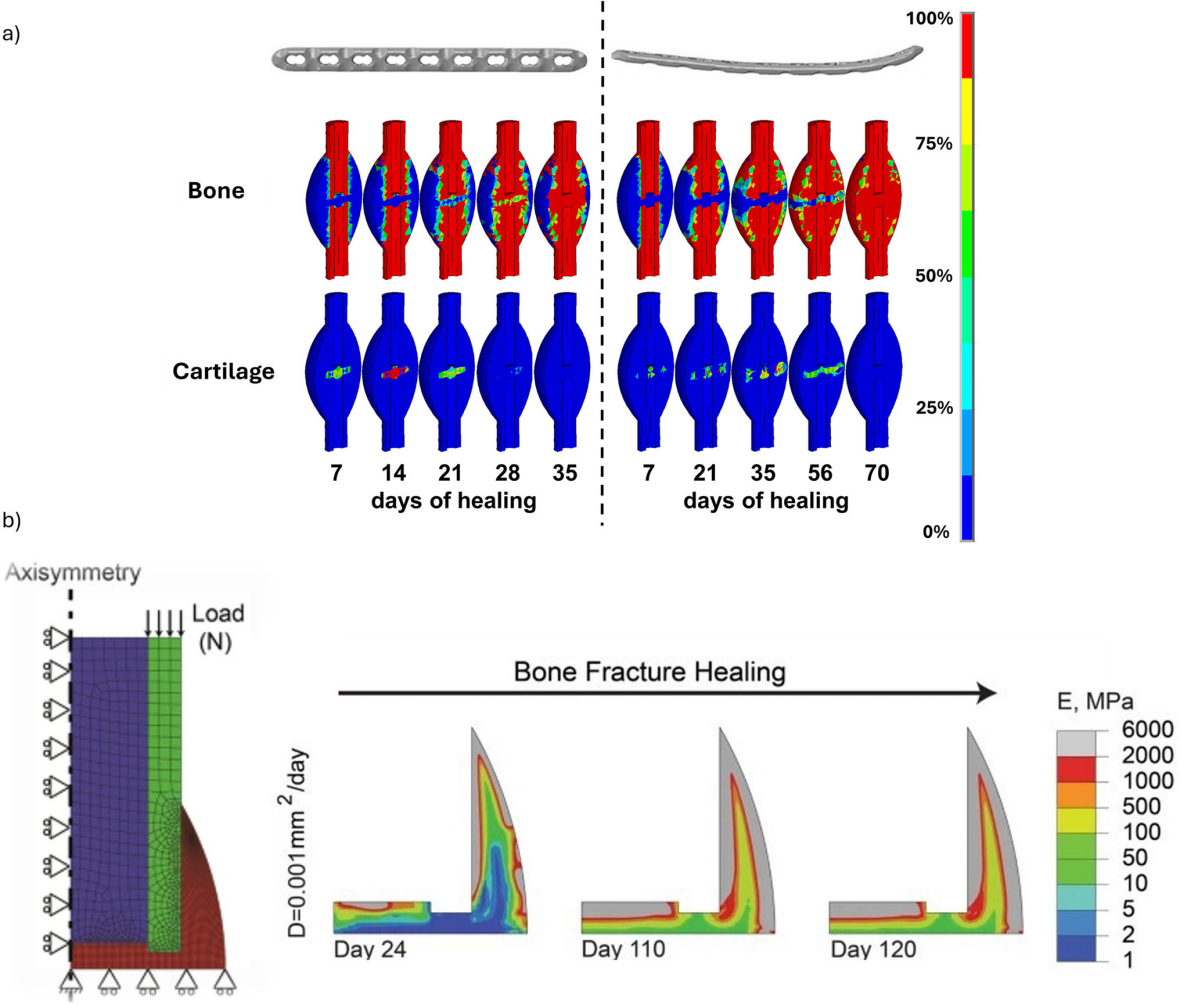
**a** Spatial distribution of bone (top) and cartilage (bottom) tissue fractions under rigid (left) and flexible (right) fixation. These results supported model calibration by linking tissue differentiation thresholds to predicted callus stiffness evolution. Adapted from Wehner et al. (2014). **b** Bone healing prediction of one-quarter of the callus region using the FEM model developed by Ghiasi et al. (2019). Adapted from Ghiasi et al. (2019)

Wehner et al. (2014) developed a finite element model in ANSYS (version 14.0, ANSYS Inc., Canonsburg, PA, USA) to simulate the time-dependent fracture healing in rats by predicting callus stiffness under rigid and flexible fixation. Their model was calibrated using three geometries, representing different healing stages and compared with *ex vivo* bending stiffness data. Material properties were identified as cortical bone (15,750 MPa), woven bone (1000 MPa), cartilage (5 MPa), and connective tissue (1 MPa). Tissue differentiation followed rules based on volumetric and distortional strains, with threshold values optimized via Latin Hypercube Sampling across 600 simulations (Fig. 4a). Exceeding 140 000 tetrahedral elements, the designed model was validated using 21 *in vivo* experiments with different fixation stiffnesses (10–119 N/mm), bodyweights (250–550 g), and fracture gaps (0.5–5 mm). It accurately reproduced healing in 15 scenarios, even though showing errors in aged rats and defects $\ge$3 mm, errors that reach 45% of the intact bone stiffness. Limitations include the assumption of linear elastic, isotropic tissue behavior and inaccurate predictions for aged rats and larger fracture gaps.

Alierta et al. (2014) and Ghiasi et al. (2019) developed FEM models to predict fracture healing outcomes by simulating the interaction between mechanical stimuli and callus differentiation at the fracture site. Both models integrate mechanistic principles to represent the callus evolution, but focusing on different aspects: Alierta et al. (2014) studied the time-dependent union level across a cohesive fracture interface. They were able to successfully replicate IFM reductions of up to 80% over 40 days in compression tests and match experimental torsional failure thresholds within 2 days of observed data. Ghiasi et al. (2019) emphasized the modulatory role of the initial healing phase, being able to predict that optimal healing occurred within 33 days when granulation tissue stiffness was 1–2 MPa, and callus thickness ranged between 3 and 6 mm, with progressive maturation over time (Fig. 4b). Values outside these ranges were predicted to result in delayed healing or non-union. Despite their predictive ability under controlled mechanical scenarios, both models are limited to small or idealized fracture gaps, underestimating the impact of spatial heterogeneity of mechanical stimuli throughout the callus.

Lipphaus and Witzel (2018) developed an advanced FEM model integrating both callus formation and long-term bone remodeling based on mechanical stimuli. Using ANSYS (Mechanical 14.5, ANSYS Inc.), they simulated three non-displaced femoral shaft fractures and one dislocated fracture under time-dependent axial loading. Their model incorporated over 280,000 linear tetrahedral elements, with initial interfragmentary movements of 0.2, 0.6, and 1 mm. Their simulations were able to capture MSCs, differentiation, and the evolution of tissue stiffness, enabling accurate remodeling predictions. Healing outcomes were predicted to vary with initial movement: bone union occurred after 2, 8, and 10 weeks for increasing gap sizes, with final compressive stresses of 13 MPa matching physiological values. Although good agreements were obtained between predicted healing trends and both clinical data and data from previous numerical studies, limitations include the use of linear elastic materials, absence of poroelastic behavior (important for torsional or frequency-dependent loading (Müller and Witzel 1981)), and reliance on simplified fracture geometries.

Finally, Paul et al. (2022) developed a FEM model based on weekly *in vivo* micro-CT scans (10.5 $\upmu$m resolution) of 20 mice with femoral osteotomies to simulate bone regeneration under mechanical loading. The model linked local effective strain to tissue behavior (formation and resorption) across a bone mineral density range of 395–720 mg HA/cm^3^. Results allowed to predict that areas with high strain have a higher probability of forming new bone, with area under the curve (AUC) > 0.75 for bone formation, indicating good predictive performance. The correct classification rate (CCR) for predicting tissue behavior (formation, quiescence, resorption) was > 33% after week 3 (bone union phase). Despite its relevant strengths, the following limitations were found in such model: (i) it does not consider how the microscopic structure of tissues affects mechanical signals; (ii) it uses static loads, which do not reflect the realistic dynamic forces acting on the bone–fixator system; and (iii) the transition zones between bone and soft tissue can introduce errors when computing the strain per voxel (partial volume effect).

#### The role of fixation devices in mechanical stability

**Fig. 5:**
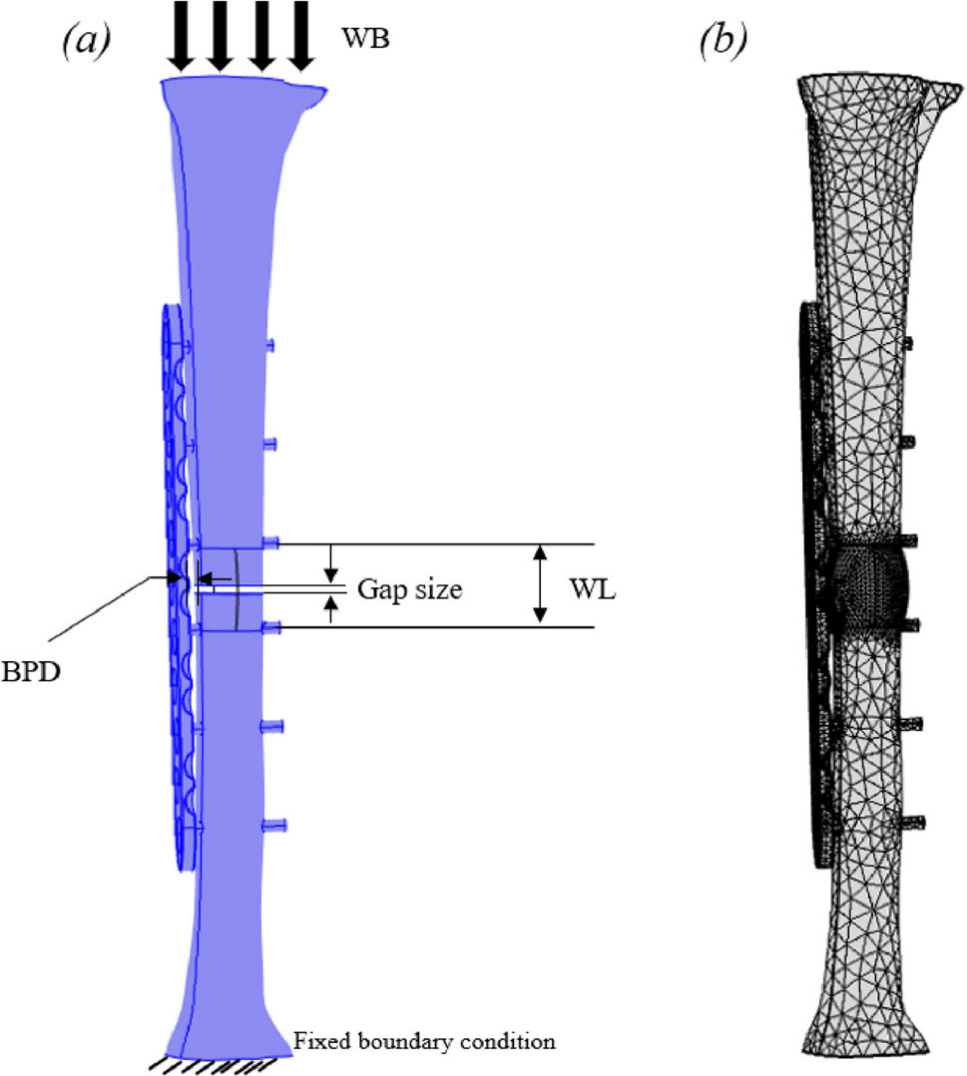
Schematic of the bone fracture configuration showing key mechanical parameters: weight-bearing load (WB), distance between bone and plate (BPD), working length (WL), and the fracture gap. **b** Finite element representation including the callus region at the fracture site. Adapted from Miramini et al. (2019)

Mechanical stability, which is mostly ensured by fixation devices, is a quite relevant requirement in bone repair (Sumita et al. 2003; Kim et al. 2020). Miramini et al. (2015) developed a FEM model to simulate secondary bone healing in a simplified 3D tibial fracture stabilized with an locking compression plate (LCP). Their model included a 2 mm fracture gap and mechanical stimuli. Results allowed to predict that excessively stiff fixations (e.g., with a short working length or small bone–plate distance) resulted in interfragmentary strains (IFS) < 2%, which promoted direct bone formation. However, they also found that excessive rigid fixations could suppress the formation of cartilaginous intermediate tissues, essential for endochondral ossification, potentially delaying healing. On the other hand, by increasing the plate flexibility via a larger working length (e.g., from 30 mm to 100 mm) or bone–plate distance (e.g., from 2 mm to 4 mm), IFSs can be increased > 10% in the far cortex, promoting fibrous tissue formation. In a follow-up study, Miramini et al. (2018), also developed a deterministic FEM model to investigate how the stiffness of locking plates influences bone healing. They applied a time-dependent solver from COMSOL MULTIPHYSICS (COMSOL AB) with 35,999 s-order tetrahedral elements for the fracture, 2158 for the LCP and 1375 for the screw domains. Results allowed to predict that constructs using standard locking screws (SLS) produced highly nonuniform interfragmentary strain (IFS), with particularly low values in the near cortex ($\approx$ 2% for stainless steel 4.5 mm LCPs), which is insufficient to stimulate cartilaginous callus formation. Differently, they also predict that the use of dynamic locking screws (DLS) significantly increased IFS at the near cortex (up to a fivefold improvement), promoting more favorable chondroblast differentiation. The ratio between the IFS in the far cortex and the one found in the near cortex ($\text {IFS}_{\text {FC}} / \text {IFS}_{\text {NC}}$) was reduced from 8–10 in SLS constructs to 2–3 in DLS configurations, indicating a substantially more uniform mechanical environment. The most favorable conditions for symmetric cell differentiation and indirect bone healing were observed in the stainless steel 4.5 mm LCP combined with DLS, due to its high bending stiffness and resulting uniform IFS distribution. Supported by this study, Miramini et al. (2019) introduced a probabilistic FEM framework for simulating early-stage healing of tibial shaft fractures. Their model incorporated statistical variability in input parameters, such as fracture gap size, bone–plate distance (BPD), working length (WL), weight bearing loading, assuming normal distributions for fracture gap size (mean ± SD: 2.5 ± 0.5 mm), callus stiffness (150 ± 30 MPa), and BPD (2 ± 0.4 mm) (Fig. 5). The authors evaluated the impact of these uncertainties on mechanical stimulus distributions using Monte Carlo simulations. Results allowed to predict that the healing trajectory was highly sensitive to variability in mechanical inputs, with the probability of achieving optimal mechanical stimuli decreasing significantly for higher BPD and WL values. Increasing BPD from 2 mm to 4 mm was found to reduce the probability of favorable healing stimuli by $\approx$ 40%. These findings emphasize the need to individualize fixation parameters and to consider parameter uncertainties during preoperative planning. More recently, Miramini et al. (2023) investigated the influence of muscle loading on fracture healing during static weight-bearing. They integrated outputs from a musculoskeletal model of the lower limb during quiet standing to establish the boundary conditions of their FEM model. This model improvement allowed to predict that ignoring muscle forces led to an underestimation of IFS by up to 25%. Under full weight-bearing and LCP configuration C1, muscle loads increased IFS in the near and far cortices by 23% and 11%, respectively. On the other hand, in a more flexible configuration (C4), including muscle loads reduced IFS by 20–23%, they observed that muscle-induced forces can affect the deformation mode of the LCP. The study also quantified the contribution of specific muscles, showing that the Quadriceps Femoris (Vastus Lateralis) produced forces around 38%, 31%, and 12% of the knee contact force in the lateral, anterior, and superior directions, respectively. Despite offering important insights, models developed by Miramini et al. (2023) present some limitations. Firstly, assumptions of linear, homogeneous tissue properties and uniform callus distribution reduce physiological accuracy. Besides, the model validation was not carried out no direct comparison with experimental or clinical data was performed, limiting the reliability of predictions. Lastly, the proposed models only simulate static or simplified loading conditions, failing to capture the effects of dynamic activities, such as walking, which are crucial in realistic fracture healing.

Yang et al. (2024) developed a computational model combining finite element analysis in ABAQUS (Dassault Systèmes) with a Python-based algorithm to simulate fracture healing under different mechanical loads and initial conditions. Their study performed 3315 simulations, with parametric variations, including fracture gap size (0.5–8 mm), initial Young’s modulus (0.05–1.55 MPa), and mechanical load (0.001–10 N). Results indicate that smaller fracture gaps ($\le$1 mm) and higher initial tissue stiffness ($\ge$1.05 MPa) accelerate healing by promoting direct bone formation and angiogenesis. Mechanical load was predicted to exhibit a nonlinear effects: moderate loads (0.1–2 N) enhanced healing, while very low or high loads impaired bone formation, favoring fibrous tissue differentiation or resorption. Qualitative predictions agree with clinical observations of fracture healing patterns. Limitations include fixed parameter ranges and simplified biological complexity. This study highlights the critical role of initial mechanical and biological conditions in fracture repair dynamics.

Several studies have further explored the impact of different mechanical environments, especially in the context of complex fracture fixations. Ganadhiepan et al. (2021) developed a probabilistic model to investigate bone regeneration under Ilizarov circular fixators (ICF), focusing on early-stage III healing (reparative phase). Using COMSOL Multiphysics (v 5.3a, COMSOL AB), they simulated the fracture healing of a transverse mid-diaphyseal ovine fracture, modeling bone and soft tissues as poroelastic materials. Four mechanical input variables (axial load, fracture gap size, ICF wire pretension, and wire diameter) were modeled as independent normal distributions with coefficients of variation (COV) ranging from 0.1 to 0.9. The probability of success (PoS), defined as the likelihood of average deviatoric strain remaining within the osteogenic range (0.005%–6%), was estimated through linear regression. Results allowed to predict high sensitivity of cortical callus to uncertainties in axial loading (PoS decreased to 46%) and gap size (decreased to 70%), while ICF related parameters was found to have minor influence (PoS > 95%). Despite offering relevant insights into mechanical variability, this model is limited by simplified geometry, and it only was qualitatively validated with other experimental studies. Similarly, Ganadhiepan et al. (2019) also developed a fully coupled 3D mechano-model of a transverse mid-diaphyseal ovine tibial fracture. This was simulated using a Taylor Spatial Frame (TSF), developed in COMSOL Multiphysics (v 5.3a, COMSOL AB) (after geometric preprocessing in SolidWorks (Dassault Systèmes)) to quantify the effects of fracture gap size (1, 3, and 5 mm), axial load (100–200N), wire pretension (491–1275N), and TSF ring diameter (130 vs 155 mm) on interfragmentary mechanics and MSC differentiation. Domains were meshed with 216 496 and 207 595 tetrahedral elements for 155 and 130 mm ring TSFs. This model was validated using ARAMIS 3D optical measurements, which allowed to verify that predicted IFMs fell within experimental error (maximum deviation 7%). A two-way ANOVA on the mechanical test data indicated that both axial load and ring diameter significantly influenced TSF axial stiffness (P < 0.05), with ring diameter employing the greatest effect, while the interaction between them was not statistically significant, even though it was close to the threshold (P = 0.06). Parametric simulations showed that reducing the fracture gap from 3 mm to 1 mm increased osteoblast content and decreased fibroblast content in the endosteal callus by around 35%. Increasing axial load from 150 N to 200 N was predicted to augment fibroblast differentiation by up to 60%, while osteoblast content decreased by up to 25% in the periosteal region. Decreasing TSF ring diameter from 155 mm to 130 mm conduct to an approximate 20% increase in chondrocyte content and a 15% reduction in fibroblast content. Similarly, increasing wire pretension from 883 N to 1275 N resulted in a 10% increase in chondrocytes and a 5% decrease in fibroblasts. Notably, significant changes in the endosteal callus were observed primarily at the smallest gap size (1 mm), whereas all parameters predominantly influenced the periosteal callus. These predictions highlight the distinct, site-specific mechano-responses within the fracture environment, highlighting the importance of patient-specific fixation and loading parameters in early bone healing. Limitations of the study include isotropic bone material assumptions, disregard of vascularization and biochemical regulatory factors, and restriction to axial compression loading, which confines predictions to the early reparative phase.

**Fig. 6:**
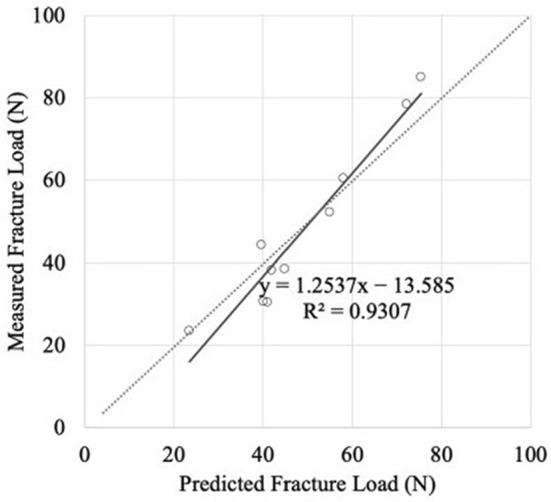
Relationship between measured and predicted fracture loads. Adapted from Suzuki et al. (2020)

Suzuki et al. (2020) developed a specimen-specific FEM approach to assess the biomechanics of callus during bone healing, focusing on estimating mechanical properties from CT-based density data. In 10 male New Zealand white rabbits, a 1 mm diaphyseal femoral defect was stabilized using an external fixator composed of resin cement and four 2 mm pins. At 3–5 weeks post-surgery, a total of 95 cuboidal callus samples (5 $\hbox {mm}^3$) were extracted for mechanical testing and quantitative CT scanning. CT data were processed using a software package (Mechanical Finder, Research Center for Computational Mechanics) to assign heterogeneous material properties based on Hounsfield unit (HU) values to quantify tissue densities. Histological analysis was used to link mineral density to tissue composition: their results allowed to predict that specimens with 0.310 mg/cm^3^ exhibited 35.5% cartilage and only 5.4% mineralized bone, while the ones with higher density (0.432 mg/cm^3^) showed reduced cartilage (8.5%) and increased bone mineralization (21.5%), indicating progressive ossification with maturation. The Young’s modulus and yield stress of each sample were experimentally measured and correlated with bone mineral density ($\sigma$), revealing strong nonlinear relationships ($r = 0.82$ and $r = 0.80$, respectively). The following empirical relations were found: $E = 0.2391e^{8\sigma }$ and $\rho = 30.49\sigma ^{2.41}$. A second cohort of six rabbits underwent identical surgical procedures for model validation purposes. Their femurs were scanned by CT and modeled in 3D using nonlinear FE analysis with Drucker–Prager plasticity. Predicted fracture loads from FEM (mean: 49.2 N) closely matched experimental values (mean: 48.1 N), with a high linear correlation ($r = 0.965$) (Fig. 6). This work demonstrated that CT-based FEM with specimen-specific calibration is able to provide accurate estimations of callus strength during healing, supporting its use for individualized clinical decision-making. Limitations include small sample size, use of a type of animal model, and potential scaling differences when extrapolating to human healing.

Finally, Travascio et al. (2021) developed FEM models to investigate the mechanical performance and healing implications of various screw configurations in tibial diaphyseal fracture fixation. Their computational models comprised $\approx$ 92 000 tetrahedral elements incorporating bone fragments, plate, screws, and cartilaginous callus. 3-Matic (Materialize) and FEBio (v. 2.4.0, University of Utah) were used to mesh the model and to conduct the simulations, respectively. Results allowed to predict similar axial stiffness across all configurations, with statistically significant relative rotations observed only in the coronal plane. Maximum von Mises stresses in the implants ranged from 3 MPa to 15.2 MPa, with the C1368 configuration being found to exhibit the lowest stresses, and the fully screwed C8 configuration the highest stresses. Mechanistic analysis using the Mechanical Stimulus Index (MSI) revealed that configurations C1368 and C1458 produced MSI values within the ideal osteogenic range (1 < MSI < 3) at peak loading, whereas C8 remained below this threshold. This study allowed to predict that the C1458 screw configuration is able to offer a favorable balance between mechanical stability and osteogenic stimulation, suggesting its potential as the preferred fixation strategy for tibial diaphyseal fractures. Model validation was performed in biomechanical tests on synthetic bone models, showing that the predicted interfragmentary rotations in the coronal plane were within the experimental mean standard deviation under compressive loads ranging from 300 to 1000 N. Limitations of this computational model include the use of synthetic bones and modeling of an idealized 2 mm transverse fracture, reducing its ability to be generalized to clinical scenarios.

#### Summary

A summary of model types, biophysical inputs, model characteristics, main predictions, validation method and limitations related to each study focused on mechanical modeling approaches is presented in Table 1.

**Table 1:** Summary of mechanical models developed to simulate bone healing processes, focusing on the physical forces influencing healing and the material properties applied to bone during regeneration

Model type	Biophysical inputs	Model characteristics	Main predictions	Validation method	Limitations	Reference
3D FE + Fuzzy Logic	Distortional strain, dilatational strain (mechanical stimuli)	3D finite element model in ANSYS using 10-node tetrahedral elements; fuzzy logic with 20 rules in MATLAB to regulate tissue differentiation; fracture gaps 2−9.9 mm	Model predicts tissue differentiation based on strain patterns	Quantitative: minimizing deviation between simulated and experimental IFMs over time; Qualitative: comparison of predicted tissue distributions with histology at weeks 4 and 8	No explicit callus growth; the model predicts intramedullary bone formation, which was not observed in *in vivo* studies	Steiner et al. (2013)
3D FE + Fuzzy Logic	Distortional strain, axial and torsional loading, fracture geometry, fixation method (intramedullary nail)	3D finite element model in ANSYS; fuzzy logic in MATLAB to predict tissue differentiation from local strain; tested various fracture geometries and fixation techniques	Torsional instability delays healing in all fracture types; combined axial and torsional loads cause <1% healing after 70 days vs full recovery with axial only; torsional loads plus fixators induce non-union via high distortional strain in callus	Qualitative comparison with previous computational models	Limited validation; no biological factors included	Ren and Dailey (2020)
Poroelastic BEM model	Strain magnitude, pore pressure, hydrostatic pressure and interstitial fluid flow	Axisymmetric poroelastic BEM assuming isotropic and homogeneous; 3 mm osteotomy gap, 500 N axial load, 31% interfragmentary strain	High pore pressure and strain near periosteum promote ossification in early healing; model captures pressure and strain evolution over time; supports poroelastic regulation of tissue differentiation	Comparison with *in vivo* and FEM data for interfragmentary movement and pressure patterns	Simplified geometry and static conditions; no modeling of biological processes	González et al. (2009)
FEM	Axial mechanical load (500 N, 1 Hz), growth-induced strain, residual stress	2D axisymmetric model of sheep metatarsus with 3 mm fracture gap and ring fixation; 4 tissue types with fixed properties; simulations at 1 and 4 weeks post-fracture	Residual stresses influence mechanical environment: up to 300% increase in stress at 1 week near cortex; reduced effect at 4 weeks	Comparisons with other studies used for reference	Static geometry, no remodeling, no biological activity modeled	González-Torres et al. (2011)
FEM	Volumetric and distortional strain, bodyweight, fixation stiffness, gap size	3D FEM in ANSYS (+140k tetrahedral elements); cortical bone (15,750 MPa), woven bone (1000 MPa), cartilage (5 MPa), and connective tissue (1 MPa)	Accurately predicted callus stiffness in 15/21 *in vivo* rat studies; effective across variable mechanical conditions; poor accuracy for large gaps or aged rats	Quantitative comparison with *ex vivo* stiffness from 21 rat experiments	Assumes isotropic linear elasticity; limited fracture gaps and rats age	Wehner et al. (2014)
FEM	Normal and shear strain, fixation stiffness, axial and torsional loads	3D FE model in ABAQUS; 91,558 elements; evolving union degree $\alpha$; intramembranous and endochondral healing included	Predicted IFM reduction of 80% after 40 days (2 mm gap); torsional failure timing matched experimental data (within 2 days)	Compared with *in vivo* experiments (sheep/human); quantitative match of IFM, stiffness, and torque resistance	Limited to small gaps; fixed geometry	Alierta et al. (2014)
FEM	Tissue stiffness (0.01–2 MPa), callus thickness (1–8 mm), gap size (0.5–4 mm), fluid flow	2D axisymmetric poroelastic FE model in ABAQUS (v6.13); $\approx$2,700 elements; iterative healing over 120 days; MSC migration via diffusion model	Optimal healing in 33 days for E = 1–2 MPa, callus = 3–6 mm; healing delayed or failed outside this range	Matches experimental data on healing timeline, callus development, and MSC distribution; comparison with the literature	Fixed geometry post-initial phase; assumes homogeneous tissues	Ghiasi et al. (2019)
FEM	Intermediate connective material and ossification regulated by stress (ranging from -13 to +10 kPa)	Iterative FE in ANSYS 14.5; 278–284k tetrahedral elements; initial IFMs = 0.2–1 mm; axial loading up to 6.4 kN	Bone bridging at 2–10 weeks depending on IFM; final compressive stress $\approx$ -13 MPa; callus index 1.2–1.4; remodeling reduces stress concentrations	Qualitative comparison with clinical/animal data; healing time and bone architecture consistent with known outcomes	Linear elastic model; no poroelasticity; simplified geometries	Lipphaus and Witzel (2018)
FEM	Tissue formation, resorption, and mineralization regulated by effective strain (normalized); mechanosensitivity varies over time	Micro-FE (Parasol); $\approx$4.9M voxels; combined axial + bending loads (8–16 N); mineral density 395–720 mg HA/$\hbox {cm}^3$	Bone formation at high strain; resorption at low strain; AUC > 0.75 for formation/mineralization; CCR > 33% indicates mechanoregulation	Comparison with *in vivo* micro-CT in 20 mice over 7 weeks; voxel-wise validation via time-lapse registration	Static loading; no dynamic or cellular-scale modeling; partial volume effects at interfaces	Paul et al. (2022)
FEM	Tissue differentiation regulated by interfragmentary strain (IFS)	3D tibial fracture with 2 mm gap, LCP fixation; variable working length (30–100 mm) and bone–plate distance (2–4 mm); mechanical loading conditions mimicking axial and bending forces	Excessively stiff fixations (short WL, small BPD) cause IFS < 2%, favoring direct bone formation but suppressing cartilaginous tissue; increased flexibility (larger WL, BPD) increases IFS > 10%, promoting fibrous tissue formation	Comparison with experimental data from sheep fracture models; qualitative agreement with clinical observations	Simplified geometry; no cellular-scale modeling; limited dynamic loading scenarios	Miramini et al. (2015)
FEM	Fracture gap size, locking plate stiffness, screws	COMSOL Multiphysics simulation with 35,999 (fracture), 2,158 (plate), and 1,375 (screw) second-order tetrahedral elements; 150N axial load ramped over 0.5s	Best conditions for were observed in the stainless steel 4.5 mm LCP combined with DLS	Qualitative agreement with experimental trends and published data	Mechanobiological simplifications; uncertain biological input	Miramini et al. (2018)
Probabilistic FEM	Fracture gap (2.5 ± 0.5 mm), callus stiffness (150 ± 30 MPa), BPD (2 ± 0.4 mm)	Statistical variability via Monte Carlo and Latin Hypercube; stimuli-driven differentiation	Healing sensitive to BPD and WL; BPD increase reduces favorable stimuli probability $\approx$40%	Compared with experiments and deterministic models	Assumed normality and independence; early healing focus; needs further validation	Miramini et al. (2019)
FEM	Interfragmentary strains (IFS) under full and partial weight-bearing; muscle load effects; LCP configurations (bone–plate distance and working length)	COMSOL Multiphysics FE model with tetrahedral mesh; 15 muscles represented; musculoskeletal model in OpenSim v3.3 with 92 muscle-tendon units	Muscle loading increased IFS by up to 23% (near cortex) and 11% (far cortex); muscles contribute up to 38% of knee contact force; LCP configuration influences stiffness and IFS	Model based on participant gait and force data; material properties from the literature	Single participant data; static loading (quiet standing); simplified material and load assumptions	(Miramini et al. 2023)
FEM + Algorithm	Fracture gap size, initial Young’s modulus, mechanical load, max swelling radius, angiogenesis inhibition strain threshold	Integrated finite element analysis (ABAQUS) coupled with Python-based biological algorithm; 3315 simulations varying 7 parameters	Smaller fracture gaps and higher initial Young’s moduli accelerate healing; moderate mechanical loads promote healing; angiogenesis parameters strongly affect healing	Comparison with clinical healing trends and known biological behavior	Simplified mechanical loading (constant external stimuli), no full biological complexity, reliance on fixed parameter ranges	(Yang et al. 2024)
FEM + Probabilistic analysis	Fracture gap size, axial loading (weight-bearing), wire pretension, wire diameter	Finite element model developed in COMSOL; probabilistic approach to assess mechanical microenvironment during stage III healing; input uncertainties modeled with normal distributions; PoS calculated as success metric	Cortical callus healing highly sensitive to axial loading and fracture gap size; wire pretension and diameter less impactful; PoS drops significantly with increased uncertainty in loading	Validation limited to qualitative comparison with other studies	Simplified bone geometry (cylindrical), axial load only, independent inputs assumed, no full healing stages considered	(Ganadhiepan et al. 2021)
FEM	Fracture gap size (1, 3, 5 mm), axial load (100–200 N), wire pretension (491–1275 N), TSF ring diameter (130, 155 mm)	COMSOL Multiphysics meshed with 216 496 and 207 595 tetrahedral elements for 155 and 130 mm ring TSFs	Gap 3$\rightarrow$1 mm: +35% osteoblasts, -35% fibroblasts (endo); Load 150$\rightarrow$200 N: +60% fibroblasts, -25% osteoblasts (peri); Ring 155$\rightarrow$130 mm: +20% chondrocytes, -15% fibroblasts (peri); wire tension 883$\rightarrow$1275 N: +10% chondrocytes, -5% fibroblasts (peri); endosteal effects mainly at 1 mm gap	ARAMIS 3D optical measurements; IFMs within 7% error	Assumes isotropic bone; no vascular or biochemical effects; axial compression only; early reparative phase focus	(Ganadhiepan et al. 2019)
Specimen-specific FEM	CT-derived mineral density; histological tissue composition	FEM implemented in Mechanical Finder; Drucker–Prager plasticity; nonlinear elastic–plastic behavior; empirical equations fitted from 95 samples	$E = 0.2391e^{8\sigma }$, $r=0.82$; $\rho = 30.49\sigma ^{2.41}$, $r=0.80$; cartilage decreased and mineralized bone increased with density; accurately predicted fracture loads	Experimental fracture load in 6 rabbits compared to FEM predictions: $r=0.965$, 48.1 N (exp.) vs. 49.2 N (FEM)	Small sample size; animal model; applicability to humans uncertain	(Suzuki et al. 2020)
FEM	Compressive loading (300–1000 N); callus tissue properties; implant stress; MSI (Mechanical Stimulus Index)	$\approx$92 000 tetrahedral elements; 3 screw configurations (C8, C1368, C1458); 2 mm transverse tibial fracture; mesh built in 3-Matic, simulations in FEBio	C1458 showed best balance: good rigidity and MSI within osteogenic range (1 < MSI < 3); C1368 showed lowest implant stress; C8 had lowest MSI and highest screw stress	Comparison of predicted interfragmentary rotation (coronal plane) with biomechanical data from synthetic tibia models (within mean±SD)	Synthetic bones used; idealized 2 mm transverse fracture; single loading direction; no soft tissue effects	(Travascio et al. 2021)

### Computational models related to biological phenomena

#### Cellular proliferation and differentiation

As cellular proliferation is an important component of bone fracture healing, Computational models have been developed to predict how differences in initial healing settings influence the behavior of bone-forming cells. Pietsch et al. (2018) developed a 3D computational framework that integrates a FEM model with a Coupled Level Set and Volume of Fluid (CLSVOF) interface-capturing technique to simulate early-stage bone fracture healing. This integrated model was designed to focus on regulated tissue differentiation and spatial front propagation by capturing moving interfaces between distinct tissue types. Implemented using a custom FEM solver (CSparse) and OpenFOAM (v. 4.1 OpenFOAM Foundation), an axisymmetric geometry of ovine metatarsus stabilized with an external fixator was used. 500 N of mechanical axial loading was applied, and tissue differentiation was governed by hydrostatic and distortional strain thresholds, constrained by biological factors, such as vascularization and cartilage age. Such model was able to predict both endochondral and intramembranous ossification, accurately reproducing tissue formation patterns and IFM trends. Validation was conducted by comparing simulation outputs with data from different studies: good agreements in tissue area fractions and IFM trajectories were obtained. A sensitivity analysis allowed to predict that ±10% variation in tissue growth velocities is able to significantly influence the healing progression. Despite its strengths, the model does not account for spontaneous tissue initiation due to the requirement of preexisting tissue boundaries; besides, it simplifies vascularization as a binary condition.

Gómez-Benito et al. (2005) developed a biological FEM model to investigate the influence of fracture gap size on the spatial and temporal progression of bone healing. Their model simulates a 2D axisymmetric cylindrical mid-diaphyseal fracture of a long bone, stabilized by an external fixator. It was implemented in ABAQUS (v.6.3 Hibbit, Karlsson and Sorensen, Inc.), coupled with adapted mathematical routines (García-Aznar et al. 2007) to update tissue properties throughout the healing process. Tissue differentiation was established to be governed by Prendergast’s mechanoregulatory framework, using distortional strain and interstitial fluid velocity as stimuli to direct the evolution of mesenchymal tissue into fibrocartilage, cartilage, or bone. Simulations were performed for three initial fracture gap sizes (0.5 mm, 1 mm, and 2 mm), under a constant axial load of 300 N. The authors used a mesh automatically generated via the Qhull algorithm, but the number and type of elements were not explicitly. The model’s predictions were qualitatively validated with published histological observations of fracture healing. Results allowed to predict that smaller gaps (0.5 mm) enhanced intramembranous ossification, while larger gaps (2 mm) was found result in endochondral ossification, characterized by a transient cartilage phase. Limitations: local mechanical stimulus and time were the only factors controlling cellular activity; angiogenesis or vascular modeling was disregarded; cartilage cells and fibroblasts were not considered; the periosteum was the only source of MSCs, although the endosteum can constitute an additional source (Lu et al. 2019); and an axisymmetric FEM model was assumed.

**Fig. 7:**
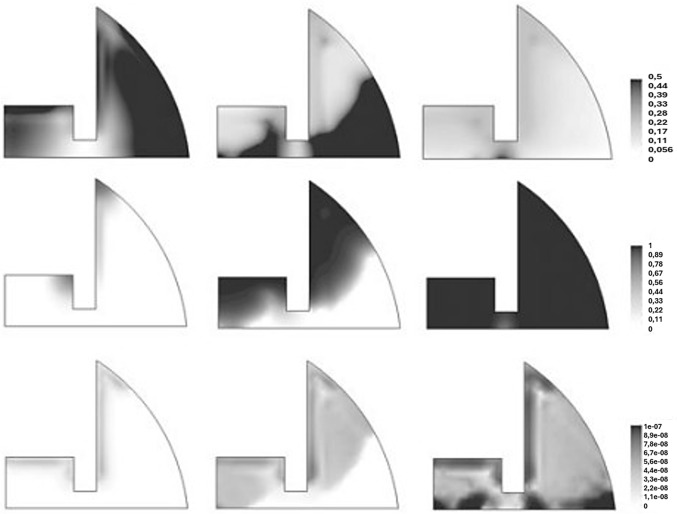
Temporal progression of cartilage, bone, and vascular tissue formation (top to bottom) at 7, 15, and 30 days after fracture (left to right), predicted by the FIC-TDG finite element simulation of biochemical bone healing. Adapted from Sapotnick and Nackenhorst (2012)

Sapotnick and Nackenhorst (2012) proposed a finite element approach combining the finite calculus method (FIC-FEM) with a time-discontinuous Galerkin (TDG) scheme to simulate the biochemical regulation of bone healing. This method was able to provide stable and accurate resolution of 12 coupled nonlinear advection–diffusion–reaction equations, modeling the dynamics of five cell populations, four extracellular matrices, and three growth factors. It was applied to a 2D axisymmetric geometry, representing a callus around a fractured long bone. Cell migration was modeled via diffusion (dependent on the matrix density) and advection (driven by growth factor gradients), with tissue production governed by reaction terms. Quadrilateral linear finite elements were used in space and TDG polynomials in time. Dirichlet boundary conditions were set on the bone interface for selected cells and growth factors; Neumann conditions were applied to the other interfaces. Fig. 7 illustrates the predicted distributions of cartilage, bone, and vascular tissue after 7, 15, and 30 days. The use of these model allowed to predicted the early healing and its cartilage formation. Besides, they found that vascularization initiates near the bone interface and progresses inward, enabling ossification. At day 30, bone tissue was predicted to occupy most of the callus region, with predicted concentrations reaching approximately $\approx$0.5 for cartilage, $\approx$1 for young bone, and $\approx 1\textrm{e}{-7}$ for vascular tissue in some regions. They were able to obtain a good agreement between their predictions and qualitatively clinical results. However, mechanical stimuli were not used, and the tissue geometry was simplified to a2D structure.

Isaksson et al. (2008) developed a coupled biological FEM model to investigate the influence of cellular activity parameters on secondary fracture healing. Their model was combines a poroelastic axisymmetric FE structure of an ovine tibia, stabilized with an external fixator. It was implemented in ABAQUS (v6.5 SIMULIA, Dassault Systemes) with a cell activity model processed via custom subroutines. Their modeling approach incorporates a 3 mm transverse fracture gap and a 1 Hz cyclic axial load of 300 N, applied to compute the cellular behavior under biophysical stimuli (e.g., strain, fluid pressure) at peak loading. The biological module was developed including four cell types (MSC, fibroblasts, chondrocytes, and osteoblasts) and three matrix types (fibrous tissue, cartilage, bone), with rules for proliferation, differentiation, migration, apoptosis, and matrix turnover. Using the Design of Experiments (DOE) methodology, 26 parameters were evaluated (such as cartilage degradation rate, osteoblast proliferation, matrix production rates, and fibroblast apoptosis) in a two-level fractional factorial design (L64), followed by a three-level design (L27), to assess their impact on healing progression. Results allowed to predict that the normal fracture healing sequence was primarily influenced by the bone matrix production rate (49%), followed by cartilage degradation. Early-stage bone formation was found almost entirely dependent on bone matrix production (76%) and osteoblast proliferation (13%). Differently, mid- and late-stage bone formation was predicted to be mainly affected by cartilage matrix production, accounting for 75% and 34% of the variation, respectively. Total healing time was found highly dependent of the cartilage degradation rate (36%), followed by cartilage matrix production and osteoblast proliferation. Higher rates of cartilage renewal were predicted to reduce the healing time by $\approx$20 days. Nonlinear responses were observed for parameters related to fibrous tissue and cartilage, indicating the existence of optimal formation levels, as excessive amounts delayed bone healing. Although no experimental validation was performed, the predicted healing times were within known experimental limits for tibial fractures in sheep (Claes et al. 1997). Furthermore, key predictions, such as the importance of cartilage degradation, were consistent with established *in vivo* observations (Colnot and Helms 2001; Ford et al. 2003), providing indirect validation. Important limitations of this computational study include simplifications of complex biological processes, as well as the reliance on limited experimental data for parameter estimation. Furthermore, the validity of the model is limited to the chosen parameter space. Interactions between factors may not be sufficiently captured.

**Fig. 8:**
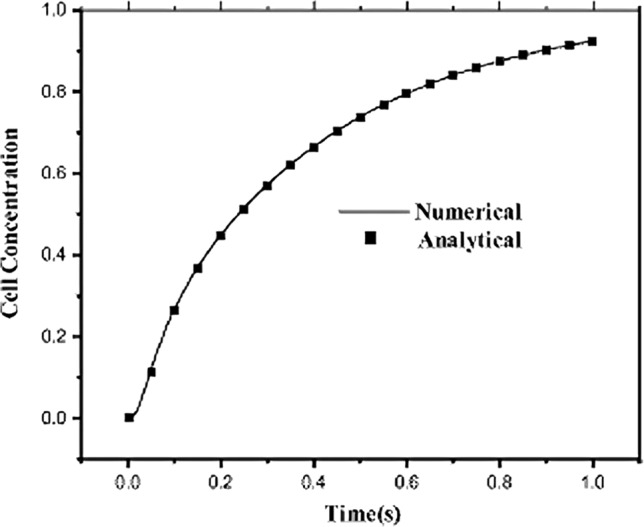
Comparison between analytical and numerical results of MSC concentration over time during bone fracture healing. Adapted from Grivas et al. (2013)

A novel meshless local boundary integral equation (LBIE) method was proposed by Grivas et al. (2013) to simulate cell proliferation during bone fracture healing, addressing limitations of mesh-based approaches in problems involving time-dependent geometries. Their model was developed considering the cellular activity as a diffusion process incorporating proliferation, differentiation, and apoptosis phenomena. A simplified 2D representation of a fractured long bone was employed, with initial MSC concentrations defined for the periosteum, bone marrow, and fracture interface. The domain was represented by 589 nodal points, and simulations were performed for predictions over 25 days post-fracture. Model validation was achieved by solving a classical diffusion problem with known analytical solution, where the numerical LBIE results showed excellent agreement with the analytical data, as illustrated in concentration–time profiles (Fig. 8). Simulation results allowed to predict progressive MSC migration from the periosteum into the callus, aligning with biological processes of intramembranous ossification and subsequent endochondral replacement. Nonetheless, this model presents some limitations, including a simplified 2D geometry, the use of MSCs without accounting for other cell types or mechanical stimuli, and the use of non-calibrated parameters.

#### Angiogenesis and vascularization

As angiogenesis is an essential phenomena to enable tissue regeneration, several relevant studies have integrated angiogenesis into mechanobiological models to evaluate the impact of mechanical conditions and fixation devices on bone healing. Ganadhiepan et al. (2021) presented a coupled 2D/3D model developed in COMSOL Multiphysics (v5.3a, COMSOL AB), combining a tri-phasic poroelastic formulation for callus tissue with angiogenesis, governed by a diffusion process modulated by local strain. Vessel density dynamically behaves as a continuous variable, influenced cell differentiation and callus stiffness oxygen-dependent production rates. Their model, validated with experimental data from sheep with 3.1 mm osteotomies (Claes et al. 1997, 2003), accurately predicted IFM, vascular density (90% in cortical/endosteal regions, 40% in periosteal by week 9), and Young’s modulus of the callus. Results allowed to predict that optimal weight-bearing increased from 30% to $\approx$100% body weight between weeks 4 and 11, depending on fracture gap size (3 mm vs 6 mm). Besides, rigid fixators (*e.g.,* half-pins) were predicted to enhance allowable loading in early stages up to +236% compared to traditional Ilizarov wire fixators. However, such model assumed cylindrical geometry, disregarded callus growth and bending loads, and focused solely on the reparative phase.

**Fig. 9:**
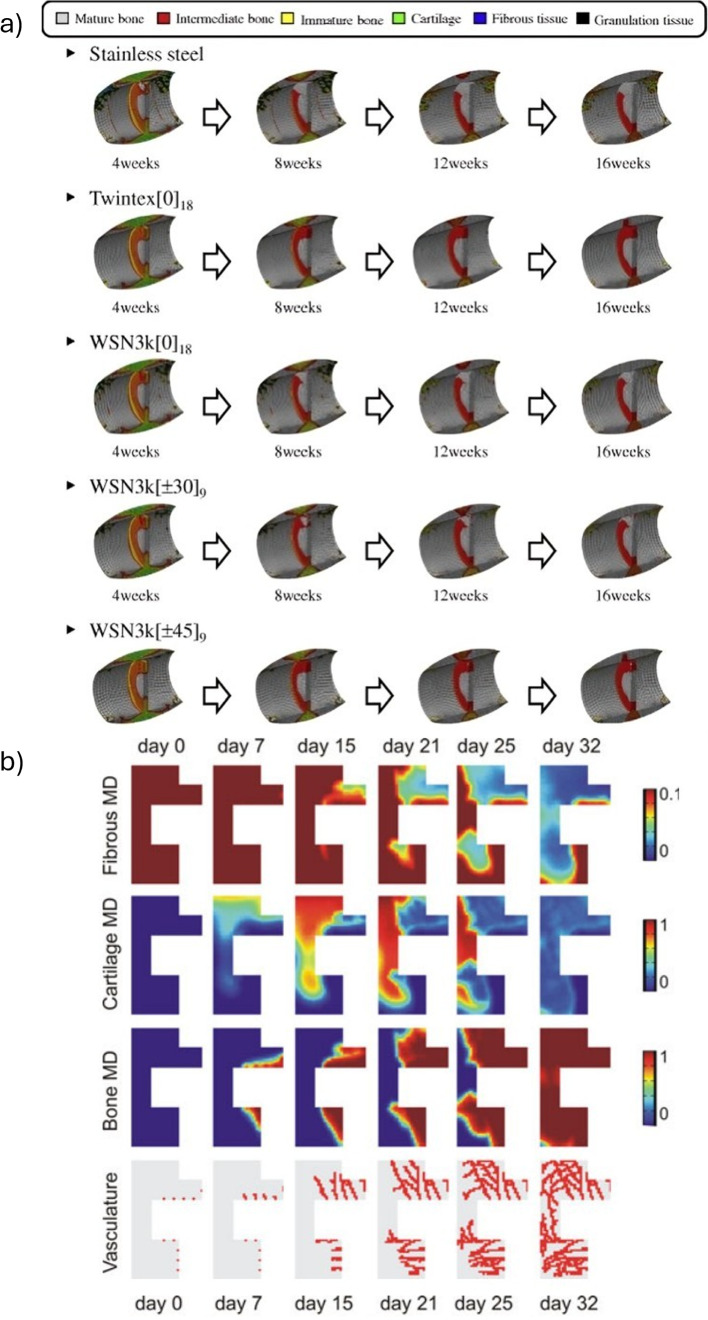
**a** Temporal evolution of tissue phenotypes within the fracture callus over 16 weeks for different bone–plate materials. Adapted from Son et al. (2014). **b** Simulated distribution of bone tissue types using the mechanistic cell model during the fracture healing. The first row illustrates the levels of fibrous tissue, while the second and third rows represent the concentrations of cartilage and bone, respectively. The last one represents the level of vascularization. Adapted from Peiffer et al. (2011)

Son et al. (2014) used ABAQUS (v6.91, Dassault Systèmes) with a Fortran user subroutine to simulate tissue differentiation via blood vessel growth through a diffusion model with a fixed coefficient ($\hbox {G} = 0.1 \hbox {mm}^{2}/\hbox {day}$). Two scenarios were analyzed: a sheep tibia stabilized with an external fixator (gap sizes: 1–2 mm) and a human tibia fixed with composite bone plates. Their model allowed to predict slower healing for 2 mm gaps, as well as higher fractions of mature bone (Young’s modulus: 4000–5500 MPa) compared to stiffer stainless steel plates using flexible bone plates (*e.g.,* Twintex $\text {[0]}_{18}$, modulus slightly above cortical bone) led to higher fractions of mature bone (Young’s modulus 4000–5500 MPa) (Fig. 9a). Vessel density was defined as a scalar proportional to the tissue modulus, but lacking spatial specificity. Validation was based on agreement with *in vivo* IFM data. Even though both models were able to capture the positive influence of angiogenesis on bone healing, their predictive ability is limited by the use of simplified geometries, as well as the lack of remodeling modeling and subject-specific parameters.

Peiffer et al. (2011) developed a hybrid computational model to investigate the role of angiogenesis in bone regeneration during fracture healing. Their model couples reaction-diffusion equations (for MSCs, fibroblasts, ECM, and growth factors) with an agent-based approach for endothelial tip cell migration. Implemented in MATLAB (MathWorks), and using a finite volume discretization on a 2D grid (25 $\mu$m resolution), time integration was performed via ROWMAP, a stiff ODE solver. The geometry was derived from histological data of a rodent femoral fracture, with a domain reduced by symmetry. Simulations reproduced key features of tissue differentiation, with blood vessel surface area increasing from 1.8% (week 1) to 41.0% (week 5). Intramembranous ossification was predicted to occur near the cortex, and endochondral ossification in the inner callus regions (Fig. 9b). The model was qualitatively validated with experimental data, showing good agreements in temporal distribution of tissues. The use of 2D geometry is a relevant limitation of such model, as well as the constant growth factor production, binary differentiation rules, and the absence of mechanical stimuli and callus growth.

O’Reilly et al. (2016) developed a 3D finite element model coupled with a lattice-based angiogenesis algorithm to investigate the impact of impaired vascularization on endochondral ossification during fracture healing. Their model, implemented in FEBio (v. 2.0, University of Utah) and COMSOL MULTIPHYSICS (v.4.3, COMSOL), used geometric and material properties of murine tibial fractures (e.g., 6.33 mm callus length, 0.17 mm fracture gap) and assumed initial granulation tissue in the callus. A lattice with $1 \times 10^6$ points/mm^3^ was used to discretize the domain, representing cell positions and vessel growth. Angiogenesis was modeled as strain-dependent directional growth of endothelial cells, governed by parameters such as vessel growth rate (0.03 mm/1/2 day), minimum oxygen tension for endochondral ossification ($\textrm{O}_2 = 50.48 \times 10^{-12}$ mol/mm^3^), and strain inhibition threshold (9%). Tissue differentiation followed oxygen- and stiffness-dependent rules: hypoxia ($\textrm{O}_2 < 30.48 \times 10^{-12}$ mol/mm^3^) was predicted to conduct to chondrogenesis, while normoxia promoted osteogenesis. Model validation was achieved by comparing predictions to histological data from murine tibiae at 10 and 20 days post-fracture, showing accurate spatial distributions of cartilage, hypertrophic cartilage, and bone. Under normal angiogenesis, the model was able to replicate the observed conversion of bone cartilage in the callus periphery by 20 days post-fracture. Under low to severe angiogenic impairments, simulations predicted persistence of cartilage, reduced hypertrophy, and fibrous tissue formation, which agrees with observations carried out in ischemic mouse models (Miedel et al. 2013). Despite the robust biological insights, limitations include the use of idealized geometry, assumptions of constant cell behavior rules, and reliance on murine data, which may limit direct clinical extrapolation.

#### Oxygen supply and biochemical signaling

As oxygen plays a pivotal role in bone regeneration, by regulating cellular differentiation and tissue formation (Lu et al. 2013), Burke and Kelly (2012) developed a coupled computational model to investigate how oxygen availability influences MSC differentiation during skeletal tissue regeneration. Their model integrated an axisymmetric FEM implemented in MSC Marc (v. 2008r1, MSC Software Corporation) with a biphasic material representation of the diaphyseal bone, incorporating cortical bone and external callus. The geometry was defined as a cylindrical bone shaft with 14 mm internal diameter, 20 mm external diameter, and 28 mm total callus diameter, spanning a 3 mm transverse fracture gap. A 300 N axial ramp load (0.5 s duration) was applied to simulate weight-bearing conditions. Tissue differentiation was defined using a dual regulatory algorithm: (i) oxygen availability, which was essential for both osteogenesis and adipogenesis but promoted chondrogenesis under hypoxic conditions; and (ii) substrate stiffness, with stiff regions favoring osteogenesis, soft regions promoting adipogenesis, and all other conditions leading to fibrogenesis. The presence of vascular supply was a prerequisite for both osteogenesis and adipogenesis. Simulations predicted that hypoxic regions supported chondrogenesis, whereas sufficiently oxygenated and stiff regions promoted osteogenesis, and oxygenated but compliant substrates favored adipogenesis. Sensitivity analysis revealed that healing strongly depends on angiogenic and mechanical parameters. Increasing the angiogenic strain threshold from 6% to 8% accelerated healing by 3 days, while reducing it to 4% delayed healing by 6 days and further reduction to 2% led to non-union. Reducing or doubling the angiogenic diffusion coefficient altered healing time by 9 days or 1 day, respectively, highlighting a minimum limit set by the bone formation rate, while increasing tissue formation rate also shortened healing up to a saturation point. Vascular boundary conditions were critical: removal of marrow or periosteal sources delayed or prevented healing, whereas adding a soft tissue source decreased healing time by 1 day. Axial loads above 700 N consistently caused non-union. These results provide preliminary support for the hypothesis that substrate stiffness and oxygen play a key role in regulating MSC fate during regenerative events such as fracture healing. Model predictions were in good agreement with previously reported experimental data on oxygen tension in the periosteal callus (Vetter et al. 2010; Epari et al. 2008), although no direct *in vivo* validation was performed in this study. Moreover, the model assumes constant oxygen consumption, uses axisymmetric geometry, and simplified representations of cell migration, angiogenesis, and marrow reestablishment. Moreover, it did not account for growth factor gradients, callus growth dynamics, or the role of substrate stiffness in regulating chondrogenesis, which may limit its predictive accuracy.

Borgiani et al. (2021) developed a 3D FEM model to simulate the early stages of bone defect healing, focusing on BMP-2-mediated MSC migration and the influence of chemotactic gradients on tissue patterning. The model geometry, implemented in ABAQUS (v. 6.12–2, Dassault Systèmes), consisted of a rat femur idealized as a hollow cylinder with a 5 mm transverse osteotomy in the midshaft. Domain included cortical bone, marrow cavity, a surrounding callus growth region, and an external fixator. The callus region was meshed with 8-node poroelastic brick elements (C3D8P) with 0.25 mm of average size of, allowing fine resolution for prediction of mechanical stimuli and tissue evolution. Boundary conditions were defined based on physiological gait data reported in the literature (Wehner et al. 2010), with loading applied at the proximal end and the distal end fixed. A second agent-based model was used to simulate the spatiotemporal dynamics of BMP-2 concentration, incorporating cell-driven production and consumption, exponential degradation (half-life = 0.42 days), Fickian diffusion ($D = 8.64 \times 10^{-2}~\text {cm}^2/\text {day}$), and gradual release from a collagen sponge (half-life = 3.25 days). Chemotactic and proliferative responses of MSCs were modulated by BMP-2 levels, with maximal chemotaxis at $1~\text {ng/cm}^3$ and peak proliferation at $200~\text {ng/cm}^3$. Simulations were able to accurately predict marrow cavity encapsulation and non-union in untreated defects, as well as periosteal bone bridging under BMP-2 treatment, highlighting BMP-2-induced chemotaxis as the key driver of progenitor cell migration and tissue organization. Model outcomes were validated using *in vivo* micro-CT data, showing some disagreements in mineralized callus volume at 2, 4, and 6 weeks post-operation. The largest inaccuracies were observed in week 2, being the bone volume underestimated by $\approx$55.6% for BMP-2 and $\approx$74.7% for BMP-2 + load. Key limitations include no modeling of angiogenesis, bone remodeling, marrow tissue formation, and BMP-2-driven enhancement of differentiation, which may contribute to the observed inaccuracies with experimental bone volumes. Nonetheless, the model illustrates the potential of multiscale *in silico* approaches to predict therapeutic mechanisms and improve regenerative strategies.

Cheng et al. (2021) investigated the relationship between early systemic immune responses and long-term bone regeneration, using a rat model of critical-sized femoral defects with delayed or early BMP-2 treatment. Immune profiling was performed longitudinally via flow cytometry and Luminex multiplex tests on blood samples, collected up to 20 weeks post-injury, while bone healing was assessed through *in vivo* micro-CT at 6 and 12 weeks, followed by *ex vivo* biomechanical testing at week 20. Computational analyses included partial least squares regression (PLSR) implemented in MATLAB (MathWorks) and nonlinear symbolic regression using Evolved Analytics DataModeler (Evolved Analytics, LLC). These models consistently predicted elevated levels of circulating myeloid-derived suppressor cells (MDSCs) and interleukin-10 (IL-10) at one week post-treatment as robust early predictors of impaired bone regeneration, achieving high predictive accuracy (ensemble model $R^2$ = 0.9255). On the other hand, increased emerging of B cells, T helper cells, and cytokines, such as IL-6 and IL-13, was positively associated with functional bone repair. Although this study did not incorporate mechanistic or FEM-based simulations, it highlights that multivariate data-driven models hold the potential to predict regenerative outcomes based on immune biomarkers. Some of the observed changes in immune cell populations may have been influenced by age-related physiological shifts, rather than being only attributed to trauma or treatment effects. They also exclude the use of mechanistic experiments to confirm whether the identified biomarkers directly drive bone regeneration outcomes. Nevertheless, results allowed to predict the importance of early immune responses in bone regeneration and support the use of systemic immune profiling as a valuable tool for predicting healing trajectories and guiding future immunotherapeutic strategies.

Bailón-Plaza and Van Der Meulen (2001) developed a 2D mathematical model to investigate the regulatory effects of generic growth factors on fracture healing. Their model simulates the interactions between mesenchymal stem cells, chondrocytes, osteoblasts, two extracellular matrices (cartilage and bone), and two diffusive growth factors (chondrogenic and osteogenic). Partial differential equations (PDEs) were defined and solved using an alternating direction implicit (ADI) finite difference method, with a mesh size of 0.02 cm and a time step of 0.24 h. Simulations were carried out for prediction of key biological timepoints, such as chondrogenic factor peaking on days 5 and 9 and reaching no expression by day 20, while osteogenic factor peaking on days 5 and 15. Such are consistent results with *in vivo* TGF-$\beta$1 expression patterns. On the one hand, quantitative predictions allowed to conclude that low osteogenic factor production (e.g., $G_{Igb} = 200$) promotes non-union scenarios, with only $\approx$10% of bone tissue found in the external callus at day 20. On the other hand, higher levels ($G_{Igb} = 500$–1000) allowed to predict restored normal ossification. This modeling approach was validated via comparison with simplified analytical solutions and outputs from functions written in MATLAB (MathWorks). Fibrous matrix, angiogenesis, and mechanobiological feedback were disregarded in the development of this models. Simplification of growth factors was also a modeling limitation, disregarding latent/active forms, enzyme interactions, and receptor-level dynamics. Mechanical effects, which are known to influence progenitor behavior and matrix deposition, were not considered.

#### Macrophage dynamics and inflammatory stage

**Fig. 10:**
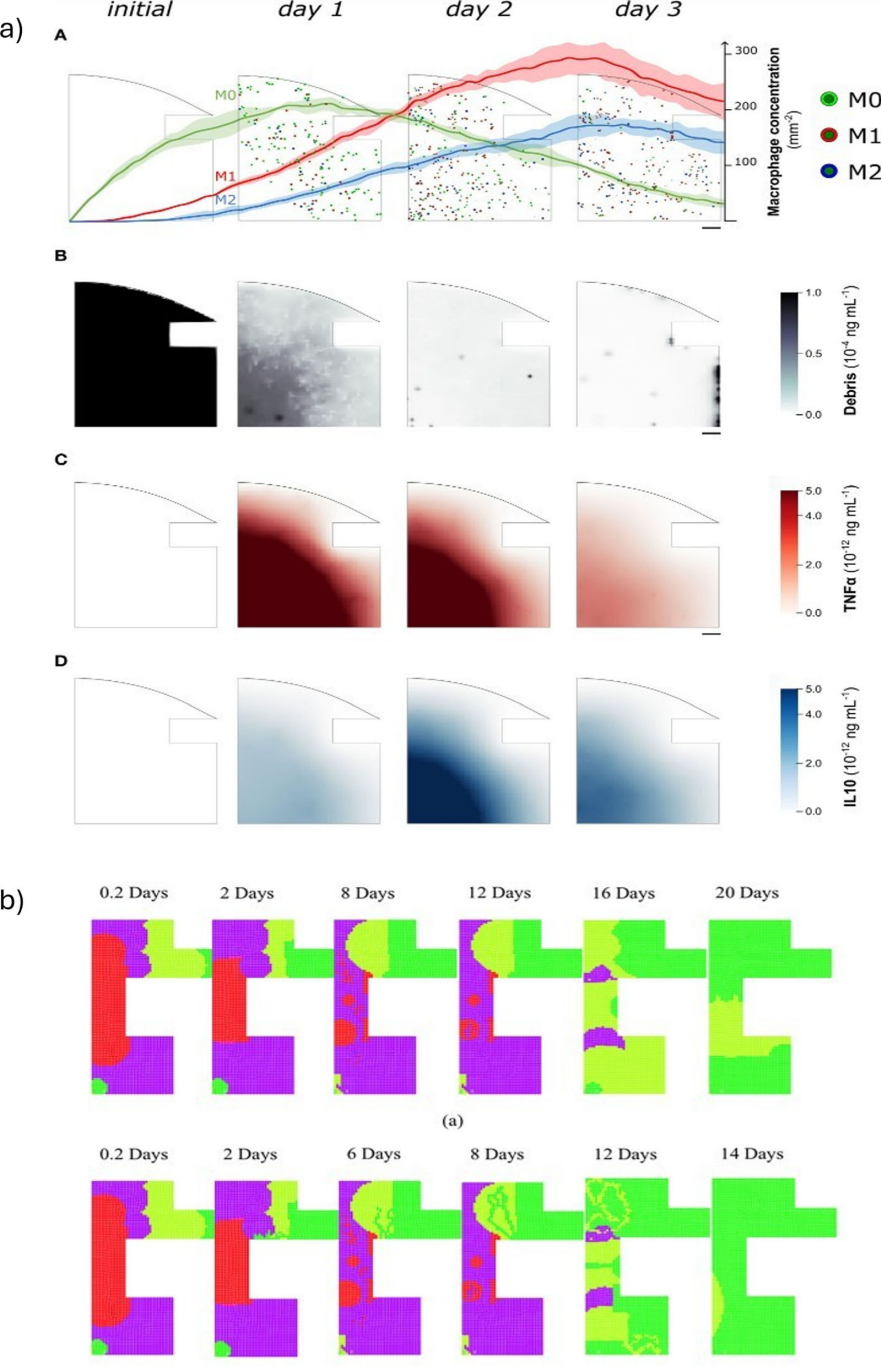
**a** Integrated visualization of simulated results with parameterization based on *in vitro* data, highlighting the ability of the COMMBINI model to capture the spatiotemporal dynamics of the inflammatory response following fracture. Panel (A) shows the evolution of the macrophage population (M0, M1, M2) over 3 days, while panels (B–D) illustrate the degradation of cellular debris and the molecular gradients of TNF-$\alpha$ and IL-10, which regulate macrophage phenotypic transitions. Adapted from Borgiani et al. (2023). **b** Predicted healing patterns: (i) without ultrasound effect; (ii) with ultrasound effect. Adapted from Grivas et al. (2019)

Borgiani et al. (2023) developed a computational model of macrophage dynamics in the bone injury immunoresponse (COMMBINI) to simulate macrophage dynamics during the early inflammatory phase of bone fracture healing. Their model was implemented in an open-source software PhysiCell (Ghaffarizadeh et al. 2018) for cell-level dynamics and BioFVM (Ghaffarizadeh et al. 2016) for cytokine diffusion, modeling a 2D transverse section of a murine tibial fracture as a cylindrical callus domain with 10 $\mu$m of spatial resolution of and temporal resolutions in the range between 1 min (cellular) and 1 s (molecular). The system allowed to predict the expression of four cell types (M0, M1, M2 macrophages, and PMNs), with phenotype transitions regulated by local cytokine concentrations (e.g., TNF-$\alpha$, IL-10) via Michaelis–Menten-type kinetics. When parameterized with values from *in vitro* studies, their model was able to predicted a macrophage density of 346.4 ± 9.3 $\hbox {mm}^{-2}$ at day 1, increasing on average by 12.7% by day 3. M0, M1, and M2 subpopulations were found to follow distinct time-dependent expressions, with M0 decreasing by 84.4%, and M1 and M2 increasing by 2.2 and 3.2 fold, respectively. These spatiotemporal dynamics are illustrated in Fig. 10a, along with the progressive clearance of cellular debris and the emergence of TNF-$\alpha$ and IL-10 driving macrophage polarization. Model calibration was subsequently performed using *in vivo* immunofluorescence imaging of macrophage subtypes (CD68$^+$/CD80$^+$ for M1, CD68$^+$/CD206$^+$ for M2) in murine tibial defects harvested at day 3 post-fracture. A novel *in silico* immunofluorescence pipeline was developed to allow good agreements between simulated distributions and experimental data. Calibration of key parameters (e.g., M1 proliferation rate, M0 recruitment rate) via a genetic algorithm decreased the mean error of the total macrophage density from 240.9 $\hbox {mm}^{-2}$ to 107.1 $\hbox {mm}^{-2}$, yielding a calibrated prediction of 457.6 ± 51.5 $\hbox {mm}^{-2}$, close to the observed 518.8 ± 8.3 $\hbox {mm}^{-2}$. Model validation on an independent femoral fracture dataset (0.7 mm gap) showed strong agreements between simulated and experimental macrophage densities at day 3. Despite these promising results, some limitations were not solved, namely the use of simplified 2D geometries, lack of mechanical stimuli, and no modeling of adaptive immune components.

Naveiro et al. (2021) developed a FEM model to simulate the initial phase of callus formation during long bone fracture healing, combining a diffusion-based formulation with a mesh-growing algorithm that enabled natural, geometry-driven expansion of the callus without relying on a predefined mesh. Their modeling approach was implemented using a custom FORTRAN solver for the diffusion equations and a Python 3.8 script to manage the overall simulation workflow. A 2D axisymmetric domain was discretized with quadratic triangular elements, with 1 mm of average mesh size. MSC and chondrocyte concentrations were computed via Fick’s law of diffusion, while the local concentrations of TNF-$\alpha$ and BMP-2 were used to modeled the growth velocity through a sigmoidal parametric function. The callus growth algorithm was developed to iteratively add new elements to the boundary once threshold concentrations are reached, such that the mesh is dynamically updated. Simulations were performed using three femoral fracture types (transverse, oblique, and comminuted), all treated with intramedullary rod, ensuring complete callus formation in each case, even if geometrically complex scenarios were considered. Results allowed to quantitatively predict 256 $\hbox {mm}^2$of callus area after 10 days in the transverse fracture case (4 mm gap) whose error is < 7% deviation from previously reported *in vivo* data. While the model offers a novel framework for simulating early-stage callus morphogenesis, it was limited to the initial phase of healing, disregarding mechanical loading and tissue differentiation, and presenting results reduced to 2D axisymmetric geometries.

Baratchart et al. (2022) developed a system of coupled ordinary differential equations (ODEs) to model the temporal dynamics of seven cellular populations (osteoblasts, osteoclasts, naive monocytes, pro-inflammatory monocytes, naive macrophages, pro- and anti-inflammatory macrophages), as well as the bone volume during the repair of non-critical bone injuries. Their model was designed incorporating experimentally derived longitudinal data from murine tibial injury experiments, with measurements collected at days 0, 1, 2, 3, 7, and 14 post-injury. Additional 18 model structures were developed based on different combinations of biologically plausible hypotheses about cell–cell interactions. Each variant was evaluated using both the Akaike information criterion (AIC), which quantifies model quality by balancing goodness of fit and model complexity, and the number of residuals with error <1. A close alignment between simulated and observed data was obtained, the best-performing model assumed that: (i) osteoblasts promote osteoclast differentiation; (ii) injury signals drive osteoblast expansion; and (iii) anti-inflammatory macrophages suppress pro-inflammatory macrophages. This model achieved the lowest AIC value (AIC = 39), indicating the best-fit model with the least complexity, and with 25 out of 40 residuals <1, highlighting a high degree of quantitative concordance with the experimental data. Model validation was further performed by simulating the biological impact of oncostatin M (OSM) depletion (a cytokine secreted by anti-inflammatory macrophages that promotes osteoblast proliferation and mineralization while inhibiting osteoclast activity). Parameters governing osteoblast expansion and mineralization were reduced by 50%, and osteoclast inhibition reduced by 80% to emulate the physiological consequences of OSM deficiency. Simulations allowed to accurately predict the expression of osteoblasts and osteoclasts, as well as bone volume dynamics, according to data experimentally observed. Nevertheless, the model was developed disregarding spatial dynamics, and other immune or stromal cell types (e.g., T cells or MSCs). Besides, its dependency on time-resolved *in vivo* data may limit generalization beyond the specific experimental context.

#### Impact of mechanical stimulation

Grivas et al. (2019) developed a computational mechanobioregulatory model to simulate bone fracture healing under low-intensity pulsed ultrasound stimulation (LIPUS). The proposed model integrates MSC migration and differentiation, angiogenesis, and oxygen concentration with mechanical stimuli derived from FEM simulations. These mechanical inputs regulate tissue differentiation according to the Prendergast and Checa mechanoregulation framework (Checa and Prendergast 2009). The finite element analysis was performed using ANSYS (v. 16.2, ANSYS Inc.), applied to a 2D representation of a standardized rat femoral fracture model. Results allowed to predict that LIPUS enhanced both intramembranous and endochondral ossification, leading to a reduction in total healing time by up to 30%, depending on the assumed origin of progenitor cells (Fig. 10b). The lack of experimental data for model calibration limited the impact of this model. Additionally, the ultrasound effect was represented in a simplified, pressure field imposed by US, without explicitly modeling wave-specific parameters such as frequency or pulse duration. Nevertheless, the model presents an innovative approach by iteratively coupling mechanical and biological factors and highlights the potential of LIPUS to accelerate vascularization and ossification during bone healing.

Vavva et al. (2018) Vavva et al. (2018) developed a bioregulatory computational model to investigate the influence of low-intensity ultrasound on bone fracture healing, with a specific emphasis on angiogenesis and vascular endothelial growth factor (VEGF) transport. The hybrid model combines a tissue-level system of 11 partial differential equations, based on the work of Peiffer et al. (2011), describing the evolution of multiple cell types, growth factors, extracellular matrices, and nutrient concentrations, with a discrete vascular network model representing endothelial tip cell dynamics. The influence of ultrasound was incorporated via modifications to the VEGF transport equation, accounting for interstitial fluid velocity induced by acoustic pressure, and by applying boundary conditions corresponding to different ultrasound intensities (15–75 $\mathrm {mW/cm^2}$). Simulations were performed considering a2D callus geometry extracted from real rodent fracture data and using the method of lines (MOL), finite volume spatial discretization, and ROWMAP for time integration. Quantitative comparisons with experimental data revealed that ultrasound accelerates angiogenesis (first vessels appear at day 3 post-fracture vs day 7 in control) and enhances ossification, resulting in higher bone matrix densities at day 25 (e.g., 93.75% with ultrasound vs 85.42% without in periosteal callus). The optimal acoustic intensity was predicted to be 50 $\mathrm {mW/cm^2}$. Model limitations include 2D geometry simplification, lack of mechanical stimuli integration, and overestimation of cartilage density. Nonetheless, this study demonstrates that ultrasound-driven VEGF enhancement can significantly improve vascularization and bone formation.

#### Summary

A summary of model types, biophysical inputs, model characteristics, main predictions, validation method, and limitations related to each study focused on the biological modeling approaches is presented in Table 2.

**Table 2:** Overview of biological models developed to simulate cellular interactions and biological mechanisms during bone healing, including immune response and cellular regulation in the repair process

Model type	Biophysical inputs	Model characteristics	Main predictions	Validation method	Limitations	Reference
FEM + CLSVOF (interface-capturing)	500 N axial load; hydrostatic and distortional strain thresholds; biological constraints (vascularization, cartilage age); tissue growth velocity	Axisymmetric geometry of ovine metatarsus with external fixator; materials: soft tissue, cartilage, woven and lamellar bone, marrow, muscle; OpenFOAM + custom FEM solver (CSparse)	Predicts tissue front propagation; captures both endochondral and intramembranous ossification patterns	Comparison with *in vivo* data; sensitivity study with ±10% variation in growth velocity	Requires predefined tissue boundaries; simplified vascularization	Pietsch et al. (2018)
2D axisymmetric FE model	Axial load (300 N); fracture gap sizes (0.5, 1, 2 mm); Prendergast’s mechanoregulatory stimuli (strain + fluid velocity)	ABAQUS + MATLAB routine; Cylindrical mid-diaphyseal fracture with external fixator; automatic Qhull-based mesh	Smaller gaps promote intramembranous ossification; larger gaps induced endochondral ossification with cartilage intermediate; healing mode transition observed between 1 and 2 mm	Qualitative comparison with histological data from the literature	No angiogenesis or vascular modeling; limited MSC sources; cartilage and fibroblasts neglected; axisymmetric simplification	Gómez-Benito et al. (2005)
FEM (FIC-FEM + TDG)	Growth factor gradients, extracellular matrix densities, diffusion/advection/reaction parameters	2D axisymmetric finite element model; 12 coupled nonlinear PDEs (5 cell types, 4 matrices, 3 growth factors); cell migration via matrix-dependent diffusion and chemotactic advection; TDG temporal discretization	Cartilage dominates early healing; bone formation follows vascular ingrowth; at day 30, bone $\approx$1, cartilage $\approx$0.5, vascular tissue $\approx$ $10^{-7}$	Qualitative comparison to biological expectations and consistency with model behavior	No mechanical stimulus; no experimental validation; simplified 2D geometry	Sapotnick and Nackenhorst (2012)
FEM	MSC, fibroblasts, chondrocytes, osteoblasts; fibrous tissue, cartilage, bone; strain, fluid pressure; cyclic load 300 N at 1 Hz	Axisymmetric FE model of ovine tibia with 3 mm fracture gap; ABAQUS + custom subroutines; DOE on 26 parameters (L64, L27)	Bone matrix production (49%) and cartilage degradation (36%) most influential; early bone formation driven by bone matrix production (76%) and osteoblast proliferation (13%); mid/late bone formation influenced by cartilage matrix production (75%, 34%); nonlinear effects observed; cartilage renewal reduces healing time by $\approx$20 days	Indirect validation via predicted healing times consistent with experimental data; biological trends confirmed	Simplified biology; limited data; parameter space limited	Isaksson et al. (2008)
Meshless diffusion-based model (LBIE)	MSC diffusion, proliferation, differentiation, apoptosis	2D simplified long bone geometry; meshless LBIE method with 589 nodes; simulated 25 days of healing	Captured MSC migration patterns consistent with intramembranous and endochondral ossification stages; demonstrated high numerical accuracy without need for remeshing	Numerical comparison with analytical solution for classical diffusion problem	2D simplification; no mechanical stimuli; non-calibrated parameters	Grivas et al. (2013)
FEM	Gap size (3/6 mm); ICF; MSC and angiogenesis	2D and 3D axisymmetric models of sheep metatarsal fracture under ICF; COMSOL poroelastic and mechanobiological simulation with time-dependent solver; triangular/tetrahedral mesh; iterative angiogenesis and callus stiffening loop	Optimal weight-bearing ranges depend on gap size, ICF rigidity and BW; early high loading inhibits angiogenesis; rigid ICFs allow earlier loading; angiogenesis correlates with callus stiffening	Comparison with experimental data: IFM vs time, callus stiffness, and vessel density at 9 weeks	Simplifies fracture geometry and loading, ignores callus growth, and uses generalized parameters	Ganadhiepan et al. (2021)
FEM	Composite bone plates + blood vessel growth	3D FE model of fractured human tibia with composite plate; ABAQUS v6.9.1; C3D8T elements; mechanoregulation and diffusion equations via Fortran user subroutines; blood vessel growth modulates callus stiffness; simulation over 112 days	Flexible Twintex$\text {[0]}_{18}$ plates promoted more mature bone (4000–5500 MPa) than stainless steel; vascular growth enhanced healing and reduced sensitivity to implant stiffness	Comparison with *in vivo* sheep data (gap size and IFM trends); tissue phenotype distributions vs histological data	Simplified geometry; constant load conditions; uniform vascular density; no patient variability	Son et al. (2014)
Hybrid (finite volumes + discrete cell tracking)	Growth factors (chondrogenic, osteogenic, VEGF), MSC and fibroblast source densities, matrix fiber orientation	2D callus domain (1/4 symmetry); Finite Volume Method discretization with $\Delta x = 25\ \mu$m; ROWMAP time integration; MATLAB implementation; angiogenesis via tip cell tracking; simulates MSC/chondrocyte/osteoblast differentiation	Predicts spatiotemporal patterns of vascularization and tissue formation; blood vessel surface fraction rises from 1.8% (week 1) to 41.0% (week 5); VEGF treatments enhance healing	Comparison with experimental tissue fraction data; alignment in periosteal, intercortical and endosteal regions	Simplified 2D geometry; fixed domain; binary differentiation rules	Peiffer et al. (2011)
3D FEM + lattice-based angiogenesis	Strain, oxygen tension, tissue stiffness	Coupled FEBio + COMSOL simulations of murine tibial fracture; 6.33 mm callus, 0.17 mm gap; $1 \times 10^6$ points/mm^3^; vessel growth rate 0.03 mm/1/2 day; chondrogenesis if $O_2 < 30.48 \times 10^{-12}$ mol/mm^3^; inhibition threshold 9% strain	Accurate prediction of ossification zones under normal conditions; vascular impairment delays cartilage hypertrophy and increases fibrous tissue	Validation against histological data at 10 and 20 days post-fracture from murine tibiae; consistent with *in vivo* ischemic studies	Idealized geometry; constant cell behavior rules; murine data may limit clinical extrapolation	O’Reilly et al. (2016)
FEM	Oxygen concentration, octahedral strain, fluid flow; Axial load (300 N)	Axisymmetric FE model of cylindrical bone (MSC Marc 2008r1); biphasic materials (cortical bone + callus); oxygen modeled via reaction-diffusion equation	Low oxygen (<5%) promotes chondrogenesis; high oxygen promotes osteogenesis; spatial patterns consistent with *in vivo* observations	Qualitative validation: simulation results consistent with known experimental differentiation patterns	Simplified geometry; constant oxygen consumption; ignores growth factor gradients and marrow heterogeneity	Burke and Kelly (2012)
FE + agent-based	BMP-2 diffusion, production, degradation; mechanical loading (gait)	3D hollow cylinder (rat femur) with 5 mm osteotomy; Abaqus 6.12–2; C3D8P elements (0.25 mm in callus); BMP-2 model includes Fickian diffusion ($D = 8.64 \times 10^{-2}$ $\hbox {cm}^2$/day), exponential degradation (half-life 0.42 d), sponge release (half-life 3.25 d); max chemotaxis at 1 ng/$\hbox {cm}^3$, proliferation at 200 ng/$\hbox {cm}^3$	Chemotaxis-driven MSC migration directs spatial tissue patterning and periosteal bridging; predicts non-union in untreated defects	Comparison with *in vivo* $\mu$CT; good match in spatial patterns, discrepancies in BV (74.7% at week 2 for BMP-2 + load)	No angiogenesis, bone remodeling, marrow tissue formation and does not model BMP-2-induced enhancement of differentiation	Borgiani et al. (2021)
Data-driven multivariate regression (linear and nonlinear)	Circulating immune cells (e.g., MDSCs, B cells, T cells); cytokines (e.g., IL-10, IL-6, IL-13, IP-10)	PLSR implemented in MATLAB; nonlinear symbolic regression via Evolved Analytics DataModeler; based on longitudinal blood data (flow cytometry, Luminex)	Elevated MDSCs and IL-10 at 1 week post-treatment predict impaired bone healing ($R^2 = 0.9255$); B cells, T helper cells, IL-6/IL-13 positively correlated with regeneration	*In vivo* $\mu$CT at weeks 6 and 12; *ex vivo* biomechanical testing at week 20	No mechanistic modeling; absence of causal validation; immune shifts may partly reflect aging effects	Cheng et al. (2021)
Analytical	Generic chondrogenic and osteogenic growth factor diffusion, MSCs, chondrocytes, osteoblasts, ECM formation (bone, cartilage)	2D domain; ADI finite difference method; mesh size 0.02 cm; time step 0.24 h; implemented and cross-checked with MATLAB toolbox	Simulates temporal-spatial evolution of tissue differentiation; ossification fails for low osteogenic factor production ($G_{Igb}=200$), but restored for $G_{Igb}=500$–1000; replicates TGF-$\beta$1 expression timing	Compared against reduced analytical solutions and MATLAB ODE toolbox outputs	Omits fibrous tissue, angiogenesis, mechanical effects, and detailed growth factor biochemistry	Bailón-Plaza and Van Der Meulen (2001)
Agent-based model (discrete, multiscale)	Cytokine concentrations (e.g., TNF-$\alpha$, IL-10), cellular debris, macrophage recruitment and proliferation parameters	2D agent-based simulation of macrophage dynamics during early fracture healing; coupled with PDE-based cytokine diffusion; 36 parameters; implemented in PhysiCell and BioFVM	Model reproduces spatial and temporal macrophage polarization driven by cytokine gradients; calibration improved quantitative match with experimental macrophage densities	Quantitative comparison with *in vivo* immunofluorescence (day 3 post-fracture), including independent dataset for external validation	2D geometry, no mechanical loading, absence of adaptive immune cells	Borgiani et al. (2023)
FEM	MSC and chondrocyte concentrations; TNF-$\alpha$ and BMP-2 gradients	2D axisymmetric FE model with quadratic triangular elements (1 mm average size); diffusion via Fick’s law; mesh growth via parametric sigmoid velocity; FORTRAN and Python 3.8 implementation	Model reproduces natural callus morphogenesis across three fracture types; predicted callus area (transverse case) of 256 $\hbox {mm}^2$ after 10 days	Comparison with previously reported *in vivo* data; <7% deviation in callus area	Limited to initial phase; no mechanical loading, tissue differentiation, or 3D geometries	Naveiro et al. (2021)
Mathematical (ODE-based)	Longitudinal *in vivo* data from murine tibial injury: cell counts (osteoblasts, osteoclasts, monocytes/macrophages) and bone volume over time	Coupled ODE system modeling 7 cell populations + bone volume (8 variables total); 18 hypothesis-driven model variants evaluated using AIC and residuals; implemented in Python	Best-performing configuration (a3-b2-c2): Osteoblasts promote osteoclast differentiation; anti-inflammatory macrophages suppress pro-inflammatory macrophages; model reproduces key bone healing dynamics and reveals regulatory roles of immune cells	External validation via simulation of OSM cytokine depletion; parameter adjustments (-50% osteoblast expansion and mineralization, -80% osteoclast inhibition) reproduced trends from independent OSM deficient dataset	No spatial resolution; excludes other relevant cell types (e.g., T cells, MSCs); dependent on high-resolution longitudinal *in vivo* data	Baratchart et al. (2022)
FEM	LIPUS (modeled via angiogenesis enhancement)	2D FEM of rat femoral fracture; rule-based tissue differentiation based on local stimuli	LIPUS accelerates healing up to 30%; improves vascularization and ossification; healing time varies with progenitor cell origin	No validation performed	No experimental validation; ultrasound modeled simplistically without frequency/pulse parameters	Grivas et al. (2019)
Hybrid PDE + discrete vasculature model	Ultrasound intensity (15–75 $\mathrm {mW/cm^2}$), VEGF transport, acoustic pressure, interstitial fluid velocity	2D model combining 11 PDEs (cells, matrices, growth factors, oxygen) with vascular tree; ultrasound modifies VEGF via pressure-driven convection; MOL with finite volume discretization and ROWMAP solver	Accelerated angiogenesis and ossification (e.g., first vessels at day 3 vs day 7; bone matrix in periosteal callus at day 25: 93.75% vs 85.42%); optimal intensity: 50 $\mathrm {mW/cm^2}$	Comparison of tissue fractions and bone matrix with experimental data	Simplified 2D geometry; no mechanical loading; cartilage overestimation; limited to VEGF effects	Vavva et al. (2018)

### Computational models integrating biological and mechanical phenomena

#### Mechanical stability in tissue regeneration

**Fig. 11:**
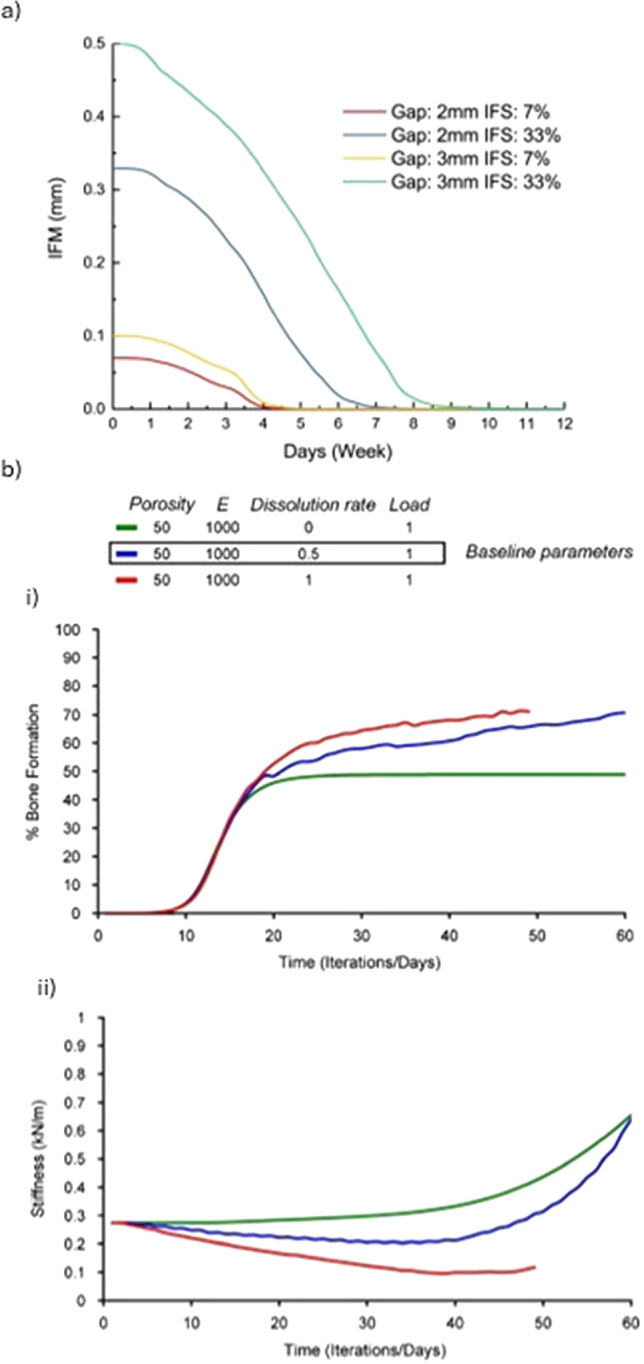
**a** Results of IFM over time for stable and unstable conditions. Adapted from Wang and Yang (2019). **b** Effect of dissolution rate on (i) bone formation (%) and (ii) mechanical stiffness of the bone-scaffold system (kN/m). Adapted from Byrne et al. (2007)

Wang and Yang (2019) developed a computational model to investigate the influence of mechanical stability on the spatial and temporal dynamics of growth factors during bone fracture healing. The model combined a finite element analysis (FEA) in ABAQUS (v. 6.13-3, Dassault Systèmes) with a fuzzy logic-based tissue differentiation algorithm implemented in MATLAB (v. 6.13-3, MathWorks), allowing predictions obtained from a co-simulation framework. An axisymmetric representation of a sheep tibial fracture with two fracture gap sizes (2 mm for stable fixation; 3 mm for unstable fixation) was used, each tested under low (7%) and high (33%) IFS conditions. Mechanical stimuli, namely dilatational and distortional strain, were computed via FEA and used, along with local tissue concentrations, as inputs to a fuzzy logic system that governed the differentiation between connective tissue, fibrocartilage, and woven bone. Partial differential equations modeled the diffusion, production, and decay of chondrogenic and osteogenic growth factors (BMP-2/4 and TGF-$\beta$1 analogs, respectively). Simulations have allowed to predict that under stable conditions, osteogenic growth factors reached the fracture gap by $\approx$4 weeks, and healing concluded within the same timeframe. Differently, under unstable conditions, predictions showed that osteogenic factor expression was delayed to 8 and 10 weeks, with overall healing taking 8–9 weeks. Higher IFSs were associated with greater deformation and wider, but slower, diffusion of growth factors. This model predicted that low mechanical stability impairs the timely delivery of osteogenic signals to the fracture site, delaying ossification (Fig. 11a). However, experimental validation was disregarded, only parameters from mixed species were used, and the biological feedback from growth factors on tissue behavior was not considered.

Avval et al. (2016) investigated the long-term response of femoral bone density to the combined presence of a hip implant and a bone fracture plate (lateral or anterior) using a mechanobiochemical bone remodeling model. Simulations were performed using ANSYS (v. 14.5, Ansys, Inc), with high-resolution FEM models based on CT-derived femur geometries, meshed with quadratic tetrahedral elements. The bone remodeling model incorporated five Michaelis–Menten enzyme reactions to express bone resorption and formation processes. These biochemical reactions were coupled with mechanical loading through a modified law of mass action, where the reaction rates were influenced by the first invariant of the strain rate tensor, $\varepsilon ^{(1)}$, representing volumetric deformation under physiological loading. Muscle and joint contact forces, representing 45% of the gait cycle (the peak load phase), were applied to simulate *in vivo* conditions. Bone density was iteratively updated based on the concentrations of newly formed and reabsorbed bone until convergence was reached. This model allowed to predict severe bone loss directly beneath the plates (up to $\approx$70%) and localized bone formation near the proximal and distal screw holes (up to $\approx$110%), due to stress concentration. To validate the model, a separate simulation was performed mimicking the clinical conditions of an intact femur with a lateral plate, even though without hip implant. Simulations were performed such that bone density changes could be evaluated in four quadrants (posterior, medial, lateral, anterior) within a specific femoral region defined in the clinical study. The predicted bone losses in the posterior (32%), lateral (27%), and anterior (15%) quadrants were found within the ranges clinically reported (45% to 6% posterior, 28% to 5% lateral, and 37% to 1% anterior), demonstrating good agreements between the simulation data and experimental data. Direct validation for the dual-implant condition (plate + hip stem) was not carried out due to the lack of available clinical data. The assumption of isotropic material properties was considered; besides, only a simplified modeling of cellular mechanotransduction processes was developed. Finally, a realistic correspondence between model iterations and actual biological time was not carried out.

#### Dynamic loading and cellular behavior

Dynamic loading, which mainly includes cyclic or intermittent mechanical pressures, has a relevant role in bone regeneration because it stimulates cellular activity and improves tissue organization (Ma et al. 2023; Ghimire et al. 2018). Unlike static loading, dynamic stimuli increase osteogenesis via mechanotransduction pathways, which improves the healing environment (Ma et al. 2023).

García-Aznar et al. (2007) proposed a mechanobiological FEM model to investigate the influence of interfragmentary movement on the callus growth during bone fracture healing. The model accounted for the proliferation, differentiation, and hypertrophy of MSCs, osteoblasts, fibroblasts, and chondrocytes, regulated by the local mechanical environment expressed through the second deviatoric strain invariant. A 2D axisymmetric geometry representing a 17 mm diameter long bone with a mid-diaphyseal fracture and a 2 mm fracture gap, stabilized by an external fixator of variable stiffness, was implemented in ABAQUS ( v6.3, Dassault Systèmes) with user-defined subroutines. Simulations under different axial loads (250, 500, and 750 N) predicted temporal changes in callus morphology: during the first 6 weeks, callus size increased significantly, with radial thickness reaching values comparable to cortical bone, followed by a stabilization phase. The model predicted interfragmentary motions ranging from $\approx$0.2 mm (350 N, week 6) up to $\approx$1.5 mm (750 N, week 2), closely matching experimental data in sheep (Claes and Heigele 1999). Healing was completed within 8 weeks for all loading cases, although delayed under 750 N, where osteoblasts filled the gap only after week 8. Quantitative predictions further indicated that excessive motion (>2 mm) led to persistent fibrous tissue formation and impaired ossification, whereas moderate loading enhanced endochondral progression. While the model successfully captures key mechanobiological aspects of periosteal callus growth, it remains limited by assumptions regarding growth mechanisms, cell saturation, matrix production, simplified geometry, and the exclusion of endosteal callus formation.

Ghimire et al. (2018) investigated the impact of dynamic mechanical loading on solute transport and tissue differentiation during the early stages of bone fracture healing. To this purpose, they developed a poroelastic FEM model incorporating mechanical stimuli-mediated MSC differentiation, tissue formation, and convective transport of cells and growth factors. Their model was implemented in COMSOL MULTIPHYSICS (v. 5.2, COMSOL) using a 2D axisymmetric geometry of a rodent femur, comprising 1933 triangular elements with 20,745 degrees of freedom. A physiologically relevant loading protocol was applied: cyclic axial compression at 5–15% strain and 0.01–10 Hz for six hours. Simulation results allowed to predict that, compared to free diffusion, dynamic loading increases MSC concentration in the endosteal zone by up to 20% during the first hour and enhanced chondrogenic growth factor uptake by 60% in the periosteal and 100% in the cortical callus. Their simulations also allowed to find that dynamic loading at 1 Hz (10% strain magnitude) result in 15% increase in cartilage content and simultaneous reduction of fibrous tissue by 5%, promoting endochondral ossification. Besides, they predicted that higher strain (15%) can induce excessive fibrous tissue formation, while low strain (5%) or low frequency (0.01 Hz) can suppress cartilage development. Model validation was performed with experimental data, by comparing tissue composition at day 7 across three zones of the callus. The model accurately predicted higher fibrous tissue fractions in the endosteal (90%), cortical (93%), and periosteal zones (65%), cartilage localized primarily in the periosteal zone (up to 15%), and bone in the periosteal (20%), according with histological findings. Moreover, simulated bone and cartilage contents ($\approx$16% and $\approx$8.3%, respectively) were consistent with another study in murine tibial fractures (Harrison et al. 2003). Despite these promising results, only simplified 2D geometry were used; moreover, the focus on the first week of healing is a prediction limitation; the assumption of constant mechanical properties in the callus also limits its clinical translation.

Irandoust and Müftü (2023) investigated how different mechanical loading profiles affect fluid flow and bone healing around dental implants. A 2D axisymmetric FEM model of a screw-shaped titanium implant and surrounding bone tissue was developed using ANSYS (Mechanical APDL, ANSYS), where the healing gap was modeled as a saturated porous medium governed by Biot’s poroelasticity theory. Displacement-controlled cyclic loading was applied via ramp and haversine functions with 10 $\mu$m of peak displacement and 1 Hz of frequency. Bone healing was simulated over 30 days using a mechanobiological algorithm that incorporated MSC diffusion and a mechanoregulatory stimulus function, based on octahedral shear strain and interstitial fluid velocity. Results allowed to predict that fluid velocity in the range 0.1 and 12 $\mu$m/s can be found under the implant on day 1, and decreased over time; a different dynamics was found in more coronal regions , where the velocity remain below 10 $\mu$m/s. Fluid velocity in the coronal region and at the interface between the lateral implant surface and the surrounding trabecular bone required more time to reach steady state compared to the region beneath the implant. Predictions based on the average healing stimulus revealed the formation of mature trabecular bone, whereas using the maximum stimulus resulted in persistent fibrous tissue. Limitations include the use of linear elastic material behavior, 2D axisymmetric geometry; besides, the directional influences of fluid flow on stem cell migration were also disregarded.

Byrne et al. (2007) developed a 3D computational model to predict tissue differentiation and bone regeneration within a scaffold as a function of porosity, Young’s modulus, and dissolution rate, under low and high mechanical loading conditions. Their model integrates a biphasic poroelastic finite element formulation (solved in ABAQUS (v. 6.5-1, Dassault Systèmes)) with a mechanobiological algorithm that governs MSC dynamics based on local shear strain and fluid velocity. Cell proliferation and migration were simulated using a 3D random walk approach on a lattice defined within each element. Scaffold porosity (30–70%), stiffness (750–1250 MPa), loading (1–2 MPa), and dissolution rate (0–1% per iteration) were systematically varied in 54 simulations. Under baseline conditions (50% porosity, 1000 MPa, 0.5%/iteration), the model allowed to predict 71% bone regeneration after 21 iterations, with increased porosity enhancing bone and cartilage formation, but reducing mechanical stiffness. High dissolution rates were found to trigger premature scaffold failure (stiffness <0.1 kN/m), while medium dissolution rates were found to optimize both regeneration and mechanical integrity (Fig. 11b). Increased Young’s modulus was predicted to improve bone formation (2%),and critical behaviors were found for high load. Although not experimentally validated, the model demonstrates how scaffold design can be optimized for site-specific conditions. Limitations include the assumption of uniform nutrient supply, no modeling of the apoptosis phenomenon, no validation, and the use of simplified linear degradation kinetics.

**Fig. 12:**
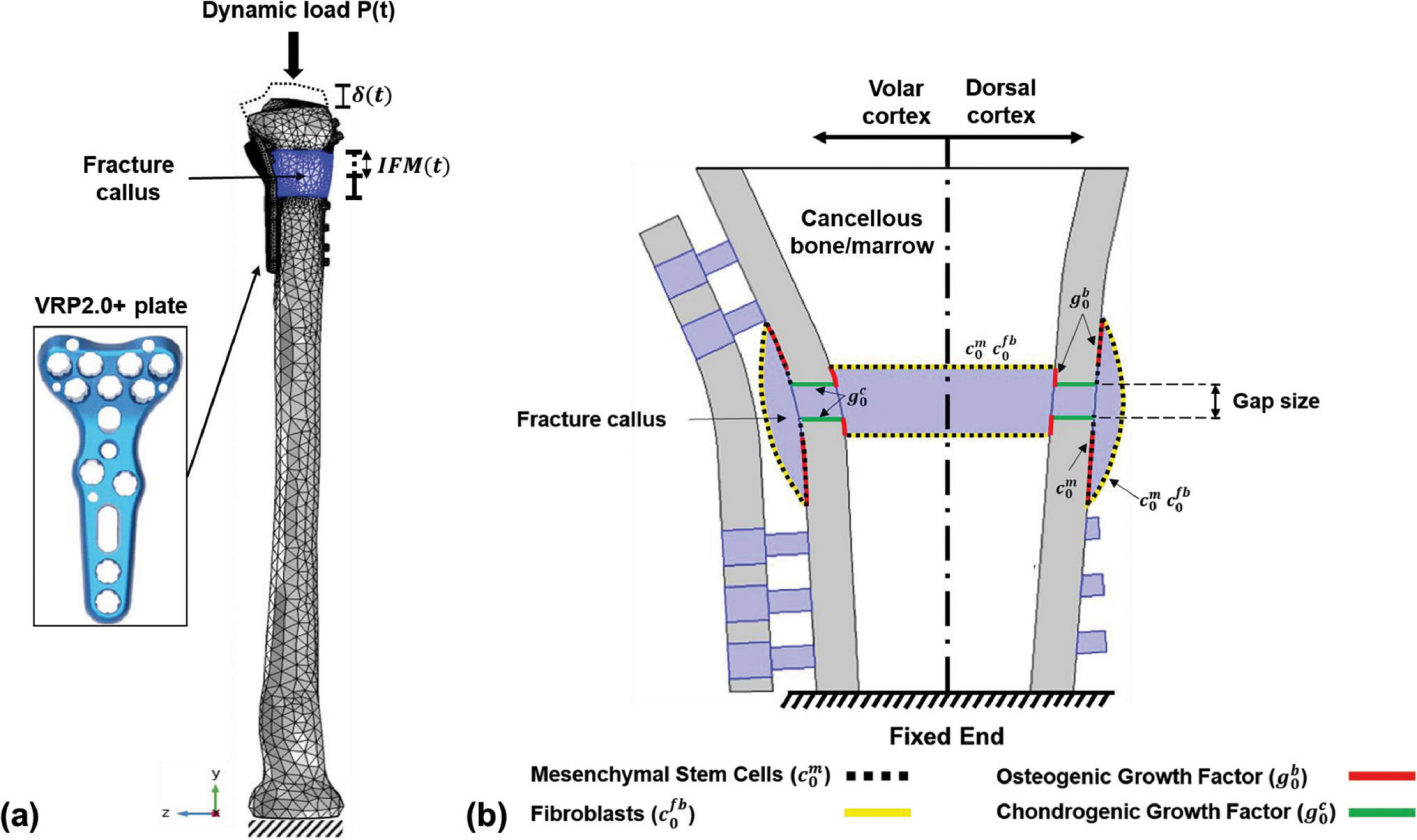
Schematic representation of the DRF model: **a** 3D FEM of a distal radius fracture stabilized with a VRP2.0+ plate; **b** Callus region at the fracture site. Adapted from Liu et al. (2022)

Liu et al. (2022) developed a 3D FEM model in COMSOL MULTIPHYSICS (v.5.6, COMSOL) to study how dynamic grip-exercise loading influences distal radius fracture healing with volar locking plate fixation (Fig. 12). Their model incorporates poroelasticity and a mechanobiochemical regulatory framework to simulate early-stage MSC differentiation, solute transport, and cellular migration within a CT-based geometry. They design a mesh comprising $\approx$ 65,000 tetrahedral elements for cortical bone, callus, marrow, and implant. Their study allowed to predict a strong positive linear relationship between loading rate and axial stiffness of the fixation construct, indicating that higher loading rates induced by rehabilitation exercises reduce maximum displacement (up to 17.5%). Moderate dynamic loading frequencies (1–2 Hz) were found to enhance fracture healing by promoting secondary bone formation, whereas frequencies above 3 Hz can result in detrimental effects. It was also found that dynamic loading at 2 Hz notably improved the transport of cells and growth factors, especially chondrocytes (up to 1700% volarly, and 1300% dorsally) and fibroblasts (up to 100%) within the fracture callus, with more pronounced effects in the dorsal cortex. This enhancement was also found dependent on fracture gap size, with a 3 mm gap combined with 2 Hz loading optimizing chondrocyte concentration and indirect bone healing. Validation was performed by mechanically testing five radius sawbones specimens, fixed with volar locking plates under cyclic axial compression to simulate therapeutic exercises. Experimental data of axial displacement and stiffness at different loading frequencies (2–600 N/s; 1–3 Hz) allowed to identify a regression line fitting the numerical predictions. Such close alignment between experimental and predicted data demonstrated a good model accuracy. However, this work was only focus on axial loading, disregarding bending and torsion forces present *in vivo*. The investigation employed a fixed loading magnitude (100 N hand gripping) and measured the overall axial displacement rather than local IFM. While IFM is considered a more direct metric for evaluating the mechanical environment at the fracture site (Ganadhiepan et al. 2019; Miramini et al. 2016; Liu et al. 2021), the authors justified that, under their strictly controlled experimental setup, the measured axial displacement could reasonably approximate IFM. Assumptions on boundary conditions and early-stage parameters may not sufficiently capture the biological complexity, and temporal changes in callus mechanical properties were also not modeled.

#### Mechanoregulation and tissue differentiation

Mechanoregulation models frequently use biophysical characteristics, such as strain and fluid shear stress, to predict tissue patterning and cellular activity.

**Fig. 13:**
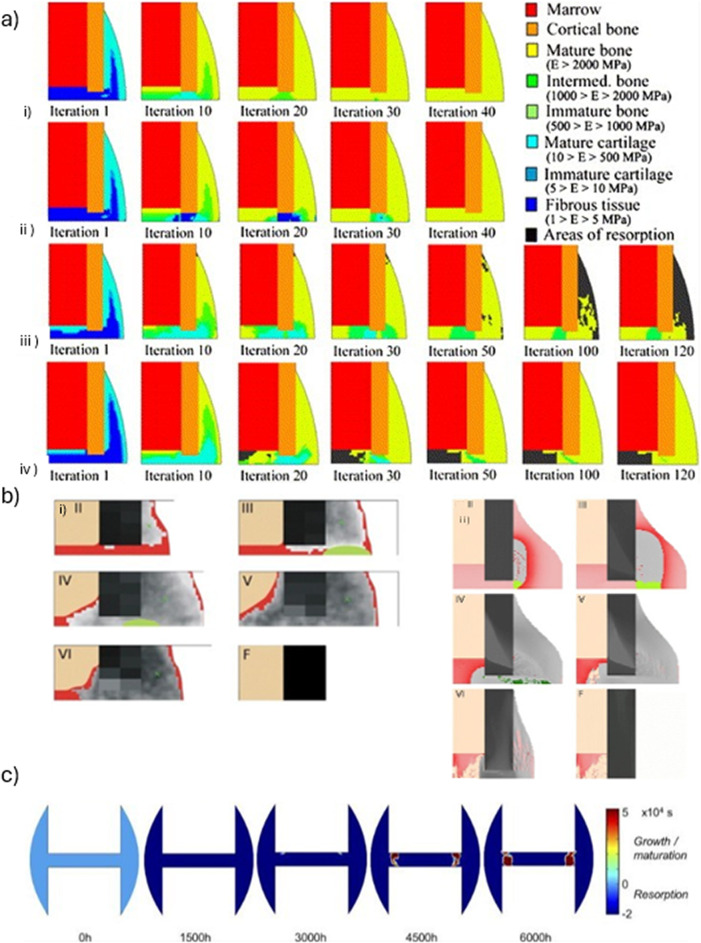
**a** Predicted fracture healing pattern with the biomechanics feedback loading regime with the algorithms of (i) Carter et al. (1988), (ii) Claes and Heigele (1999), (iii) Prendergast et al. (1997) and (iv) Deviatoric strain. Adapted from Isaksson et al. (2006). **b** (i) Experimental tissue patterns averaged from sheep across healing stages II to VI, plus an idealized final cortex restored; (ii) Corresponding simulated tissue distributions obtained, which incorporates endochondral and intramembranous ossification, as well as late-stage bone resorption. The model captures periosteal bone initiation (stage II), cartilage bridging (stage III), gradual ossification and callus reduction (stages IV–VI), and eventual restoration of cortical bone geometry and density (**f**). Adapted from Repp et al. (2015). **c** Bone-specific strain response function shown within its activation range. Other tissue types exhibited constant minimal response, leading exclusively to resorption and are therefore omitted. Adapted from Perier-Metz et al. (2022)

Isaksson et al. (2006) implemented a mechanoregulated axisymmetric FEM model of an ovine tibia with a 3 mm fracture gap and external callus to compare three previously proposed algorithms for mechanobiological tissue differentiation (Carter et al. 1988; Claes and Heigele 1999; Prendergast et al. 1997), as well as to investigate the individual contribution of deviatoric strain, pore pressure, and fluid velocity. Their model comprises 779 elements for the callus, 1060 for the marrow, and 540 for the cortical bone, using 8-node poroelastic elements in ABAQUS (v. 6.3, Dassault Systèmes) with adaptive mechanobiological simulation implemented in MATLAB (v 6.5, MathWorks). Tissue formation was regulated by mechanical stimulus distributions and cell diffusion over 120 iterations (days). Two loading regimes were compared: a constant 300 N axial load and a biofeedback-regulated loading that increased based on decreasing interfragmentary motion, up to 600 N. The three classical algorithms provided accurate predictions of spatial and temporal aspects of fracture healing, including periosteal intramembranous ossification, central endochondral ossification, and creeping substitution. However, only the model developed by Prendergast et al. (1997) predicted callus resorption. When individually tested, only deviatoric strain could reproduce realistic healing patterns, whereas pore pressure and fluid velocity alone failed to reproduce consistent spatial-temporal tissue distributions. However, these models disregarded cellular processes (e.g., apoptosis, mitosis), tissue shape change, and nutrient/vascular regulation. The simulation time was not physically accurate, due to simplifications in cell behavior, diffusion, and mechanotransduction. Isaksson et al. (2008) improved her previous bone healing model by incorporating cell-phenotype-specific behaviors, allowing a mechanistic coupling between mechanical stimuli and cellular processes, such as migration, proliferation, differentiation, apoptosis, and matrix production for MSCs, fibroblasts, chondrocytes, and osteoblasts. This upgraded model was developed using a poroelastic finite element framework and a mechanoregulation algorithm based on deviatoric strain and fluid velocity, implemented as seven coupled nonlinear PDEs, and solved via a custom formulation in ABAQUS (v. 6.5, Dassault Systèmes). Under physiological axial loading (300 N), such model successfully reproduced key features of fracture healing, including periosteal intramembranous ossification, sequential formation of fibrous tissue followed by cartilage in the fracture gap, progressing to endochondral ossification and bone remodeling. Cartilage formation peaked between days 20–30, and complete formation of bony bridges occurred without isolated islands of ossification. Comparatively, the previous model allowed to predict non-physiological bony islands and excessive cartilage production, because tissue types were assigned based on assumed cell distributions, instead of simulating actual matrix production (Fig. 13a). Their new model also demonstrated predictive ability under perturbed conditions: by increasing the load to 400 N, the bony bridging required took twice as long; at 500 N, healing failed with a persistent fibrocartilaginous state. Simulations of periosteal detachment and impaired cartilage remodeling allowed to predict delayed healing, with external bone bridging was found to occur only after 60 days, and complete healing taking up to 80 days. These results reflect improved biological realism, due to the model’s capability to reproduce both spatially and temporally accurate tissue transitions. Nonetheless, it lacks explicit modeling of vascularization and growth factor signaling and assumes binary cell response thresholds, which may limit its biological response.

Vetter et al. (2012) developed a simplified phenomenological mechanobiological model to investigate the spatiotemporal tissue patterning observed during secondary bone healing. Their model simulates healing in a 3 mm tibial osteotomy in sheep, using a custom 2D lattice-based finite element implementation with rotational symmetry, fibrous tissue as the initial condition, and subsequent differentiation driven by local mechanical strain thresholds, and a diffusing “biological potential” representing progenitor cell activity. Such modeling framework was supported by previous mechanoregulation concepts proposed by Claes and Heigele (1999) and extended by Isaksson et al. (2006, 2008). The mechanobiological algorithm was implemented in MATLAB (v. 7.1, MathWorks), and computation was performed using the open-source Finite Element framework DUNE library (Bastian et al. 2008), accessed as a shared object within the MATLAB routine. A rotationally symmetric grid of 112 $\times$ 120 square elements with side length 0.15 mm was used. Simulations were conducted in daily time steps throughout 60 days. Tissue maturation was represented as a local increase in elastic modulus, with values and rates calibrated using nanoindentation and histological data (e.g., cartilage: 10–500 MPa over 10 days). The model distinguished between intramembranous and endochondral ossification based on two volumetric strain thresholds. These thresholds are adimensional, representing fractions of local volumetric strain tolerated by tissue before differentiation: direct bone formation from 0.005 to 0.15 (step 0.005), and the cartilage formation from 0.01 to 0.3 (step 0.01). The parametric study showed the best agreement with experimental histological images ($\approx$6.2% mismatch) when volumetric strain was used as the mechanical stimulus, the biological potential (relative concentration of progenitor cells) was set to 10 at the periosteum (strong source) and 0.1 at the marrow (weaker source), and the volumetric strain thresholds were 1.5% for direct bone formation and 4% for cartilage formation. Despite reproducing essential aspects of mechanoregulated healing, the model could not capture the spatial shift of cartilage toward the endosteal side at later stages, likely due to simplifications such as rotational symmetry, linear elasticity of soft tissues, and lack of explicit bone remodeling. Nevertheless, this study demonstrates that a minimal model, with careful calibration of spatial information, stimulus thresholds, and maturation rates, can effectively predict the main features of secondary bone healing, providing a quantitative platform for investigating the effects of mechanobiological regulation.

Repp et al. (2015) proposed a phenomenologic-based mechanobiological model to explore how specific cellular-level regulatory mechanisms influence tissue patterning during secondary bone healing. Their model allows to simulate a 3 mm tibial osteotomy in sheep using a 2D lattice-based FEM framework with rotational symmetry and volumetric strain as the only mechanical stimulus. Building upon the model by Vetter et al. (2012), the model developed by Repp et al. (2015) incorporates additional features, such as bone resorption governed by a fixed strain threshold ($60~\mu \varepsilon$), and models healing as an iterative loop implemented in MATLAB (MathWorks) with mechanical steps computed via DUNE (Bastian et al. 2008). The tissue state dynamics in each volume element was defined using three thresholds: direct bone formation, endochondral ossification, and resorption. Repp et al. (2015) tested three biologically motivated hypotheses: (1) a delayed response to mechanical stimulation, (2) variable mechanosensitivity via fuzzy thresholds, and (3) committed tissue maturation independent of ongoing stimulation. For each scenario, parameter re-tuning of parameters related to direct bone formation and endochondral ossification was performed to optimize the agreement with a set of six averaged histological images obtained from 32 animals. Simulations allowed prediction of high robustness across scenarios, with all results exhibiting tissue pattern evolution in the correct temporal sequence; a mismatch only between 4 and 11% was reached (Fig. 13b). Differences were mostly observed in cartilage amount and spatial distribution, especially under delayed stimulus or committed maturation. The model allowed to predict that mechanoregulated healing is a robust bio-phenomenon to variations in cellular-level assumptions, provided appropriate threshold tuning is performed. However, limitations include the use of a 2D symmetric geometry, assumption of linear elasticity, and lack of explicit vascular signaling dynamics.

Quinn et al. (2022) developed a coupled computational framework to simulate both the early healing and long-term remodeling phases of tibial fractures, focusing on the mechanobiological effects of internal fixation using for titanium plates. Their model was implemented in ABAQUS (Dassault Systèmes), employing $\approx$ 65500 8-noded linear brick elements across the cortical bone, fracture callus, and fixation plate, with custom subroutines to govern mechanobiological healing and strain-driven remodeling. A simplified cylindrical tibia geometry was used, with fracture gaps ranging from 0.75 to 12 mm. Validation against experimental sheep data showed excellent agreement for interfragmentary motion (differences < 0.1 mm) and spatial callus progression. Simulations allowed to reveal that plate fixation supported fracture bridge formation under high-load conditions (e.g., healing under $\approx$6.6 MPa vs failure at $\approx$0.33 MPa without fixation), but also cause protection against long-term stress, particularly in the anterior cortex, leading to bone resorption in the stress concentration regions where screws were fastened to the bone. Although the framework was able to predict post-union healing and more realistic adapting behavior than uncoupled models, limitations include the idealized geometry, single daily load cycle, and omission of biological and anatomical variability.

Perier-Metz et al. (2022) developed a 3D *in silico* multitissue evolution model to investigate mechanobiological regulation during the late phase of fracture healing, focusing on bone callus remodeling. The model was implemented in COMSOL MULTIPHYSICS (v.5.6, COMSOL) and used tetrahedral finite elements to simulate two distinct animal models: murine (0.5 mm defect) and ovine (3 mm defect). The mesh resolution ranged from 0.16 mm in the inner callus (mouse) to 3.8 mm in peripheral cortical regions (sheep), accounting wit $\approx$ 92,000 elements across bone fragments, cartilaginous callus, and fixation devices. Biophysical inputs included compressive and antero-posterior bending loads (1.5 N and 0.3 N for mouse, respectively; 1200 N and 75 N for sheep, respectively) applied at the proximal end of the bone, reconstructed from *in vivo* experiments. Mechanical stimuli were computed using accumulated strain, which governed tissue maturation and resorption via a mechanoregulation-based reaction–diffusion framework. Model predictions of spatial tissue distribution were qualitatively validated with histological sections at days 14–21 (mouse) and weeks 6–9 (sheep), showing good agreements in the localization of bone formation, external callus resorption, and reopening of the medullary cavity (Fig. 13c). Simulations allowed to predict that bone formation occur primarily under compressive strains around 800 $\mu \varepsilon$ (mouse) and < 1000 $\mu \varepsilon$ (sheep), while bone resorption is triggered by strain accumulation in peripheral and medullary regions. Final predicted mineralized tissue volumes in the sheep model was found to stabilize around 45% after initial loss. Despite its biological relevance, this model presents some limitations, including unrealistic simulation durations (e.g., 225 days in simulation represent 21 days biologically in sheep), and simplified isotropic linear elastic tissue behavior; the cortical bone remodeling and vascular effects were also disregarded. Nonetheless, the framework successfully offers the ability to replicate conserved remodeling patterns across species, being a promising computational tool for studying long-term bone healing.

Moore et al. (2014) developed a mechanistic, multiscale model of bone regeneration that couples mechanical stimuli, progenitor cell dynamics, and BMP-mediated signaling to simulate endochondral ossification in large postnatal bone defects. Their model integrates a 3D finite element framework implemented in ANSYS (ANSYS Inc), using a minimum of 150k quadratic tetrahedral elements to compute deviatoric strain distributions within the defect. The simulated defect geometry was able to reproduce a 2.54 cm mid-diaphyseal segmental bone loss in the ovine femur, stabilized with a stainless steel intramedullary (IM) nail, measuring 35 cm in length and 12 mm in diameter, and secured proximally and distally by four locking bolts (10 cm long, 7 mm of diameter). Periosteal strains were estimated to range between 0 and 1.2%, modulating the local production of bone morphogenetic protein (BMP-2). BMP-2 transport was modeled via passive diffusion ($3*10^{-3}~\text {cm}^2/\text {day}$), generating spatial gradients that regulate cellular activity. Cellular processes (migration, proliferation, and differentiation of mesenchymal progenitors) were modeled through a system of coupled PDEs, numerically solved in MATLAB (v. R2011b, MathWorks). Cell motility was assumed to be one order of magnitude lower than BMP diffusivity. Though parametric analysis, simulations allowed to reveal that cartilage formation was most sensitive to the osteoprogenitor differentiation rate and chondrocyte proliferation rate, both estimated from the literature. Model simulations predicted early accumulation of BMP-2 near the periosteum, followed by a decrease as callus stiffness increased, mirroring dynamic signaling environments. Model validation was performed using histological and $\mu$-CT data from experimental studies of periosteum-mediated defect healing in two ovine. Data from the periosteum group was able to predict 87.5% callus filling (3500 $\hbox {mm}^3$ of a 4000 $\hbox {mm}^3$ defect), closely matched by model predictions with a callus infilling parameter of 90%. In this case, bone volume fraction was approximately 40% with periosteum sutured *in situ*, rising to 60% when a periosteum substitute implant was used. Quantitative $\mu$-CT measurements could not be obtained for the latter case due to imaging artifacts caused by the intramedullary nail. While the model captures key mechanobiological feedbacks and agrees with *in vivo* outcomes, intramembranous ossification was disregarded, idealized representations of cell motility and apoptosis were considered, and explicit vascularization or oxygen tension dynamics was not modeled.

**Fig. 14:**
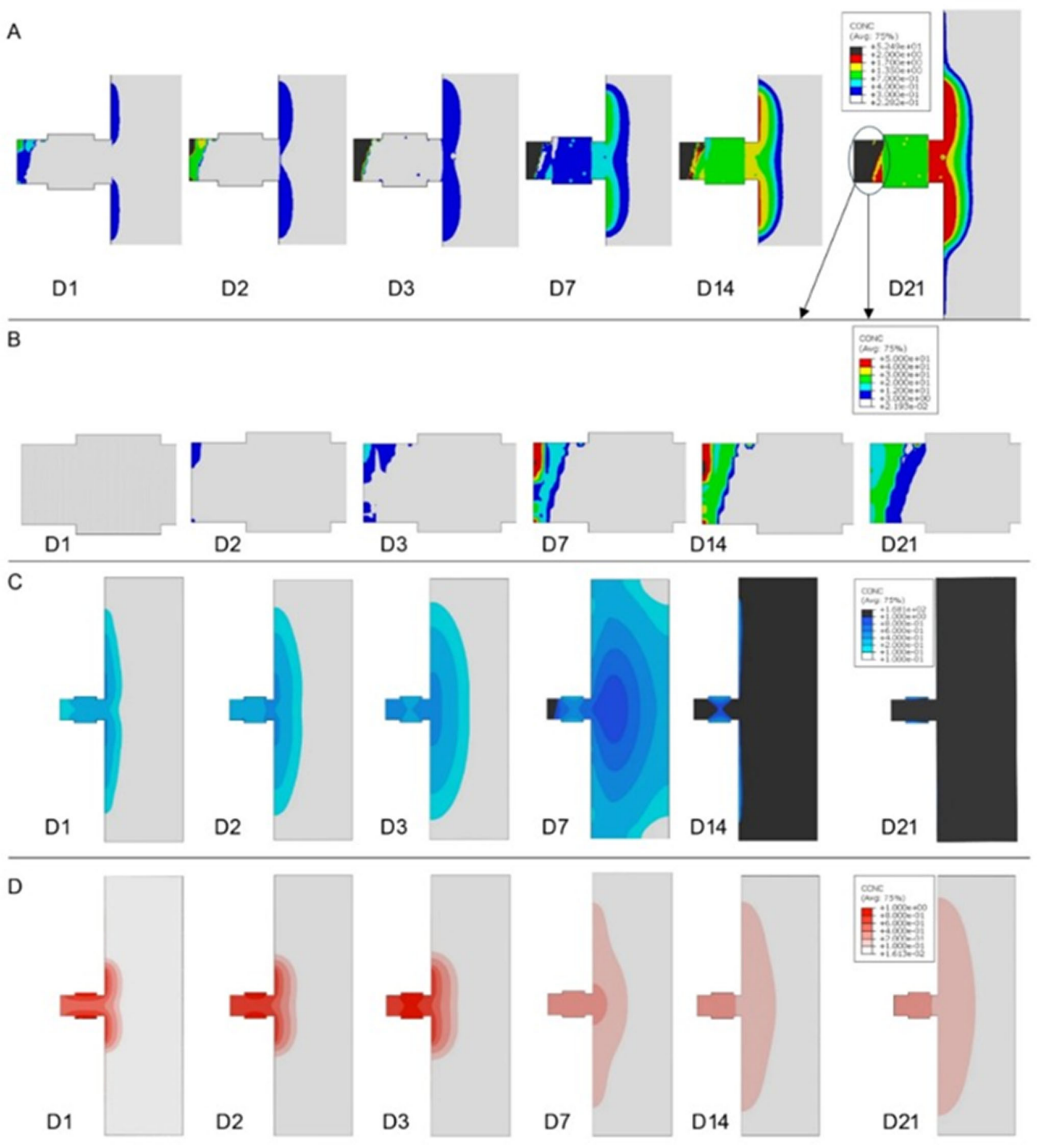
Rows **a** and **b** show the increase in tissue stiffness (E) over time, especially around the cortical bone and under the plate, with hard callus forming by day 21 (10–40 MPa). Row **c** shows MSC migration from high-density areas toward the fracture site, with notable accumulation under the plate by day 7 and saturation between days 7 and 14. Row **d** shows vascularization progression, with blood vessel growth in the callus by day 7 and stabilization of vessel density and oxygen levels by day 21, supporting tissue regeneration. Adapted from Razavi et al. (2024)

A recent study of Razavi et al. (2024) introduced a species-specific computational model designed to simulate bone fracture healing in rats by integrating mechanobiological regulation, angiogenesis, and physiological callus growth. Their model employs a 2D biphasic poroelastic finite element framework built in ABAQUS (v. 2023, Dassault Systèmes), with daily iteration control via Python scripts. The geometry reflects a cross section of a rat femur with a 2 mm transverse osteotomy stabilized using a PEEK plate and screws. A total of 15 108 biquadratic pore pressure elements were used, with mesh refinement focused around the fracture gap. Model inputs included a compressive load of 14.7 N (50% of physiological gait loading) and initial tissue properties obtained from atomic force microscopy (AFM)-based nanoindentation on day one post-surgery. Tissue differentiation was governed by local octahedral shear strain, fluid flow, and oxygen-dependent angiogenic diffusion, simulating MSC-driven transitions into fibroblastic, chondrogenic, and osteogenic lineages. Throughout the 21-day simulation, mechanical stimuli was found to progressively decrease as tissue matured; octahedral shear strain in cortical regions was also found to decrease from 3.7% to 2.0%, and fluid velocity to decrease to the range 30–60  $\mu$m/s. Simulated tissue stiffness beneath the plate increased from early soft tissue values to a range of 10–40 MPa by day 21, indicating early-stage hard callus formation (Fig. 14). Moreover, simulated tissue stiffness in the central region increased from 46 kPa to 853 kPa and in the peripheral region from 235 kPa to 13.33 MPa by day 21. Model validation was performed through direct comparison with histological analysis and AFM measurements across six time points (days 1, 2, 3, 7, 14, and 21), focusing on three predefined regions (central, intermediate, and peripheral). The model was able to accurately predict temporal trends in tissue transformation and stiffness evolution, with elastic modulus predictions in agreement with experimental data until day 14. Model performance was quantified using the root-mean-square error (RMSE), which ranged from $\approx$0.025 in the central region to 0.39 in the peripheral region. Deviations observed beyond day 14 remained within experimental standard deviations. Compared to traditional models based on large animals (e.g., sheep), this rat-specific approach significantly improved the prediction of early-stage mechanical behavior (p < 0.001 for slope differences). Even though this modeling approach was based on simplified 2D geometries, it only includes small sample size (n = 4 per time point), and it is supported by an open-fracture surgical model. It is also relevant to notice that temporal inaccuracies related to cartilage formation between simulation and histology (day 7 vs day 14) suggest the need for further refinements in biological modeling, particularly regarding early chondrogenesis.

#### Advanced computational approaches using fuzzy Logic

Recent developments in computer modeling have included more sophisticated algorithms and numerical methods for simulating the complex processes occurring throughout bone healing. These approaches attempt to overcome the limitations of classic mechanobiological models by integrating dynamic and nonlinear interactions between mechanical and biological factors.

**Fig. 15:**
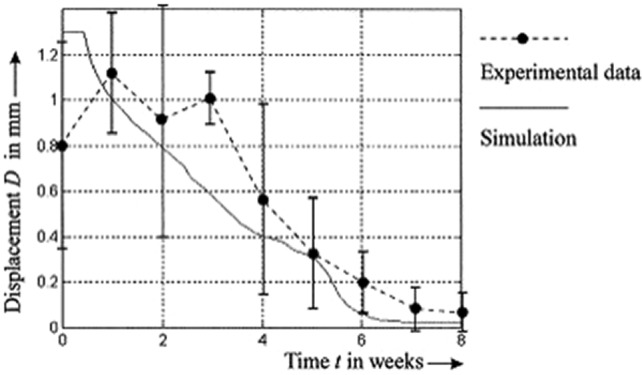
Comparison between simulated and experimentally measured displacement *D* of the osteotomy gap over the first 8 weeks of healing. Experimental data are from in vivo sheep metatarsal models stabilized with external fixation, showing good agreement with the fuzzy logic-based simulation. Adapted from Ament and Hofer (2000)

Ament and Hofer (2000) developed a fuzzy logic-based model of fracture healing to simulate the tissue transformation in a sheep metatarsal osteotomy stabilized with external fixation. Their model combines finite element analysis and fuzzy control theory to predict the spatial and temporal evolution of bone, cartilage, and fibrous tissue within a quadrant geometry of the osteotomy site. The finite element mesh was designed with 3600 triangular elements with rotational symmetry, and simulations were implemented in custom MATLAB (MathWorks) routines. Material properties were interpolated using fuzzy membership functions based on the local composition of the three basic tissues, and the mechanical stimulus was defined by the strain energy density, while a novel osteogenic factor, based on stress gradients, modulated the ossification mode (intramembranous or endochondral). External axial loading was applied up to 500 N, and tissue evolution was updated using Euler integration at daily time steps. Such model allowed to reproduce characteristic healing stages: cartilage bridge formation by day 3, callus growth and progressive ossification between weeks 2–5, and callus resorption beginning after week 8. Quantitatively, displacement of the bone ends was predicted to decrease from 1.2 mm to near zero over 8 weeks, matching experimental data from *in vivo* measurements in sheep (Fig. 15). This agreement supports the model’s ability to predict the dynamics of tissue adaptation. However, limitations include the use of only three tissue types, as well as and the absence of explicit modeling of vascularization, hematoma, or cellular signaling pathways.

Morgan et al. (2024) developed a strain-driven computational algorithm to simulate bone fracture healing in an ovine metatarsal model by directly incorporating maximum and minimum principal strains as mechanical stimuli for tissue differentiation. Unlike traditional models that rely on diffusion-based proxies for angiogenesis or cellular distribution, this modeling approach avoids explicit spatial predefinitions of ossification pathways, thereby ensuring independence from the callus domain. Their model was implemented in MSC.Marc (v2021, MSC Software) using a 2D axisymmetric nonlinear finite element framework representing a transverse mid-diaphyseal osteotomy stabilized by an external fixator. The geometry consisted of a hollow cylindrical metatarsus (outer diameter: 16 mm; cortical thickness: 2 mm) with an explicitly defined callus region (diameter: 48 mm; length: 52 mm), meshed with linear triangular elements (edge length $\approx$0.35 mm). Each callus element was modeled as a mixture of soft tissue, cartilage, and woven bone, with material properties updated over 150 iterations using a fuzzy logic controller implemented in Python (v. 3.6) with the Scikit-Fuzzy toolbox. This controller integrated the local maximum and minimum principal strains, distortional strain, and neighborhood tissue composition to regulate biological transitions during secondary fracture healing. The model allowed to simulate both intramembranous ossification at the periosteum and endochondral bridging across the outer callus. Six distinct fracture conditions were simulated (1 mm gap and 7 % interfragmentary strains (IFS); 1 mm gap and 31% ifs; 2 mm gap and 7% ifs; 2 mm gap and 31% ifs; 6 mm gap and 7% ifs; 6 mm gap and 31% ifs), which correspond to experimental *in vivo* tests carried out in animal (ovine) studies (Claes et al. 1997). The first five groups converged within 55–136 iterations, while the last group, which experimentally exhibited non-union, also failed to converge within 150 iterations. IFM progression curves mirrored experimental data, suggesting a correspondence of $\approx$3 simulation iterations per day of physiological healing. Virtual mechanical testing performed on the final 3D geometries yielded bending stiffnesses of 6.9–8.4 N$\cdot$m/mm in the healed groups and 1.8 N$\cdot$m/mm in the non-union case, which was found not so consistent with experimental trends (8.9–35.6 N$\cdot$m/mm for union, and 1.6 N$\cdot$m/mm for non-union scenarios). This model was able to distinguish between union and non-union scenarios and reproduced key ossification pathways without reliance on predefined spatial patterns. However, the assumption of isotropic behavior of tissues was a modeling limitation, as well as the use of a simplified 2D axisymmetric geometry excluding torsional and shear effects, the use of static axial loading, and absence of bone remodeling (woven-to-lamellar substitution), which most likely contributed to underestimate the stiffness magnitudes.

#### Advanced computational approaches using artificial intelligence

Recent advances in artificial intelligence (AI)/machine learning (ML) have demonstrated promising results in increasing diagnostic accuracy and treatment planning for bone fractures.

**Fig. 16:**
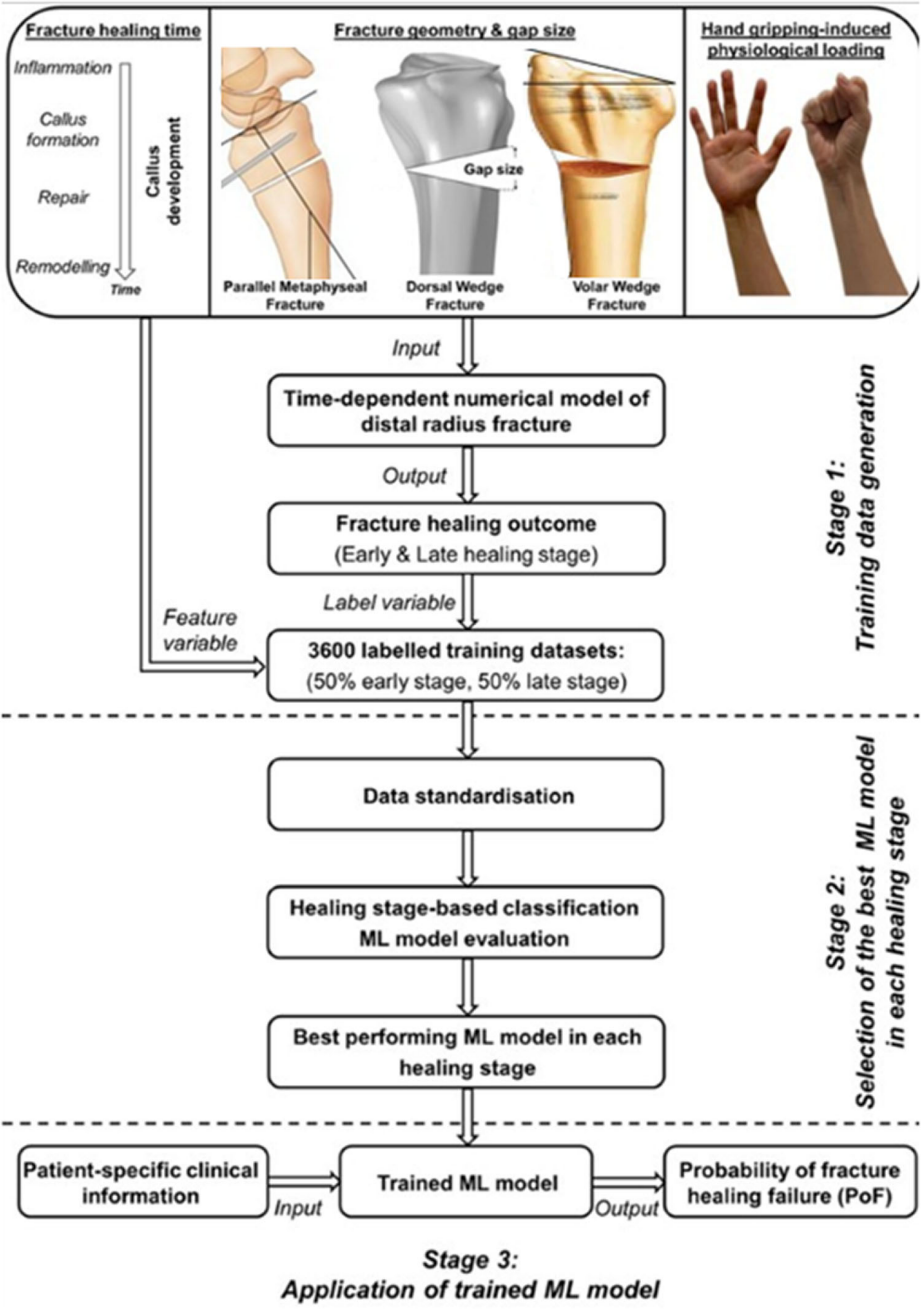
Schematic overview of the methodology by Liu et al. (2023), showing the integration of the numerical DRF model with ML-based classification, trained on 3600 simulated outcomes across different fracture types, gap sizes, and loading scenarios. Adapted from Liu et al. (2023)

Liu et al. (2023) developed a 3D numerical model to simulate distal radius fracture (DRF) healing in the presence of volar locking plate fixation, under gripping-induced physiological loading. Their model integrates mechanoregulated tissue differentiation, angiogenesis, and variable fracture geometry to assess the effect of different loading conditions on healing progression (early and later stages). Simulations considered gap sizes ranging from 1 mm to 5 mm, and multiple fracture types (Colles, Smith, and metaphyseal), evaluating tissue patterns and mechanical thresholds relevant for safe rehabilitation. The computational framework was implemented in COMSOL Multiphysics (v. 6.0, COMSOL Inc.), coupling structural mechanics and transport of diluted species modules to solve for tissue evolution and angiogenesis over time. A total of 3600 simulation outcomes were generated to train and test ML classifiers for predicting healing success or failure (Fig. 16). These datasets, combining four fracture-related features with corresponding healing outcome labels, were imported into MATLAB (R2022a, MathWorks) and processed in the Classification Learner Toolbox. Within this platform, five types of supervised algorithms were tested: support vector machines (SVM), artificial neural networks (ANN), k-nearest neighbors (KNN), decision trees (DT) and ensemble learning, using hyperparameter tuning and fivefold cross-validation, with an 80/20 train–test split. Each classifier was trained on 1440 simulations and tested on 360 unseen cases, per stage, with cross-validation applied to mitigate overfitting. Given the unbalanced dataset, performance was assessed not only by accuracy but also by precision, recall, and F1 score, prioritizing recall to minimize false negatives, which would correspond to undetected healing failures. Results showed that the SVM with cubic kernel achieved superior performance in early-stage healing (98% accuracy, 94% recall and F1 score 92%), reflecting the relatively straightforward patterns in early tissue differentiation. In contrast, a tri-layered ANN yielded the best results in later stages (95% accuracy, 87% recall and F1 score 88%), reflecting its ability to predict nonlinear interactions between fracture geometry, gap size, and physiological loading over time. This performance pattern suggests that early-stage healing outcomes are easier to classify using models that performs best with simpler decision boundaries, whereas later-stage healing involves more complex relationships that benefit from the flexible, nonlinear modeling capacity of neural networks. This workflow demonstrated that while finite element simulations required approximately six weeks of computation, the subsequent ML training took less than a minute, underlining the efficiency and clinical scalability of the hybrid FEM–ML pipeline. In the periosteal callus, simulations allowed to predict that bone tissue decreased from 86% to 78% as gap size increased from 1 mm to 5 mm, while fibrous tissue increased from 3% to 20%, indicating compromised healing in larger gaps. Regarding angiogenesis, the proportion of periosteal callus under favorable strain conditions ($<6\%$) was found to decrease from 95% to 77%. Model validation was performed by comparing predicted gripping strength recovery to clinical experimental data from four studies, showing strong agreement (Oshige et al. 2007; Tosti and Ilyas 2013; Takeuchi et al. 2016; Luo et al. 2018): at week 4, the model predicted 46% recovery (normalized to 290 N), consistent with reported values of 45–50%, and by week 12, the model reached 87%, matching clinical measurements (80–90%). Tissue-level predictions also reproduced key histological features, including periosteal intramembranous ossification, central endochondral ossification, and fibrous tissue in high-strain regions. Despite these strengths, this model assumes constant loading conditions and simplified time-dependent tissue properties. Moreover, it does not incorporate patient-specific variability in callus mechanics or evolving fracture geometries and relies on idealized fracture parameters due to limited availability of detailed clinical data. The ML classifiers, while efficient, remain purely statistical and cannot capture the underlying biophysical mechanisms of healing, as well as additional clinical factors, such as diabetes or osteoporosis that were not considered.

Razavi et al. (2025) developed a ML surrogate model as a rapid alternative to a previously validated 21-day finite element mechanoregulation framework for simulating femoral fracture healing in rodents (Razavi et al. 2024). The original FEM model incorporated a poroelastic biphasic formulation to represent both solid and fluid phases of healing tissue, with tissue differentiation governed by mechanical stimuli, specifically octahedral shear strain and fluid velocity, as well as vascular cues modeled through vessel density and MSC migration. Simulations were conducted on a simplified 2D hollow cylindrical femoral shaft with a 2 mm transverse osteotomy and variable fixation parameters, using Python-controlled iterative analysis. Material properties evolved according to tissue type (fibrous tissue, cartilage, woven bone, lamellar bone), generating a total of 648 FE simulations by systematically varying clinically relevant parameters, including plate stiffness and length, screw number, applied compressive and shear loading, osteogenic differentiation rates, and bone condition (healthy versus osteoporotic). Outputs including central (CS), intermediate (IS), and peripheral (OS) callus stiffness, and total strain energy (SE) over 21 days, were used to train and compare 7 ML models (SVR, RF, XGBoost, MLP, 1D CNN, RNN, and LSTM). Hyperparameters were optimized via Bayesian optimization using the Tree-structured Parzen Estimator (TPE) algorithm. Model performance was evaluated through tenfold cross-validation, including tests on reduced training sets (down to 50%) to assess data efficiency. The sequence-to-sequence LSTM model achieved the highest predictive accuracy, reducing errors by up to 98% compared to other methods, and maintaining robust performance even with limited data. Beyond model training, Razavi et al. (2025) assessed the ability of this model to generalize to unseen scenarios. Among all tested models, the top performers (LSTM, RNN, XGBoost, and CNN) demonstrated strong interpolation capabilities, successfully predicting callus stiffness and capturing spatial differences across CS, IS, and OS regions despite having no prior exposure to those parameter combinations. Importantly, only 2 models provided high accuracy when extrapolating to later healing stages. LSTM and XGBoost consistently achieved the lowest mean squared errors (MSE), with average values of 0.01–0.002 and 0.03–0.10, respectively, across a 10-day prediction horizon, and even lower errors for one-day-ahead forecasts (LSTM: 0.006–0.020; XGBoost: 0.025–0.070). The superior performance of the sequence-to-sequence LSTM can be attributed to its inherent ability to capture long-range temporal dependencies within the healing trajectory, allowing it to more accurately predict progressive changes in callus stiffness compared to feedforward and tree-based models. This advantage became particularly evident in extrapolation tasks, where sequential modeling facilitated robust forecasting of future outcomes. SHAP (SHapley Additive exPlanations) revealed the magnitude and directionality of input feature contributions. Screw number was the dominant predictor of central stiffness, with higher screw counts increasing fixation stability and promoting mineralization, whereas excessive compression loading negatively impacted peripheral healing. Plate size and stiffness were critical for intermediate stiffness, and shear loading predominantly influenced strain energy, reflecting the importance of multidirectional forces in governing global mechanical behavior. Analysis of predicted callus geometry showed that larger plates and higher screw counts were associated with smaller callus dimensions, consistent with clinical and experimental evidence (Khanuja et al. 2014) that controlled micromotion promotes callus formation while excessive rigidity can induce stress shielding. The model relies on simplified 2D geometry, lacks direct experimental or clinical validation, and depends on synthetic training data. Despite these constraints, this study presents a robust and computationally efficient ML-enhanced modeling strategy that holds promise for real-time clinical decision support, implant optimization, and personalized fracture management. Future work will most likely be focused on developing anatomically accurate 3D patient-specific models and validating predictions with experimental and clinical data to enhance translational potential.

#### Advanced computational approaches using specimen-specific models

Specimen-specific finite element analysis has emerged as an effective approach for increasing the complexity of computational models. By combining patient-specific anatomical and biomechanical data, these models provide more personalized predictions of healing outcomes.

**Fig. 17:**
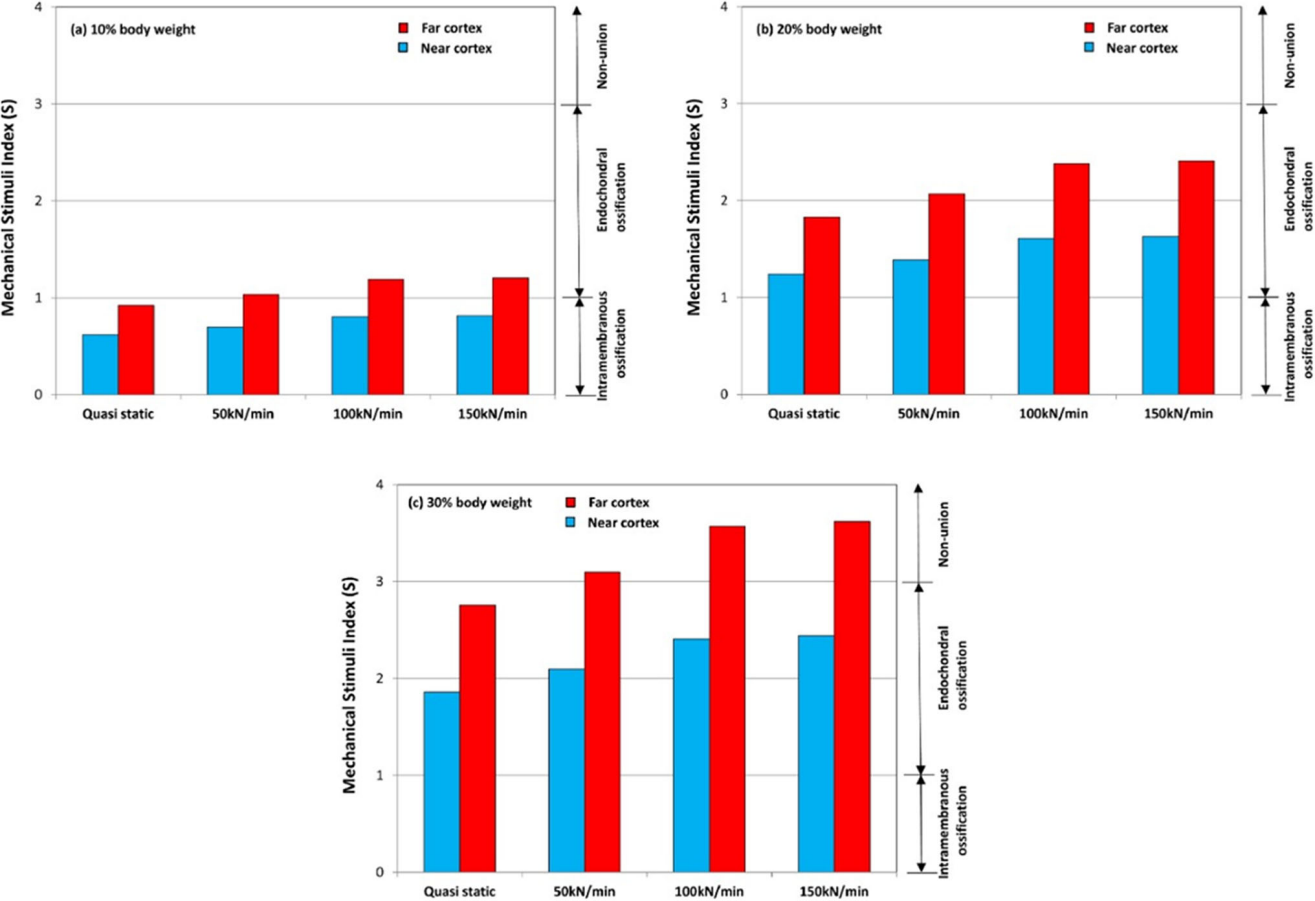
Mechanical stimuli index (*S*) at near and far cortex regions of the fracture site. **a** 10% body weight, **b** 20% body weight, and **c** 30% body weight. Adapted from Lihai et al. (2017)

Lihai et al. (2017) developed a multiscale framework to assess how partial weight-bearing exercise influences bone fracture healing under different walking speeds and loading rates. Their study combined wearable accelerometry, mechanical testing, and finite element modeling. Firstly, triaxial acceleration data were collected from healthy individuals walking at speeds from 1 to 12 km/h using waist-mounted ActiGraph wGT3X-BT sensors. A theoretical model was formulated to estimate peak ground reaction force (PGRF) and peak loading rate (PLR) from the vertical acceleration signal. These quantities were related to raw acceleration via fitted empirical relations a Hill function for PGRF ($\hbox {R}^2$ = 0.97), and a linear expression for PLR ($\hbox {R}^2$ = 0.99). Interestingly, PGRF stabilized at walking speeds above 6 km/h (approximately six times BW), whereas PLR increased almost linearly with velocity, highlighting distinct mechanical effects of speed and loading rate on the healing environment. These estimates were then applied as input loads in mechanical tests using surrogate tibiae stabilized with locking compression plates. IFM and fixation stiffness were measured under increasing loading rates (50–150 kN/min) and axial forces corresponding to 10%, 20%, and 30% of average body weight (750 N). Results allowed to predict that increasing loading rate significantly affected construct mechanics: axial stiffness was found to reduce by up to 27%, resulting in IFM increases of up to 53%. This finding indicates that faster loading rates, as occurred at higher walking speeds, may compromise fixation stability and increase the risk of delayed union. These experimental values were incorporated into a computational model implemented in COMSOL Multiphysics (COMSOL Inc.), where early-stage callus tissue was modeled as a biphasic poroelastic material using a neo-Hookean solid matrix and Darcy flow. The FE mesh included over 46 000 tetrahedral elements, allowing detailed spatial resolution of local tissue mechanics. Tissue differentiation was simulated using a mechanical stimulation index dependent on octahedral shear strain and interstitial fluid velocity. Model outputs allowed to predict that at 10% BW, intramembranous ossification (mechanical stimulation index: < 1) occurred near the cortex, while endochondral ossification (mechanical stimulation index in the range 1–3) dominated in distal regions. At 20% BW, endochondral ossification was found more widespread, whereas at 30% BW, mechanical overstimulation (mechanical stimulation index: > 3) was observed in far cortex regions, suggesting risk of non-union (Fig. 17). Limitations include the use of synthetic bones rather than biological tissue, model validation was not carried out, and simplified patient-specific gait dynamics based on healthy individuals were used. Moreover, the study included only three healthy volunteers for gait data collection, which restricts generalizability to patient populations with altered walking patterns. Nonetheless, this integrative framework highlights the critical role of PGRF and PLR in modulating early healing outcomes, and offers a foundation for personalized physiotherapy design.

#### Summary

A summary of model types, biophysical inputs, model characteristics, main predictions, validation method, and limitations related to each study focused on the key mechanobiological modeling approaches is presented in Table 3.

**Table 3:** Presentation of mechanobiological models that integrate both mechanical and biological factors to simulate bone regeneration, highlighting the interactions between mechanical stimuli and cellular responses during bone healing

Model type	Biophysical inputs	Model characteristics	Main predictions	Validation method	Limitations	Reference
EM + PDE + Fuzzy logic	Axial load (700 N), interfragmentary strain (7% / 33%), dilatational and distortional strain, tissue fractions, perfusion	Axisymmetric 2D FE model of sheep tibia (ABAQUS) with 2 mm (stable) and 3 mm (unstable) fracture gaps; fuzzy logic rules in MATLAB; PDEs for growth factor (GF) diffusion (BMP-2/4, TGF-$\beta$1)	Stable cases (A, B) showed osteogenic GF arrival at fracture gap in $\approx$4 weeks and complete healing by 4 weeks; unstable cases (C, D) delayed arrival (8–10 weeks) and healing (8–9 weeks); higher IFS increased GF spread but delayed healing	No experimental validation	GF not used as feedback regulator; mixed-species parameters; simplified axisymmetric geometry	Wang and Yang (2019)
FEM	Strain rate (first invariant), muscle and joint forces, biochemical reaction rates	3D FEM in ANSYS; CT-based femur geometry; 5 coupled biochemical reactions; iterative update of density and modulus based on mechanobiochemical stimuli	Severe bone loss under plate (up to -70%); bone gain near screw holes (up to +110%); similar average loss in anterior/lateral plating	Validation based on lateral plate only; predicted losses in posterior (-32%), lateral (-27%) and anterior (-15%) quadrants matched clinical ranges: posterior (-45% to -6%), lateral (-28% to -5%), anterior (-37% to -1%)	No temporal calibration; isotropic material assumption; simplified cellular mechanotransduction; dual-implant case not directly validated	Avval et al. (2016)
FEM	Mechanical stimuli, MSCs, osteoblasts, fibroblasts, chondrocytes; tissue types (fibrous, cartilage, bone)	2D axisymmetric geometry; ABAQUS v6.3 with user-defined subroutines; axial loads (250, 500, 750 N)	Moderate loading promotes endochondral ossification; excessive motion (>2 mm) causes fibrous tissue persistence; callus radial thickness approaches cortical bone	Validation against sheep experiments: interfragmentary motion 0.2–1.5 mm, callus morphology and healing time comparable; healing completed within 8 weeks	2D simplification; simplified growth mechanisms; no endosteal callus; assumptions on cell saturation and matrix production	García-Aznar et al. (2007)
FEM	Mechanical stimuli, MSCs, growth factors (osteogenic and chondrogenic), tissue types (fibrous, cartilage, bone)	2D axisymmetric geometry; using COMSOL, 1933 triangular elements and 20,745 degrees of freedom; cyclic loading (5–15% strain, 0.01–10 Hz, 6 h)	Dynamic loading enhances MSC and growth factor transport; optimal loading (10% strain @ 1 Hz) increases cartilage formation (+15%) and slightly reduces fibrous tissue (–5%), promoting endochondral ossification	Validation against two experimental studies: accurate prediction of tissue distribution at day 7 (fibrous: 90% endosteal, 93% cortical, 65% periosteal; cartilage up to 15%; bone 20% in periosteal zone); total bone ($\approx$16%) and cartilage ($\approx$8.3%) content matched murine tibial fracture data	2D simplification; early-stage only (first week); constant callus properties	Ghimire et al. (2018)
FEM	Octahedral shear strain; Interstitial fluid velocity; MSC diffusion	2D axisymmetric model of titanium dental implant; ANSYS Mechanical APDL; Biot’s poroelasticity theory; cyclic loading ($z_r$, $z_h$); 30-day simulation	$z_h$ + $S_{\textrm{avg}}$ led to bone formation; $S_{\textrm{max}}$ resulted in fibrous tissue; fluid velocity peaked near 0.1–12$\mu$m/s (P1); slower stabilization in P2 and P3	No validation	Linear elastic materials; 2D geometry; No directional influence of fluid flow on MSC migration	Irandoust and Müftü (2023)
FEM	Shear strain; Interstitial fluid velocity; MSC proliferation and migration	3D poroelastic model; Abaqus 54 simulations; Random dispersion cell model; Scaffold with porosity 30–70%, stiffness 750–1250 MPa, dissolution 0–1%/iteration; Loading 1–2 MPa	71% bone regeneration under baseline; High porosity enhances bone but reduces stiffness; High dissolution rates cause collapse; Medium rates optimize outcome; Young’s modulus more critical under high load	No experimental validation	Assumes uniform nutrient supply; excludes apoptosis; linear degradation kinetics	Byrne et al. (2007)
FEM	Hand grip dynamic loading (axial compression, 100 N at 1–2 Hz and >3 Hz); poroelasticity; mechanobiochemical model (MSC differentiation, solute transport, cell migration)	CT-based geometry in COMSOL; $\approx$65,000 tetrahedral elements (bone, callus, marrow, implant); axial dynamic loading at varied rates	Moderate frequencies (1–2 Hz) enhance healing and chondrocyte transport (up to 1700% volarly); 2 Hz with 3 mm gap optimizes indirect bone formation; high frequency (>3 Hz) detrimental	Experimental validation with 5 sawbone specimens under cyclic axial loading; close match between predicted and measured displacement/stiffness	Only axial loading considered; no local IFM measured; simplified early-stage biology; constant loading magnitude; no time-dependent callus properties	Liu et al. (2022)
FEM	Deviatoric strain, pore pressure, fluid velocity, cell diffusion	Axisymmetric 2D poroelastic model of ovine tibia with 3 mm fracture gap; ABAQUS 2379 elements; adaptive mechanobiological algorithm in MATLAB; 120 iterations; axial loading (300 N or biofeedback to 600 N)	All classic algorithms reproduced fracture healing stages; only Lacroix model predicted callus resorption; deviatoric strain alone sufficient for correct prediction; pore pressure and fluid velocity alone are insufficient	Qualitative comparison with previous results	No explicit modeling of mitosis, apoptosis, neovascularization; simplified cell migration; model time not physical	Isaksson et al. (2006)
3D poroelastic FEM	Deviatoric strain, interstitial fluid velocity	Seven coupled nonlinear PDEs governing MSCs, fibroblasts, chondrocytes, and osteoblasts; mechanoregulation drives migration, proliferation, differentiation, apoptosis, and matrix production; implemented in ABAQUS	Reproduced spatially and temporally healing: periosteal intramembranous ossification, sequential fibrous and cartilaginous formation in gap, endochondral ossification; higher loads (400–500 N) delayed or prevented healing; periosteal stripping and cartilage impairment led to delayed bridging	Comparison with previous phenomenological model; qualitative agreement with experimental observations	No vascularization or growth factor modeling; binary cell thresholds; neglect of volumetric growth	Isaksson et al. (2008)
FEM	Volumetric strain; diffusing biological potential	2D lattice-based FEM with rotational symmetry; implemented in MATLAB; mechanical solver via DUNE; 112$\times$120 grid, 1-day steps until 60 days; tissue maturation as modulus increase	Model reproduced periosteal ossification, cartilage bridging, and realistic callus progression; best results with $c=10$ (periosteal), $c=0.1$ (marrow), and thresholds $M_\textrm{Sb}=1.5\%$, $M_\textrm{Sc}=4\%$	Pixel-wise comparison with averaged histological images from 32 sheep tibial osteotomies across 6 healing stages; mismatch $\approx$6.2%	No modeling of bone resorption/remodeling; assumes linear elasticity, rotational symmetry; lacks vascularization and signaling	Vetter et al. (2012)
FEM	Volumetric strain; biological potential; thresholds $\text {MS}_\textrm{b}$, $\text {MS}_\textrm{c}$, $\text {MS}_\textrm{r}$	2D lattice-based grid with rotational symmetry; implemented in MATLAB with mechanical steps via DUNE; iterative update of tissue type and modulus; incorporates bone resorption; tested delayed response, fuzzy thresholds, and committed maturation	Accurately reproduced healing sequence; variations mainly affected cartilage amount/location; committed maturation accelerated healing	Pixel-level comparison with 6 averaged histological images from 32 sheep tibial osteotomies (4–11% mismatch)	Simplified 2D symmetric geometry; linear elasticity; no explicit vasculature or signaling dynamics	Repp et al. (2015)
FEM	Compressive loading 10–300% BW (1 cycle/day); mechanobiological stimulus (deviatoric strain + fluid flow)	Cylindrical tibia, gaps 0.75–12 mm; plated (Ti plate + 6 screws) vs non-plated; $\approx$65k elements, solved in Abaqus with subroutines	Plate enables early union at high loads and low gaps; long-term stress shielding causes cortical resorption. Non-plated models fail under high load	Interfragmentary movement matched sheep osteotomy data (error < 0.1mm) and reproduced spatial callus patterns	Idealized bone–plate geometry; single daily load cycle; isotropic tissues; no vascular or patient-specific factors	Quinn et al. (2022)
FEM	Compressive + flexural loads; principal strain $\varepsilon _I$, $\varepsilon _{III}$; reaction-diffusion tissue evolution	3D model implemented in COMSOL; $\approx$ 92,000 tetrahedral elements; species-specific geometry (mouse: 0.5 mm gap; sheep: 3 mm gap); mesh size: 0.16–3.8 mm	Remodeling driven by strain accumulation; bone formation at $\varepsilon<$ 800–1000 $\mu \varepsilon$; medullary reopening and external callus resorption reproduced; mineralized volume stabilized around 45% (sheep)	Qualitative comparison with histological sections (mouse: d14–21, sheep: wk6–9); spatial pattern of bone and cartilage matched	Simulation slower than biological time; no cortical remodeling; isotropic linear elastic tissue; no vascular modeling	Perier-Metz et al. (2022)
Mathematical with FEM-based strain input	Deviatoric strain, BMP-2 gradients	3D FEM in ANSYS with $\ge$150k quadratic tetrahedral elements simulating a 2.54 cm ovine femoral defect stabilized with an IM nail; BMP-2 diffusion ($D = 0.013$ $\hbox {cm}^2$/day); cell motility $1.3\times 10^{-3}$ $\hbox {cm}^2$/day; cellular dynamics (migration, proliferation, differentiation) solved PDEs in MATLAB	Accurate reproduction of endochondral ossification dynamics; predicted 90% callus infilling, matching 87.5% observed *in vivo*; bone volume fraction range (40–60%) depending on implant	Comparison with $\mu$CT and histology from ovine critical-size defect studies	Exclusion of intramembranous ossification; simplified cell motility and apoptosis; no vascularization or oxygen tension modeling	Moore et al. (2014)
2D FEM	AFM-derived tissue stiffness (day 1), mechanical loading (14.7 N), strain, fluid velocity, MSC diffusion, vascular density	Built in ABAQUS with daily iterations in Python; 15,108 biquadratic pore pressure elements; geometry from rat femur with 2 mm osteotomy stabilized by PEEK plate and screws	Accurately predicted spatial–temporal evolution of callus tissue; simulated stiffness increased to 10–40 MPa by day 21; model outperformed sheep-derived mechanoregulation models	Histological analysis, AFM nanoindentation (3 regions $\times$ 6 time points: days 1, 2, 3, 7, 14, 21); compared predicted vs measured stiffness (RMSE and regression)	2D simplification; small sample size (n=4/time point); time difference in simulated cartilage formation vs histology	Razavi et al. (2024)
FEM + fuzzy logic	Strain energy density *u*(*x*); osteogenic factor *c*(*x*) based on stress gradients	3600 triangular elements (rotational symmetry); implemented in MATLAB; daily time steps via Euler integration; 3 tissue types with fuzzy membership; $F_\textrm{max} = 500$ N	Reproduced cartilage bridging (day 3), ossification (weeks 2–5), and callus resorption (after week 8); displacement decreased from 1.2 mm to 0 mm over 8 weeks	Comparison with experimental displacement data *in vivo*	Only 3 tissue types; no vascularization, hematoma, or signaling pathways	Ament and Hofer (2000)
FEM, strain-based + fuzzy logic	Maximum and minimum principal strain, distortional strain, tissue composition in neighboring elements	2D axisymmetric nonlinear FE model in MSC.Marc; ovine metatarsal osteotomy; external fixator; triangular mesh (0.35 mm); 150 iterations; fuzzy logic controller implemented in Python	Callus domain independence; physiological simulation of intramembranous and endochondral ossification; correct classification of union vs non-union; healing rate of 3 iterations $\approx$1 day	Good IFM progression and not so equivalent virtual mechanical testing compared to *in vivo* experimental data	Assumes isotropic materials; 2D axisymmetry (no torsion/shear); static loading only; no bone remodeling; stiffness magnitudes underestimated	Morgan et al. (2024)
3D mechanobiological + machine learning	Gap size (1–5 mm), fracture geometry (Colles, Smith, metaphyseal), gripping-induced load, healing time (features); healing outcome (success/failure, labels)	COMSOL-based time-dependent model coupling structural mechanics and angiogenesis (transport of species); 3600 simulations; trained ML classifiers (SVM, ANN, KNN, DT, ensemble) with cross-validation	Bone fraction in periosteal callus decreased from 86% to 78% and fibrous tissue increased from 3% to 20% (gap 1–5 mm); SVM best in early stage (accuracy 98%, recall 94%, F1=92%), ANN best in late stage (accuracy 95%, recall 87%, F1=88%)	Comparison with four independent clinical datasets (gripping strength recovery at weeks 4 and 12); model predicted 46% (vs 45–50%) at week 4, and 87% (vs 80–90%) at week 12	Assumes constant loading conditions, simplified time-dependent tissue properties, no patient-specific variability	Liu et al. (2023)
2D mechanobiological + machine learning	Plate stiffness, length, screw number, loading (compression + shear), osteogenic rates, bone health	Poroelastic biphasic 2D FE model (Python); 648 simulations of rat femur with transverse osteotomy; mechanical and biological inputs (shear strain, fluid flow, vessel density); 7 ML algorithms trained on outputs (Central, Intermediate and Outer Stiffness, Strain Energy); sequence-to-sequence LSTM model selected	LSTM reduced prediction error up to 98%, capturing long-range temporal dependencies; SHAP showed screw number $\uparrow$ central stiffness, plate size and stiffness $\uparrow$ intermediate stiffness, shear loading $\uparrow$ strain energy; model generalized well to interpolated inputs and performed reasonably on early-stage extrapolation (MSE: 0.01–0.002 for LSTM)	Computational validation using tenfold cross-validation, data efficiency tests (down to 50%), and generalization/extrapolation to unseen scenarios	Simplified 2D geometry; lack of experimental or clinical validation; dependence on synthetic training data	Razavi et al. (2025)
FEM + empirical loading input	Acceleration-derived PGRF and PLR; IFM; octahedral shear strain	COMSOL poroelastic FEM (neo-Hookean + Darcy flow; 46 000 elements); synthetic tibia mechanical testing; tissue differentiation via stimulation index *S*	Stiffness decreased up to 27%, IFM increased 53%; ossification shifted with BW (intramembranous ossification at 10%, endochondral at 20%, overstimulation/non-union risk at 30%)	No *in vivo* validation; outputs consistent with mechanical test observations	Synthetic bones; only 3 healthy subjects for gait input; simplified gait dynamics	Lihai et al. (2017)

### Computational models related to ultrasound phenomena

In addition to mechanical and biological techniques, advances in ultrasound have introduced a promising tool for monitoring bone fracture healing, by measuring wave propagation velocity across damaged bone tissue (Vavva et al. 2007; Protopappas et al. 2008). This technique enables researchers to timely assess structural changes and healing progression, offering valuable insights into the biomechanical properties of bone fracture healing dynamics. Changes in wave velocity can be directly correlated with tissue differentiation, providing an indirect measure of callus formation, bone mineralization, and mechanical stability at the fracture site (Vavva et al. 2007; Protopappas et al. 2008).

Vavva et al. (2007) developed a 2D computational model to investigate guided ultrasound wave propagation in healing bones, with a focus on the influence of surrounding soft tissues on wave dispersion and first-arriving signal (FAS) velocity. The bone was modeled as a homogeneous isotropic elastic plate with a multiregion inhomogeneous callus representing progressive healing across three stages. Three boundary condition scenarios were examined: full immersion in blood (Model 1), blood over the periosteal surface with marrow beneath (Model 2), and a three-layer model with both blood and marrow layers (Model 3). Wave propagation was simulated using the finite difference method implemented in Wave2000 Pro (CyberLogic Inc.), and analyzed using time–frequency Reassigned Smooth-Pseudo Wigner–Ville (RSPWV) distributions. The predicted FAS velocity for the intact bone was 3914 m/s in Model 1, 3925 m/s in Model 2, and 3772 m/s in Model 3, indicating minimal influence from boundary conditions in Models 1 and 2, but a noticeable reduction in Model 3. Throughout the healing process, the FAS velocity was found to decrease with tissue maturation, approaching intact bone values by Stage 3. Besides, Model 2 revealed the presence of higher-order guided wave modes, specifically the leaky A2 (l–A2) and S3 (l–S3) modes, which was found within the 0.73–1.13 MHz frequency range. These modes were not predicted in the simpler Model 1, where the bone was fully immersed in fluid. This suggests that including realistic representations of surrounding soft tissues (such as finite blood and marrow layers) allows for the emergence of additional wave modes that can enhance the sensitivity of ultrasound-based monitoring. Limitations include the lack of full dispersion curve analysis for Models 2 and 3, (the numerical computation of the corresponding theoretical dispersion curves was still in progress), the simplification of cortical bone geometry as a 2D plate, and absence of anisotropy, porosity, or experimental validation.

**Fig. 18:**
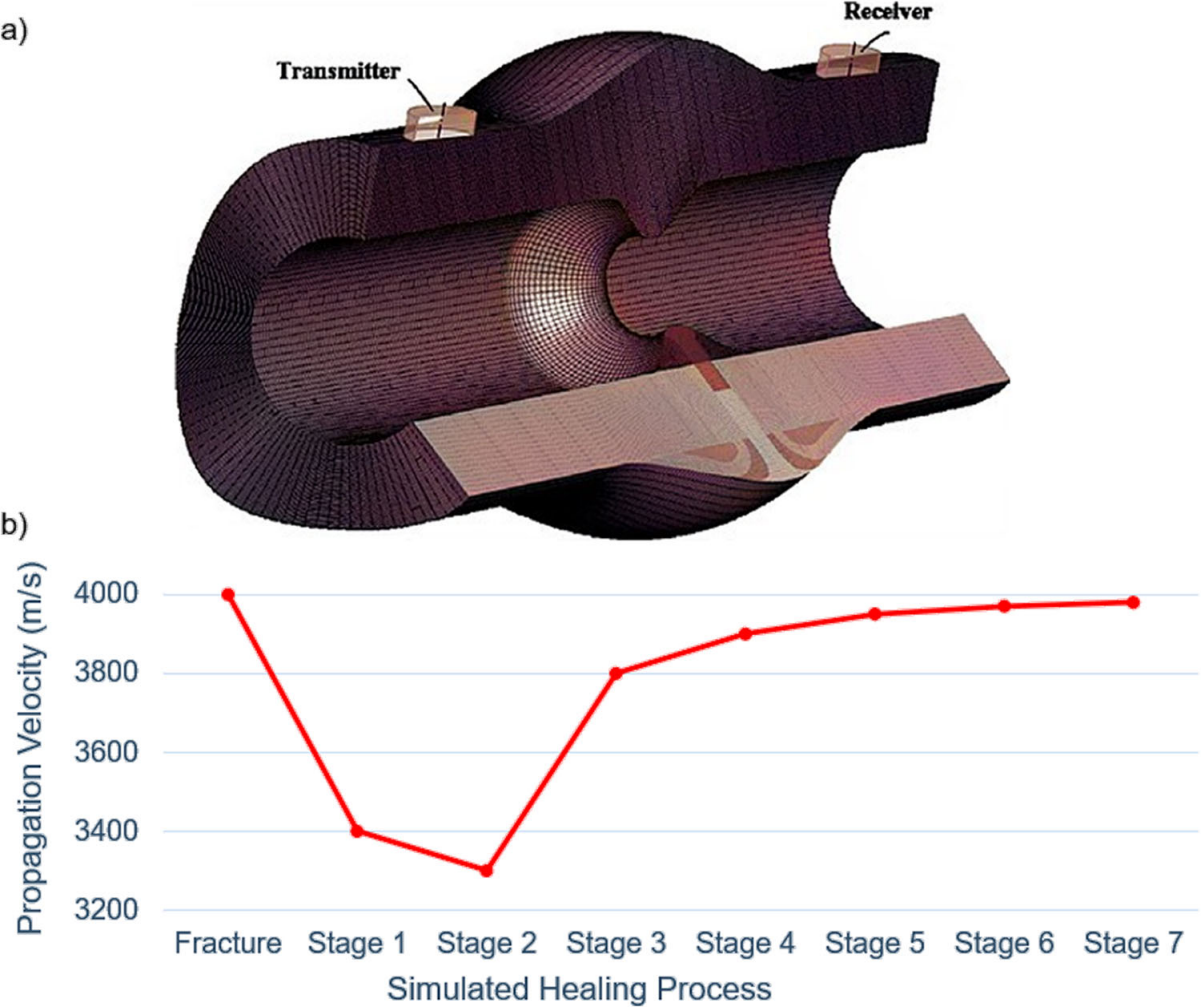
**a** Finite element model of a sheep tibia, combining the fracture callus (sagittal section) and the transmitter–receiver; **b** simulated healing process using ultrasound. Adapted from Protopappas et al. (2008)

Protopappas et al. (2008) simulated the propagation of ultrasonic waves in a healing long bone using 2D and 3D finite difference models. The bone was modeled as an elastic, isotropic plate with a transverse fracture gap filled by callus tissue, whose properties were dynamically updated across seven healing stages, transitioning linearly from blood-like (early hematoma) to cortical bone properties. The axial transmission setup simulated a transmitter–receiver pair operating at 500 kHz and 1 MHz, with a corresponding wavelength of 8 mm and 4 mm in bone, respectively. Simulations allowed to predict that the velocity of the first-arriving signal (FAS), a laterally propagating ultrasonic wave, was decreased from 4063 m/s in intact bone to $\approx$3360 m/s at the first healing stage, remaining low in the second stage, and progressively recovering to over 3900 m/s by the fourth stage (Fig. 18). This trend reflected the increasing stiffness of the callus over time. To further investigate the diagnostic potential of guided waves, time–frequency analysis was also performed on the simulated ultrasonic signals. Previous 2D simulations allowed to find that specific Lamb wave modes, particularly the symmetric S2 and antisymmetric A3 modes, were highly sensitive to the dynamically changing properties and geometry of the callus during healing. In this study, the insights on the sensitivity of specific Lamb wave modes to dynamically changing callus properties were extended to a more realistic 3D model of a sheep tibia, incorporating anisotropy and anatomical geometry. Although time–frequency analysis was not reproduced in the 3D framework, simulations allowed to reveal that higher-order guided modes were significantly affected by both the structural complexity of the measurement site and the anisotropic nature of bone tissue. This suggests that the use of specific longitudinal modes (e.g., L(0,5) and L(0,8)) may offer larger sensitivity for monitoring callus consolidation *in vivo*. However, active biological processes were disregarded, clinical validation was not carried out, and the healing tissues were modeled as simplified structures.

The ultrasound findings are summarized in Table 4, highlighting the model types, biophysical inputs, model characteristics, main predictions, validation method, and limitations related to each study.

**Table 4:** Summary of the model that used ultrasound to monitor and simulate bone healing, emphasizing the detection of ultrasonic wave propagation parameters in bone and their relationship to the healing process

Model type	Biophysical inputs	Model characteristics	Main predictions	Validation method	Limitations	Reference
FDM	Ultrasound-guided waves, soft tissue boundary conditions, evolving callus stiffness	2D finite difference model of bone–plate with multiregion inhomogeneous callus; three healing stages; three boundary condition models (blood immersion, blood+marrow layers); time–frequency RSPWV analysis	FAS velocity increased with healing (e.g., 3730–3900 m/s); higher-order guided modes (e.g., l–A2, l–S3) appeared only under realistic boundary conditions; dispersion patterns sensitive to soft tissue representation	No experimental validation	Simplified 2D plate geometry; no anisotropy or porosity; dispersion analysis for Models 2 and 3 incomplete	Vavva et al. (2007)
FEM	Ultrasonic wave velocity, frequency (500 kHz, 1 MHz), tissue mechanical properties (blood $\rightarrow$ bone)	2D and 3D finite difference models; isotropic and anisotropic bone geometry; fracture gap filled with healing callus in 7 stages; analysis of FAS and Lamb modes	FAS velocity increased with callus stiffening (e.g., 3360 m/s $\rightarrow$ 3900 m/s); guided wave modes were sensitive to geometry and tissue properties; 3D results showed L(0,5) and L(0,8) modes as promising indicators	No validation	No modeling of biological processes; simplified tissue representation	Protopappas et al. (2008)

### Computational models related to dielectric phenomena

While biomechanical modeling remains the predominant approach for simulating bone regeneration, dielectric-based models have emerged as a promising alternative. These models exploit changes in the electrical properties of regenerating tissues across different healing phases, enabling the prediction of capacitive responses from sensors placed near the fracture site. This approach is particularly relevant for the development of low-power implantable devices compatible with autonomous intracorporeal instrumentation (Pires et al. 2025; Conceição et al. 2023).

**Fig. 19:**
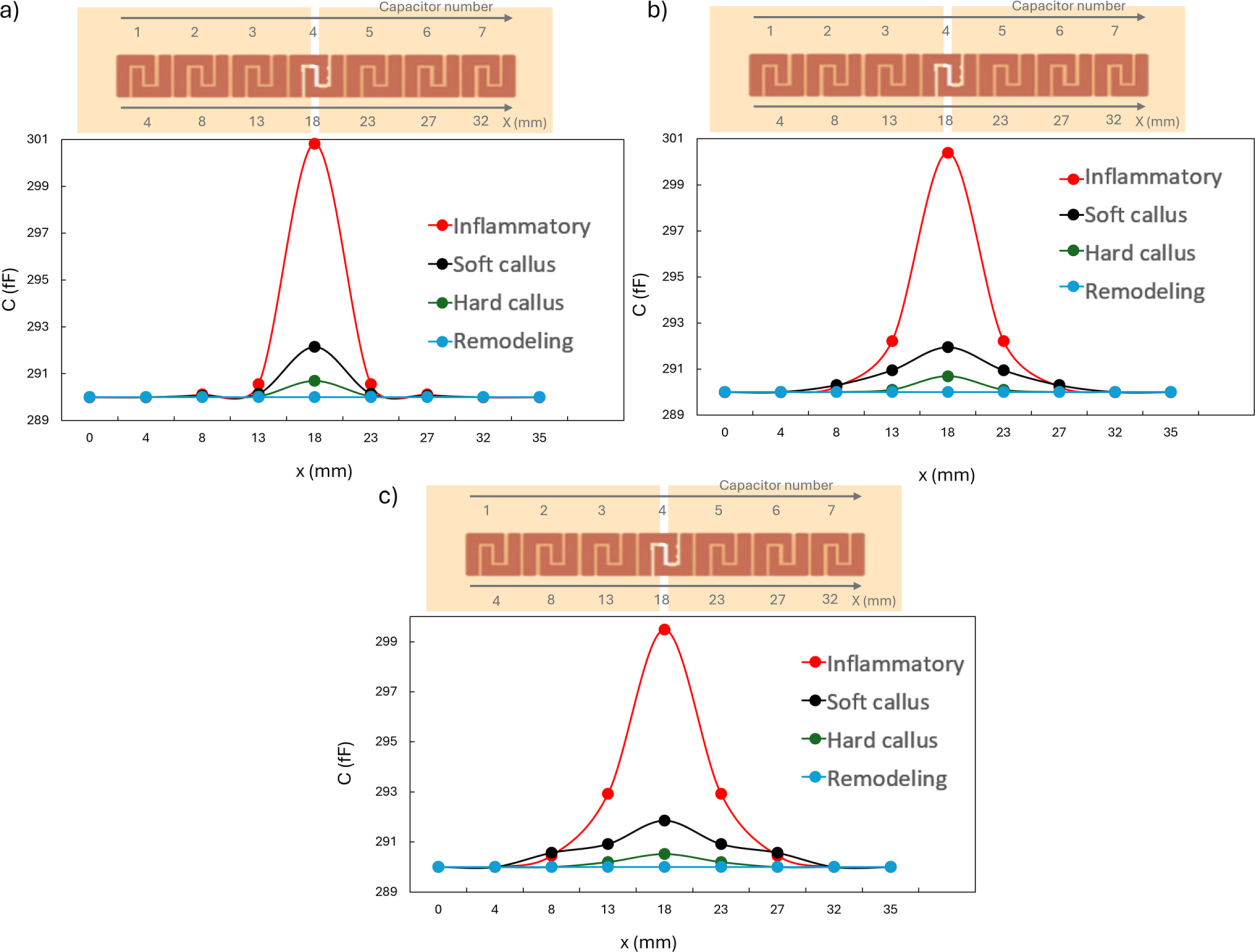
Simulation results showing capacitive response profiles for: **a** 1 mm fracture; **b** 0.5 mm fracture; and **c** 0.25 mm fracture. Interpolated curves were generated using cubic spline fitting to smooth transitions between discrete data points. Adapted from Pires et al. (2025)

Conceição et al. (2023) and Pires et al. (2025) developed a similar dielectric-based finite element model to simulate the capacitive response of an interdigitated sensor placed near a healing bone fracture. The geometry consisted of concentric cylindrical domains representing trabecular and cortical bone (24.56 mm and 30 mm in diameter, respectively, and 40 mm in length), with a 3−0.25 mm wide fracture gap centrally located and filled with a material whose dielectric properties evolved across four healing phases: inflammatory, soft callus, hard callus, and remodeling. These phases were modeled as discrete stages, each with homogeneous isotropic properties derived from literature values. Seven interdigitated electrodes were positioned on a 0.5 mm thick polymeric support at a fixed 2 mm distance from the cortical surface, average deviation distance between an implant and the bone. The system was excited with a sinusoidal voltage of 10 and 3.3 V at 32 kHz, using the Electric Currents interface in COMSOL Multiphysics (v. 6.0, COMSOL Inc.), and the capacitive response was computed for each healing phase. Simulations revealed a progressive decrease in measured capacitance from the inflammatory phase (545.56 fF), soft callus (474.54 fF), and hard callus (464.62 fF) to the remodeling phase (462.92 fF) under a 10 V excitation, corresponding to a total max variation of 17.85%, primarily driven by evolving tissue permittivity and conductivity throughout healing. However, this capacitive contrast was found to diminish under conditions of lower excitation voltages or reduced fracture dimensions. Specifically, decreasing the fracture gap from 1 mm to 0.25 mm resulted in a reduction of peak capacitance variation by approximately 0.9%, with predicted values in the inflammatory phase ranging from 300.82 fF (1 mm) to 299.49 fF (0.25 mm). Similar patterns were observed across all healing stages, particularly in the inflammatory and soft callus phases, which showed the steepest capacitive variations. In contrast, when tissue properties approached those of mature bone (remodeling phase), capacitance variations became negligible, as expected in fully healed structures (Fig. 19). Additionally, for the same 3 mm wide fracture, lowering the excitation voltage from 10 V to 3.3 V further reduced the absolute capacitive response, effectively compressing the dynamic range available to distinguish between healing phases. Under this lower-voltage condition, the maximum variation in capacitance decreased from 17.85% to 10.46%. These findings reinforce the sensitivity of the dielectric model to biological progression but also highlight the influence of sensor configuration and fracture geometry on signal amplitude. Importantly, the relative variation patterns across healing stages remained consistent, supporting the robustness of capacitive sensing as a monitoring approach even in narrower fracture scenarios or low-power settings. Validation against *in vitro* experimental measurements, by mimicking each stage, demonstrated high correlation after normalization ($\hbox {R}^{2}$ ranging from 0.8995 for the remodeling phase to 0.9992 for the inflammatory phase), despite systematic deviations in absolute magnitude attributed to parasitic effects not included in the model. RMSE decreased with tissue consolidation, ranging from 0.016 in the inflammatory phase to 0.0027 in the remodeling phase. Limitations of the model include the use of simplified geometries, absence of osteosynthesis components, and fixed stepwise transitions between tissue types. Furthermore, dynamic biological processes such as cell proliferation or vascularization were not represented, and no mechanical loading conditions were imposed. Nonetheless, the results suggest that dielectric modeling may offer a viable route for non-invasive monitoring of bone healing, especially when integrated into implantable sensor systems capable of capturing subtle changes in local tissue composition. Table 5 provides a comprehensive summary of computational models related to the dielectric phenomena.

**Table 5:** Summary of the dielectric FEM model for capacitive monitoring of bone healing across different stages and conditions

Model type	Biophysical inputs	Model characteristics	Main predictions	Validation method	Limitations	Reference
FEM	Evolving dielectric properties (permittivity, conductivity); excitation voltage (3.3–10 V); fracture width (0.25–3 mm)	3D concentric cylindrical geometry (cortical/trabecular bone + fracture gap); four discrete healing stages; 7 interdigitated electrodes; simulations at 32 kHz using COMSOL	Pick capacitance decreased with healing (545.56 fF at stage 1 to 462.92 fF at stage 4); max variation dropped from 17.85% (10 V) to 10.46% (3.3 V); fracture sizes (0.25–3 mm) do not change sensitivity	*In vitro* capacitive measurements for each stage; high correlation after normalization (R^2^ $> \approx 0.9$); RMSE = 0.0027 at $4^\circ$ stage and 0.016 at $1^\circ$ stage	Simplified geometry; static material transitions; no mechanical load or biological dynamics	Pires et al. (2025), Conceição et al. (2023)

## Discussion

### Mechanical models

Despite considerable progress in developing modeling approaches to predict the dynamics of mechanical factors influencing bone fracture healing, current computational models are limited by several key challenges. Many rely on idealized geometries and simplified material properties, failing to model the biological complexity of the healing process, including cellular dynamics, angiogenesis, and tissue heterogeneity. Moreover, a significant number of these models lack robust experimental validation, which restricts their applicability in clinical settings.

Mechanical stimuli, such as load magnitude, fixation stability, and residual stresses, were found critical for fracture healing. To overcome these limitations and personalize mechanical models, patient-specific imaging (*e.g.,* quantitative CT scans) is the most important data source, since it provides both the 3D fracture geometry and bone density mapping for assigning heterogeneous material properties. Integrating patient-specific mechanical loading history (derived from gait analysis or wearable sensors) is crucial for defining accurate boundary conditions that go beyond idealized static loading. However, proposed models often oversimplify the biological environment, disregarding relevant physiological processes involved in tissue regeneration. Computational approaches should aim to integrate biological complexity with mechanical factors to develop more realistic and predictive simulations. Such advancements hold the potential to optimize the clinical relevance of these models and support personalized treatment strategies to optimize fracture healing outcomes.

### Biological models

Computational models centered on the biological processes of bone healing have significantly advanced our understanding of critical mechanisms such as cellular proliferation and differentiation, angiogenesis, oxygen diffusion, and the influence of mechanical cues on cell behavior. These *in silico* frameworks highlight the essential role of biochemical signaling pathways, immune responses, and vascular dynamics in orchestrating effective tissue regeneration. Moreover, recent efforts have extended biological models to include exogenous stimuli, such as ultrasound and growth factor delivery as potential modulators of healing kinetics.

Despite these advances, important limitations persist. Many models rely on simplified tissue geometries, assume idealized biological conditions, or exclude complex interdependencies between mechanical and molecular regulators. Personalization of biological models requires patient-specific systemic and metabolic data (*e.g.,* age, diseases like diabetes, and blood levels of systemic inflammatory indicators). These clinical features are crucial for calibrating the initial conditions and rate equations regulating cellular proliferation and angiogenesis, which can significantly vary among patient profiles. Direct experimental validation is still lacking in many models, often restricted to qualitative comparisons or constrained parameter spaces. Additionally, few models incorporate patient-specific variability or adaptive feedback mechanisms that characterize real biological systems. Nevertheless, the integration of biological detail into predictive simulations represents a promising step toward optimizing therapeutic strategies. Models that simulate the effects of fixation stability, cytokine gradients, or ultrasound-mediated stimulation have shown the potential to inform clinical decisions and enhance healing outcomes.

### Mechanobiological models

The integration of mechanical and biological factors in bone healing models has considerably advanced our ability to predict tissue regeneration outcomes and optimize treatment strategies. Mechanobiological models provide crucial insight into how local mechanical stimuli regulate cellular behaviors, such as migration, differentiation, proliferation, and matrix deposition. A considerable number of computational approaches were proposed, including poroelastic finite element methods, fuzzy logic, and multiphysics solvers which were employed to simulate the dynamic interplay between biomechanics and biology during healing.

Models consistently demonstrate that both mechanical stability and dynamic loading parameters critically influence the spatial and temporal patterns of tissue differentiation. Calibration for these systems requires a multimodal data strategy due to their integration into two domains. The most important data for personalizing mechanobiological models are the combination of high-resolution imaging modalities (such as MRI for soft tissue mapping and CT for bone structures) to establish the initial morphostructural baseline, and continuous kinematic data to accurately capture the realistic mechanical loading history that drives the differentiation algorithms. Moreover, parameter sensitivity analyses and validation against histological or mechanical data reinforce the ability of these models to replicate experimental specific trends, although full biological validation remains limited in many developed models. Simplifications, such as 2D symmetry, linear elasticity, and static material properties, still constrain prediction accuracy of biological phenomena. Despite these limitations, recent developments, such as incorporating mechanosensitive growth factor transport, scaffold degradation, and phenotype-specific cell behavior highlight a shift toward more comprehensive and realistic simulations. Additionally, machine learning and patient-specific finite element models are beginning to bridge the gap between theoretical predictions and clinical application, enabling personalized rehabilitation protocols and implant design. Future research must prioritize the integration of vascularization, biochemical signaling networks, and adaptive mechanotransduction in dynamically changing geometries. The combined use of experimental data, *in vivo* imaging, and high-performance computing is essential to enhance predictive power and translational relevance.

### Ultrasound models

Ultrasound-based computational modeling has demonstrated strong potential as a method for monitoring bone fracture healing by predicting mechanical changes in callus properties via wave propagation analysis. The proposed models allowed to reveal that both first-arriving signal (FAS) velocity and the emergence of specific guided wave modes (e.g., S2, A3, L(0,5)) correlate with tissue maturation and geometrical complexity during healing. Incorporating realistic soft tissue boundary conditions was found to significantly improved the sensitivity of wave-based indicators, particularly in higher-order modes. To properly adapt these models into specific diagnostic tools, it is important to include the patient’s specific soft tissue morphology and baseline acoustic properties (available through ultrasound methods used in clinical practice). Because wave propagation is highly sensitive to the surrounding soft tissue environment, model calibration using the patient’s specific anatomical boundary conditions is essential to accurately isolate signal changes caused only by bone callus maturation. While these studies laid important groundwork, current models remain limited by simplified bone geometries, lack of anisotropy and porosity, and the absence of biological and clinical validation. Future research integrating patient-specific anatomy and *in vivo* data is essential to translate these simulation tools into clinically relevant monitoring strategies.

### Bioelectric models

Dielectric modeling offers a novel computational approach to monitor bone fracture healing by capturing changes in the electrical properties of regenerating tissues. The reviewed finite element models simulated the capacitive response of interdigitated sensors positioned near the fracture site, taking into account evolving permittivity and conductivity across different healing phases. This approach provides means to assess tissue maturation, particularly in early-stage healing, by detecting dielectric contrasts between inflammatory, cartilaginous, and mineralized tissues. To accurately calibrate and personalize numerical bioelectric models, a critical step is to define the patient’s initial dielectric baseline. The most practical approach to feed these computational frameworks is using data from sensing networks to compare real-time capacitive measurements from the fracture structure with reference sensors on adjacent consolidated bone. This comparative data can provide a dynamic, patient-specific baseline that allows FEM models to continuously update local material properties (such as permittivity and conductivity), ensuring that simulations can accurately predict tissue maturation. Moreover, parametric analyses confirmed that capacitive variation is influenced not only by biological progression but also by practical factors such as fracture gap size and excitation voltage, key parameters in implantable device design. Despite demonstrating high sensitivity and strong agreement with *in vitro* validation data, current dielectric models are still constrained by simplified geometry, fixed material transitions, and the exclusion of active biological processes such as cell migration or vascularization. Additionally, the absence of mechanical loading conditions and implant interference limits the physiological realism of these simulations. Nonetheless, the robustness of capacitive signal patterns across different conditions highlights the potential of dielectric sensing for continuous and minimally invasive healing assessment.

### Challenges and future opportunities in AI-driven models

The integration of AI and data-driven algorithms into orthopedic research provides unique capabilities. These algorithms excel at processing large datasets and detecting complex nonlinear patterns that can hardly be found by human capabilities, considerably improving fracture healing prognosis and individualized treatment planning (Song et al. 2025). However, despite these potential benefits, several limitations hinder its acceptance in clinical practice. A key constraint is data availability and quality. The use of neural networks, in particular deep learning, require massive datasets to ensure accurate predictions and avoid underfitting. Fragmented data are often unstructured or limited by privacy restrictions (Misir and Yuce 2025). Furthermore, generalization remains a major challenge, as models trained on homogeneous patient groups may fail when applied to heterogeneous patient groups, namely for different demographic characteristics, age and gender (Lisacek-Kiosoglous et al. 2023). Furthermore, the "black box" architecture of many AI systems challenges the confidence level demanded by clinicians. Interpretability is vital, as clinicians need to understand which are the methods and algorithms behind the prediction process. Consequently, it is crucial to emphasize that AI should not be considered an isolated alternative to medical expertise. These models hold the potential to be implemented as clinical decision support systems (CDSSs), supporting healthcare providers in making more informed and data-driven decisions, rather than replacing human judgment (Song et al. 2025; Misir and Yuce 2025). Finally, future efforts must focus on prospective, multicenter trials to realistically validate the safety and reliability of these tools before their widespread use (Kumar et al. n.d.).

The most promising opportunity most likely lies in the development of hybrid models integrating the predictive power of AI with the mechanistic principles of finite element analysis (FEA). Unlike purely data-driven techniques, these hybrid frameworks can enforce physical principles during the learning process, possibly solving data shortage problems by producing synthetic training data (Misir and Yuce 2025; Lisacek-Kiosoglous et al. 2023). Furthermore, the convergence of real-time sensor data, patient-specific imaging, and computational modeling is paving the way for Digital Twins in orthopedics, including in the scope of fracture healing. These virtual replicas hold the potential to continuously be updated based on the patient’s rehabilitation progress, offering a dynamic and personalized roadmap for fracture management that adapts to complications in real time.

### Promising modeling approaches

To bridge the gap between computational research and clinical application, particularly in the treatment of delayed healing and non-union fractures, modeling methodologies capable of integrating real-time patient-specific monitoring. The ultimate goal is to provide a transition from static to dynamic predictions. As a result, we believe that the most promising approach for near-term clinical translation is most likely to combine robust mechanobiological frameworks with monitoring technologies, such as bioelectronics (Pires et al. 2025; Conceição et al. 2023). The mechanobiological frameworks proposed by Perier-Metz et al. (2022) and Liu et al. (2023) are the most robust computational baselines among the reviewed contributions. These models accurately reproduce tissue differentiation rules under different mechanical loads. However, their clinical potential can be strongly explored to operate as a prediction engine that uses data from external sources to dynamically update tissue material properties, rather than depending exclusively on theoretical mechanobiological initial conditions. To provide real-time physiological feedback, the dielectric modeling approaches proposed by Pires et al. (2025) and Conceição et al. (2023) are promising in fracture healing. Indeed, their findings show that capacitive sensing of dielectric variations may be used to detect physical changes in tissue mechanobiological properties. Indeed, several physiological factors influence the dielectric behavior of bone-associated tissues during healing (Tannino et al. 2023), including: (i) hydration levels: changes in tissue hydration significantly affect electrical conductivity and permittivity; (ii) collagen and mineral content: progressive mineralization changes tissue impedance and dielectric constant; (iii) cellular density and activity: proliferation and differentiation of osteoblasts and other cells modify the electrical properties of the regenerating tissue. These bioelectrical signals provide essential input data for calibrating and validating the mechanobiological models discussed earlier. By using prediction data of dielectric dynamics (timely updated by monitoring systems, as proposed by Pires et al. (2025)) to supply mechanobiological simulations (as proposed by Perier-Metz et al. (2022)), a closed feedback loop systems can be implemented for enhancing phenomena predictability. This approach would allow early and more accurate predictions of non-unions by deviations from the expected mechanobiological trajectories, enabling timely and more effective clinical interventions based on bioelectric data rather than time-consuming and subjective imaging analyses.

## Conclusion

Bone fracture healing is a complex process governed by mechanical, biological, and biochemical factors. Over the years, computational modeling has played a crucial role in elucidating this phenomenon. These models, mainly categorized as mechanical, biological, and mechanobiological, have provided important insights into the cellular and tissue-level dynamics of bone regeneration.

Mechanical models were developed to be primarily focus on external forces, stress distributions, and vibrational stimuli. They allowed to assess how these factors influence callus development, tissue differentiation, and mechanical stability. However, proposed models often neglect physiological and metabolic processes, limiting their ability to predict long-term biological outcomes. Differently biological models emphasize cellular proliferation, angiogenesis, oxygen transport, and the action of growth factors. Although they hold the potential to predict molecular and cellular mechanisms, they generally overlook the influence of the mechanical environment in modulating these responses. Mechanobiological models, such as those proposed by Isaksson et al. in 2008 Isaksson et al. (2008) and Repp et al. in 2015 Repp et al. (2015), offer a more integrated perspective. These frameworks explicitly couple mechanical stimuli to cellular behaviors and tissue formation, thereby bridging biomechanics and biology. Nonetheless, even these advanced models tend to disregard the temporal evolution of key physical tissue properties mainly the electrodynamics found in mechanobiological processes

A significant limitation found in the reviewed literature is the limited number of modeling approaches related to electric phenomena occurring in tissues surrounding the fracture site. These properties, affected by tissue hydration, collagen content, and cellular density (Tannino et al. 2023), are dynamically changed during healing, being sensitive indicators of tissue maturity and integrity. Despite their diagnostic potential, bioelectrical variables remain largely neglected from existing computational frameworks. Addressing this gap requires the integration of dynamic dielectric properties into future modeling approaches. Incorporating electrical characteristics alongside mechanical and biological variables could significantly enhance the predictive accuracy and clinical relevance of such models. For instance, the work of Pires et al. (2025) and Conceição et al. (2023) demonstrates how capacitance measurements of dielectric properties can reflect changes in tissue composition over time, offering a new avenue for monitoring healing progression. Furthermore, advances in ML and patient-specific modeling, such as those presented by Liu et al. (2023), could be leveraged to analyze and predict variations in dielectric properties with higher precision, such as hydration, mineral content, and cellular density, across distinct healing phases (Tannino et al. 2023).

Incorporating these factors requires robust experimental datasets and sophisticated multiscale modeling approaches. Existing frameworks, such as those developed by Quinn et al. (2022) and Perier-Metz et al. (2022), offer a valuable support that can be extended to include bioelectrical properties.

Among the reviewed contributions, the following are particularly promising for future research in dielectric modeling: (a) Perier-Metz et al. (2022) and Liu et al. (2023): their mechanoregulation model can be enhanced to incorporate dielectric dynamics as a function of cellular activity; (b) Pires et al. (2025) and Conceição et al. (2023): their dielectric models demonstrate how capacitive sensing can track temporal changes in tissue dielectric properties across different healing phases, reinforcing the potential of bioelectrical signals as complementary biomarkers in bone regeneration.

By combining mechanical, biological, and bioelectrical modeling paradigms, future research can yield more dynamic and realistic simulations of bone fracture healing. Although current computational models have significantly improved our understanding of such healing process, their inability to account for the evolving dielectric landscape of tissues remains a significant limitation. Bridging this gap would not only enhance predictive performance, but also enable the development of advanced, non-invasive diagnostic tools and bioelectronic implants. Ultimately, such innovations hold the potential to offer effective personalized treatment strategies, while reducing the reliance on animal models for testing and validation.

## Data Availability

No datasets were generated or analyzed during the current study.
